# Human Antibodies Fix Complement to Inhibit *Plasmodium falciparum* Invasion of Erythrocytes and Are Associated with Protection against Malaria

**DOI:** 10.1016/j.immuni.2015.02.012

**Published:** 2015-03-17

**Authors:** Michelle J. Boyle, Linda Reiling, Gaoqian Feng, Christine Langer, Faith H. Osier, Harvey Aspeling-Jones, Yik Sheng Cheng, Janine Stubbs, Kevin K.A. Tetteh, David J. Conway, James S. McCarthy, Ivo Muller, Kevin Marsh, Robin F. Anders, James G. Beeson

**Affiliations:** 1 The Burnet Institute for Medical Research and Public Health, 85 Commercial Road, Melbourne, VIC 3004, Australia; 2 Department of Medical Biology, University of Melbourne, Royal Parade, Melbourne, VIC 3010, Australia; 3 Centre for Geographic Medicine Research, Kenya Medical Research Institute, Coast, PO Box 230, 80108 Kilifi, Kenya; 4 London School of Hygiene and Tropical Medicine, Keppel Street, London WC1E7HT, UK; 5 QIMR Berghofer Medical Research Institute, University of Queensland, 300 Herston Road, Herston, QLD 4006, Australia; 6 Walter and Eliza Hall Institute, Royal Parade, Melbourne, VIC 3050, Australia; 7 Department of Biochemistry, La Trobe Institute for Molecular Science, La Trobe University, Melbourne, VIC 3086, Australia; 8 Department of Microbiology, Monash University, Clayton, VIC 3800, Australia

## Abstract

Antibodies play major roles in immunity to malaria; however, a limited understanding of mechanisms mediating protection is a major barrier to vaccine development. We have demonstrated that acquired human anti-malarial antibodies promote complement deposition on the merozoite to mediate inhibition of erythrocyte invasion through C1q fixation and activation of the classical complement pathway. Antibody-mediated complement-dependent (Ab-C′) inhibition was the predominant invasion-inhibitory activity of human antibodies; most antibodies were non-inhibitory without complement. Inhibitory activity was mediated predominately via C1q fixation, and merozoite surface proteins 1 and 2 were identified as major targets. Complement fixation by antibodies was very strongly associated with protection from both clinical malaria and high-density parasitemia in a prospective longitudinal study of children. Ab-C′ inhibitory activity could be induced by human immunization with a candidate merozoite surface-protein vaccine. Our findings demonstrate that human anti-malarial antibodies have evolved to function by fixing complement for potent invasion-inhibitory activity and protective immunity.

## Highlights

• Antibodies function with complement to inhibit *P. falciparum* replication
• Antibodies fix C1q to block invasion and lyse merozoites
• Complement-fixing antibodies are strongly associated with immunity in children
• Antibody-complement inhibition can be induced by human vaccination

## Graphical Abstract

**Figure:**
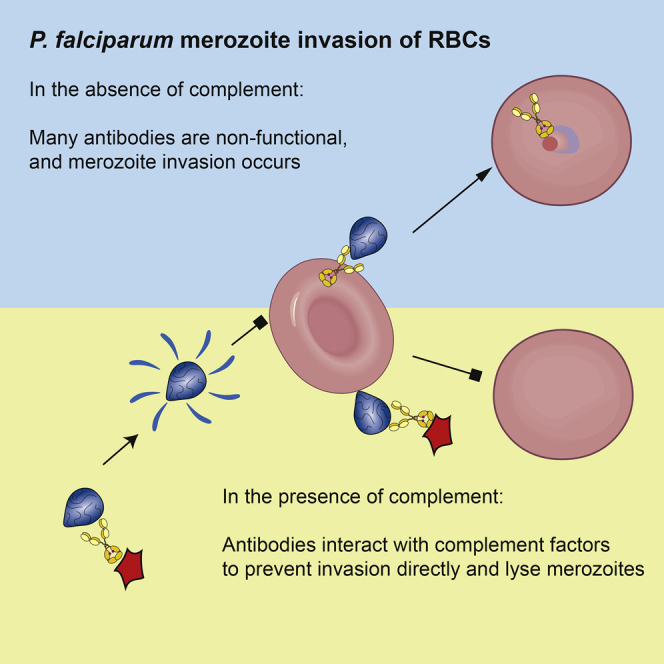

## Teaser

Antibodies are important in immunity to malaria, but their protective function has been unclear. Boyle and colleagues report that acquired and vaccine-induced human antibodies recruit complement to block infection of erythrocytes and blood-stage replication of *Plasmodium falciparum*.

## Introduction

Humoral responses to *Plasmodium falciparum* are an important component of acquired immunity against malaria, as demonstrated in pivotal studies in which immunoglobulin G (IgG) from immune adults was transferred to malaria-infected children and resulted in parasite clearance and recovery (Cohen et al., 1961). Antibodies are thought to protect by inhibiting blood-stage replication and preventing high-density parasitemia. However, specific mechanisms of protection are not well understood. The merozoite stage, which infects red blood cells (RBCs), is an important target, and antibodies to some merozoite antigens can inhibit *P. falciparum* replication in vitro (Hodder et al., 2001; Miura et al., 2009; Reiling et al., 2012; Wilson et al., 2011). However, antibodies targeting numerous merozoite antigens, including vaccine candidates such as MSP2 and MSP3, lack activity in these standard assays (McCarthy et al., 2011; Oeuvray et al., 1994), despite some evidence of efficacy in clinical and pre-clinical trials (Genton et al., 2002; Sirima et al., 2011). Indeed, growth-inhibitory activity of human antibodies is not consistently predictive of clinical immunity (Crompton et al., 2010; Dent et al., 2008; Marsh et al., 1989; McCallum et al., 2008), and antibodies from immune adults often fail to inhibit parasite replication in standard assays (Dent et al., 2008; McCallum et al., 2008; Shi et al., 1999). A lack of established immune correlates of protection severely hampers the evaluation and prioritization of vaccines (Beeson et al., 2014).

Overall reactivity of antibodies to merozoite antigens as measured by ELISA correlates with protection in some, but not all, human studies (Fowkes et al., 2010). Human antibodies to merozoite antigens are predominantly cytophilic subclasses IgG1 and IgG3; these have been associated with protection from malaria (Polley et al., 2006; Richards et al., 2010; Roussilhon et al., 2007; Stanisic et al., 2009; Taylor et al., 1998). This raises the question of whether complement might be an important effector of antibody function. Although complement activation has been reported in malaria infection and innate activation has been implicated in pathogenesis (reviewed in Biryukov and Stoute, 2014), the role of complement in antibody-mediated protection has not been defined.

Here, we developed approaches and assays to determine the ability of acquired human antibodies to fix complement and inhibit merozoite invasion of RBCs and to identify major merozoite targets of these antibodies. We evaluated antibody activity in naturally exposed individuals from diverse geographic regions and vaccinated humans, and we obtained epidemiologic evidence supporting a role for antibody-mediated complement fixation in protective immunity to malaria in children. Our findings represent a major advance in understanding immunity to malaria and provide a much-needed strategy for the development and evaluation of vaccines.

## Results

### Human IgG from Malaria-Exposed Donors Has Complement-Dependent Inhibitory Activity

**Figure 1:**
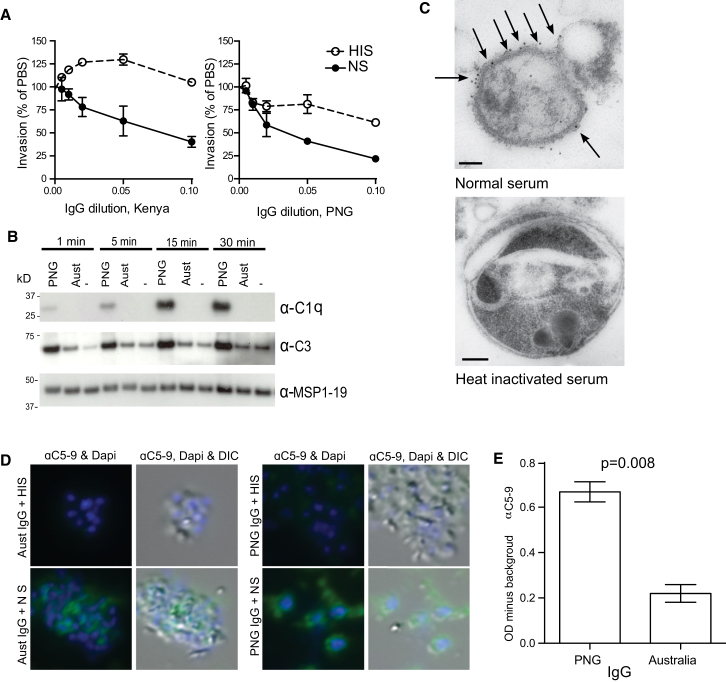
Invasion Inhibition by IgG and Complement and Complement Deposition on the Merozoite Surface (A) Invasion-inhibitory activity of purified IgG from Kenya and PNG was tested in invasion assays performed with 50% normal serum (NS; complement active) or heat-inactivated serum (HIS; complement inactivated). Data represent the mean ± range from two independent assays performed in duplicate. (B) C1q and C3 deposition on merozoites incubated with purified PNG IgG, purified malaria-naive IgG (Australian donors), or PBS together with 25% NS for 1, 5, 15, and 30 min. MSP1-19, a merozoite surface protein, was used as a loading control. (C) C3b deposition on merozoites incubated with purified PNG IgG and 25% NS or HIS via immuno-electron microscopy. Gold labeling is indicated with arrows. Scale bars represent 0.1 μm. (D) Formation of the membrane attack complex (MAC; complement components C5–C9) on merozoites incubated with purified PNG or Australian (Melbourne) IgG and 25% NS or HIS via IF microscopy. (E) MAC deposition as quantified by ELISA on merozoites incubated with NS and PNG or Australian IgG. Immunoblots and microscopy images are representative of two independent experiments. See also Figure S1.

To assess the role of complement in antibody inhibition of invasion, we performed merozoite-invasion assays in the presence or absence of active complement (Boyle et al., 2010b; Figures S1A and S1B). Merozoites were isolated from schizonts via membrane filtration and incubated with uninfected RBCs together with increasing concentrations of purified IgG (1/200 to 1/10 dilution) from malaria-exposed pooled donors (from Kenya and Papua New Guinea [PNG]) in the presence of either normal serum (NS; complement active) or heat-inactivated serum (HIS; complement inactive). IgG from Kenyan donors was non-inhibitory in HIS but effectively inhibited invasion when incubated with NS (Figure 1A). IgG from PNG donors had some activity in HIS, but inhibition was much greater in NS (Figure 1A). IgG from malaria-naive donors (Australian residents) was not inhibitory in NS or HIS, and the fact that NS did not inhibit in the absence of IgG indicates that complement alone is non-inhibitory (Figures S1C and S1D). The greater inhibition of merozoite invasion by malaria-exposed IgG in NS than in HIS suggests that IgG interacts with complement to inhibit invasion. This identifies an invasion-inhibitory mechanism that we refer to as antibody-mediated complement-dependent (Ab-C′) inhibition. We will refer to inhibitory activity of antibodies in the absence of complement (HIS) as direct antibody inhibition.

Complement fixation on merozoites incubated with malaria-exposed IgG (PNG residents) or malaria-naive IgG (Australian residents) was investigated via immunoblot. C1q and C3b were detected at higher levels on merozoites incubated with NS and PNG IgG than on merozoites incubated with Australian IgG, reflecting activation of the classical complement cascade by anti-merozoite antibodies (Figure 1B; Figures S1E and S1F). Some C3b deposition was detected on merozoites incubated with Australian IgG and NS, suggesting activation of the antibody-independent alternate pathway. C3b deposition was confirmed by immuno-electron microscopy; merozoite surface-bound C3b was detected after incubation with PNG IgG and NS, but not with HIS (Figure 1C; Figure S1G). The formation of the membrane attack complex (MAC; components C5–C9) was detected by immuno-fluorescence (IF) microscopy. MAC formation was detected on merozoites incubated with PNG IgG and NS. Lower MAC formation was seen with Australian IgG and NS (Figure 1D). Using an ELISA-based assay, we quantified MAC deposition and demonstrated that it was significantly higher (p < 0.01) with PNG IgG than with Australian (Melbourne) IgG (Figure 1E). These data show that human anti-malarial antibodies enhance complement deposition on merozoites via C1q fixation and thus result in increased C3b deposition and MAC formation.

### C1q Fixation Mediates Ab-C′ Inhibition

**Figure 2:**
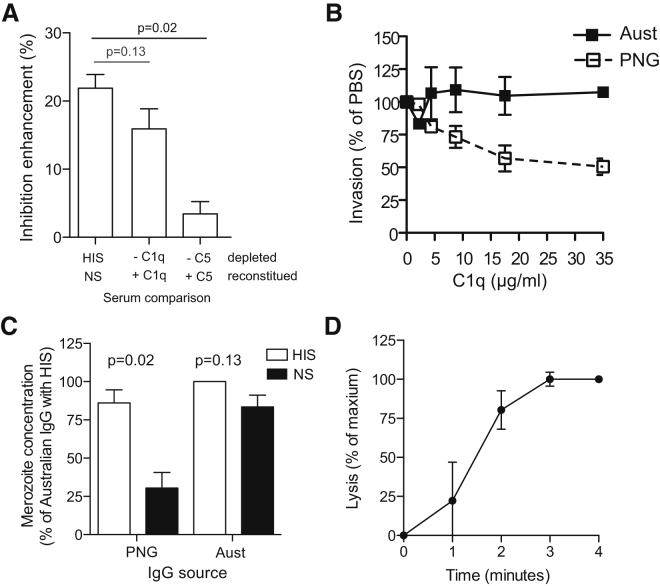
C1q Fixation by IgG Inhibits Invasion, and Complement Fixation Leads to Merozoite Lysis (A) Invasion-inhibitory activity of purified PNG IgG (1/10 dilution) was tested in the presence of 25% NS and HIS, C1q-depleted serum with and without reconstitution with purified C1q, and C5-depeleted serum with and without reconstitution with C5. The difference in invasion-inhibitory activity between depleted and reconstituted serum was calculated. Data represent the mean ± SEM from four to five independent assays performed in duplicate. (B) Invasion-inhibitory activity of purified PNG IgG in the presence of increasing concentrations (2.2–35 μm/ml) of purified C1q. Data show invasion as a percentage of that of media alone and represent the mean ± range from two independent assays performed in duplicate. (C) Lysis of merozoites: merozoite concentration after 10-min incubation with 1/20 dilution of purified PNG or purified malaria-naive Australian IgG and 20% NS or HIS. Data show merozoite concentration as a percentage of that of purified Australian IgG with HIS and represent the mean ± SEM from three independent assays performed in duplicate. (D) Lysis rate of merozoites incubated with purified PNG IgG and 20% NS. Data show lysis as a percentage of the maximum and represent the mean ± SEM from four independent assays. See also Figure S2.

To test the importance of the activation of the classical complement cascade in relation to that of the alternative complement cascade, we compared the invasion-inhibitory activity of anti-malarial antibodies between NS and HIS heated at 50°C for 20 min; this treatment inhibits the alternative complement cascade by inactivating Factor B but leaves the classical complement cascade intact. The 50°C treatment of serum had no significant effect on the invasion-enhancing activity; invasion inhibition by PNG IgG was greater in the presence of NS and 50°C-treated serum than in the presence of standard HIS (Figure S2A). This indicates that amplification of the alternative pathway does not account for Ab-C′ inhibition. To address the relative importance of different complement components, we tested invasion inhibition in C1q- and C5-depleted serum and reconstituted serum. There was significant enhancement of invasion inhibition by PNG IgG in assays with C1q-reconstituted serum in comparison to assays with C1q-depleted serum, which was comparable to the greater inhibition in NS than in HIS (Figure 2A). In contrast, there was significantly less of a difference in the extent of PNG IgG inhibition in C5-reconstituted serum than in C5-depleted serum. This suggests that activation of the classical complement cascade, specifically fixation of C1–C4, might be sufficient to mediate the majority of Ab-C′ and that deposition of C5–C9 is of less importance. To investigate this further, we incubated merozoites with PNG or Australian IgG in increasing concentrations of purified human C1q in the absence of other complement factors. C1q substantially enhanced the inhibitory activity of PNG IgG (Figure 2B), indicating that C1q fixation alone was sufficient to mediate substantial Ab-C′ inhibition. No inhibition was seen in the presence of control IgG or C1q.

To investigate whether MAC deposition and lysis could also contribute to inhibiting invasion, we assessed the ability of IgG and complement to lyse merozoites. Merozoite invasion occurs rapidly, such that 80% of invasion occurs within 10 min of mixing with RBCs (Boyle et al., 2010b); therefore, we assessed the lysis of merozoites within this time period. Merozoites were incubated with PNG or Australian IgG and 20% NS or HIS and counted by flow cytometry (Figure 2C; Figure S2B). PNG IgG mediated a significantly greater reduction in merozoites in NS than in HIS, whereas there was little lysis of merozoites with Melbourne IgG. Over an extended time period (30–60 min), lysis of merozoites was observed in NS alone (Figure S2C), consistent with the low rate of MAC formation in the absence of malaria-specific IgG (Figure 1E). Rapid merozoite lysis was dependent on activation of the classical complement cascade and was not an artifact of agglutination, as confirmed by the lack of lysis in C1q-depleted serum (Figure S2D). Using PNG IgG and 20% NS, analysis of the timing of merozoite lysis revealed that lysis occurred rapidly, such that the majority occurred within 2–3 min of incubation and reached a maximum by 4 min (Figure 2D).

### Ab-C′ Inhibition Is the Predominant Mechanism Targeting Merozoite Invasion

**Figure 3:**
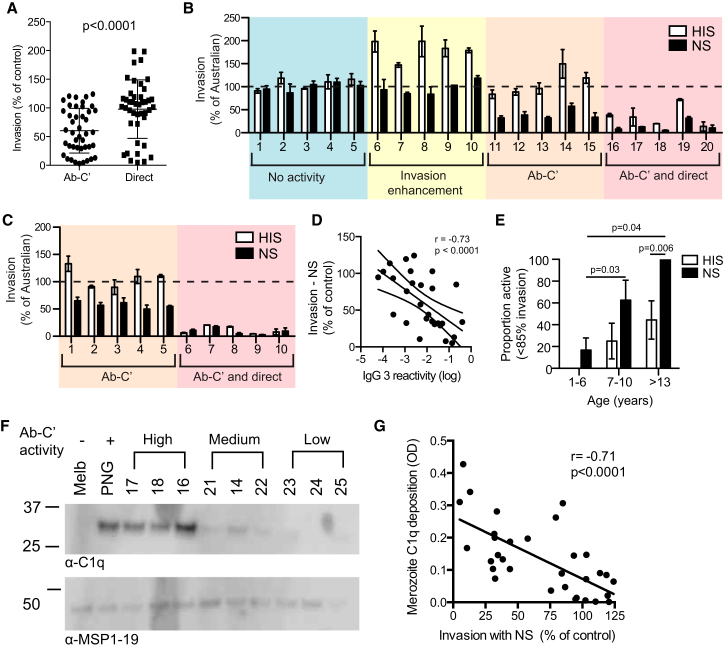
Ab-C′ Is the Predominant Mechanism of Naturally Acquired Antibodies and Correlates with C1q-Deposition Activity (A) Invasion-inhibitory activity of purified IgG from Kenyan and PNG individuals in 50% NS or HIS (median ± interquartile range). (B) Invasion-inhibitory activity of purified IgG from Kenyan individuals in the presence of NS and HIS; shown are no inhibitory activity (blue), invasion-enhancement activity (in HIS and not NS, yellow), Ab-C′ inhibition (orange), and Ab-C′ and direct inhibitory activity (red). Data represent the mean ± range from two independent assays performed in duplicate. (C) Invasion-inhibitory activity of purified IgG from PNG individuals. (D) IgG3 reactivity to the merozoite surface as measured by ELISA correlated with functional activity in Ab-C′ assays. Data show invasion as a percentage of that of the control. (E) Significant activity in invasion-inhibition assays with 50% NS or HIS was defined as <85% invasion (>15% inhibition); data show the proportion of individuals with activity, and individuals are stratified by age. p values are shown for comparisons of the proportion of samples that were positive in assays using NS between the 1–6 and 7–13 age groups and between the 1–6 and >13 age groups. The p value is also shown for the comparison of the proportion of positive samples in the >13 age group between assays using NS and assays using HIS. (F) C1q deposition on merozoites was assessed by immunoblot for nine purified Kenyan IgG samples that had high, medium, or low Ab-C′ inhibitory activity. The image is representative of two independent assays. Abbreviations are as follows: Melb, pool of Melbourne IgG included as a negative control; PNG, pool of PNG IgG included as a positive control. (G) Measured by ELISA, C1q deposition on merozoites in purified Kenyan IgG samples correlated with Ab-C′ inhibitory activity (n = 33). See also Figure S3.

The importance of Ab-C′ inhibition in naturally acquired immunity was assessed with purified IgG from Kenyan (n = 33) and PNG (n = 10) individuals. Overall, there was much greater Ab-C′ inhibition than direct inhibitory activity (Figure 3A). Compared to direct inhibition, Ab-C′ inhibitory activity was seen in a much greater proportion of individuals (proportion positive [defined as >15% inhibition]: 57.2% ± 8.7% for Ab-C′ and 21.2% ± 7.2% for direct inhibition; p < 0.01). These striking results reveal that the majority of human antibodies require complement factors to effectively inhibit merozoite invasion. The extent of inhibitory activity varied widely. In Kenyan individuals, four activity profiles were observed: (1) no inhibitory activity in NS or HIS (11/33 [33%]); (2) invasion enhancement in HIS, but not NS (7/33 [21%]); (3) Ab-C′ inhibition only, demonstrated by invasion inhibition in NS, but not HIS (10/33 [30%]); and (4) a combination of Ab-C′ inhibition and direct inhibitory activity, demonstrated by inhibition in HIS and increased inhibition in NS (5/33 [15%]) (Figure 3B; Figure S3A). Among PNG individuals, 50% had Ab-C′ inhibitory activity only, and the remaining samples had substantial direct inhibitory activity (Figure 3C). Overall, 37% of samples had only complement-dependent inhibitory antibodies. In those with both complement-dependent and directly active antibodies, complement-dependent inhibition ranged from 12% to 81% of the total inhibitory response.

#### Ab-C′ Inhibition Strongly Correlates with Cytophilic Antibodies to Merozoites

In Kenyan individuals, we assessed the relationship among Ab-C′ inhibition, direct inhibition, IgG subclass reactivity to merozoites, and antibody activity in standard complement-free growth-inhibition assays (GIAs). The prevalence of antibodies to merozoites was high, and merozoite-specific IgG was strongly and significantly correlated with Ab-C′ inhibition, but not with direct inhibitory activity and less strongly with activity in GIAs (Table 1). Of note, the strongest correlation was between Ab-C′ inhibition and IgG3 (Figure 3D). This relationship was stronger than that seen for IgG1 and is consistent with the known property of IgG3 as the most potent activator of complement. Ab-C′ inhibition was also strongly correlated with age (Spearman’s r = 0.63, p = 0.0003), matching the acquisition of immunity. In contrast, direct inhibitory activity only weakly correlated with age (Spearman’s r = 0.17, p = 0.38). With a median invasion in NS of 32.3% (95% CI = 54.7–82.2), Ab-C′ activity was also greater than activity in GIAs, whose median growth was 89.3% (95% CI = 81–91, p = 0.007). Further, the proportion of individuals with positive Ab-C′ inhibition increased with age, and 100% of adults had Ab-C′ inhibitory activity; only 44% had direct activity (Figure 3E). These results strongly suggest that Ab-C′ is the predominant mechanism of antibodies targeting merozoite invasion and is acquired in naturally exposed individuals as immunity to malaria develops.

#### C1q Fixation by Antibodies Correlates with Ab-C′ Inhibition

Having shown that Ab-C′ inhibition functions via fixation of C1q and the activation of the classical complement cascade, we evaluated the relationship between C1q deposition and Ab-C′ inhibition. We measured antibody-mediated C1q deposition on merozoites by immunoblot using IgG from nine Kenyan individuals with high, medium, or low Ab-C′ inhibition (n = 3 for each group). C1q deposition was notably higher with IgG from individuals who had high Ab-C′ inhibitory activity than with IgG from those with medium or low activity (Figure 3F). Next, to quantify C1q fixation on merozoites, we developed a high-throughput plate-based assay that uses small sample volumes to allow testing of large numbers of serum samples and that can be used with small-volume pediatric samples (Figure S3B). Antibody-mediated C1q deposition on merozoites was strongly correlated with Ab-C′ activity (Figure 3G). Samples that promoted high C1q fixation also had high C3b fixation (Figure S3C). This supports the important role of C1q fixation and activation of the classical cascade in Ab-C′ inhibition and establishes an efficient complement-deposition assay that is suitable for application to clinical studies.

#### Antibodies that Fix Complement Are Associated with Protection from Malaria

**Figure 4:**
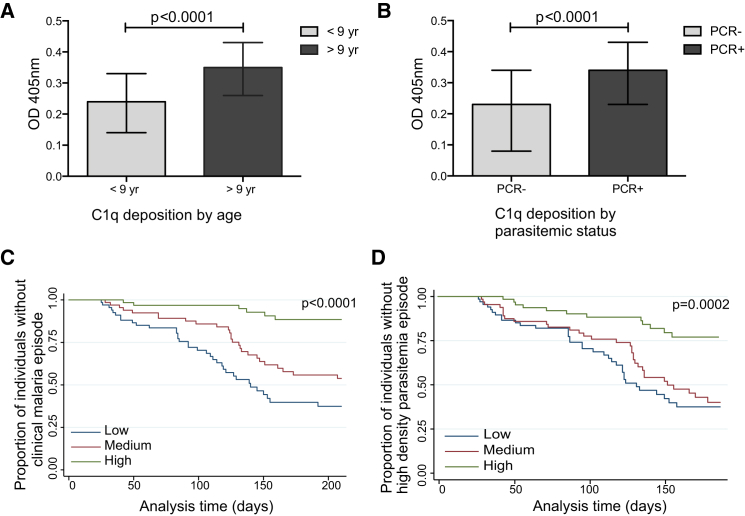
C1q Fixation by Antibodies Is Associated with Protection from Malaria Antibody-mediated C1q deposition on the merozoite surface was measured in plasma from a cohort of 206 children in PNG. (A) C1q deposition was higher in older children (>9 years; n = 115). (B) C1q deposition was higher in children with concurrent *P. falciparum* infection (n = 139) than in uninfected children, as determined by PCR. (C) High C1q fixation by antibodies was strongly associated with reduced risk of clinical malaria episodes. Children were divided into three groups on the basis of high, medium, and low C1q-fixing antibodies. (D) High antibody-mediated C1q fixation was associated with reduced risk of high-density parasitemia. See also Figure S4.

To obtain epidemiologic evidence of the importance of antibody-mediated complement fixation in acquired immunity to malaria, we tested antibodies for C1q fixation from a longitudinal cohort of 206 5- to 14-year-old children who were resident in a malaria-endemic region of PNG (Michon et al., 2007); all children were treated for malaria parasitemia at enrollment and then monitored by active surveillance for parasitemia and clinical malaria over 6 months of follow-up. The prevalence of antibody-mediated C1q fixation on the merozoite surface was very high (Table S1), reflective of substantial exposure to malaria in this population. Antibody-mediated C1q deposition was associated with age, such that older children had significantly higher C1q deposition than younger children (Figure 4A; Figure S4). C1q deposition was also higher in children who were parasitemic at the time of sample collection than in aparasitemic children (Figure 4B). The increase in antibody-dependent C1q deposition with age and parasitemia is consistent with the expected acquisition of immunity.

To assess the role of antibody-mediated C1q fixation in protection from symptomatic malaria and high-density parasitemia, we grouped children into low, medium, and high categories of C1q-fixation activity; we then compared the relative risk of malaria between response groups. High C1q deposition was very strongly associated with protection from clinical malaria (Table 2). The association between antibodies and protection from malaria appeared to have a dose-response relationship (Figure 4C); high responders had fewer symptomatic episodes than did medium or low responders. Numerous variables were explored as possible confounding factors; only subject age and location of residence were significantly associated with risk of malaria (Michon et al., 2007). The strong protective association for antibody-mediated C1q deposition remained after adjustment for age and location of residence (p < 0.0001), and protective associations were observed for children who were parasitemic or aparasitemic at enrollment (Figure S4B). High C1q deposition was also strongly associated with protection from episodes of high-density parasitemia (>5,000 parasites/μl), which remained significant after adjustment for confounders (Table 2; Figure 4D). These data are consistent with a role for antibody-mediated complement fixation and Ab-C′ inhibition in limiting blood-stage replication of *P. falciparum* and preventing disease.

### MSP1 and MSP2 Are Targets of Ab-C′ Inhibitory Antibodies

**Figure 5:**
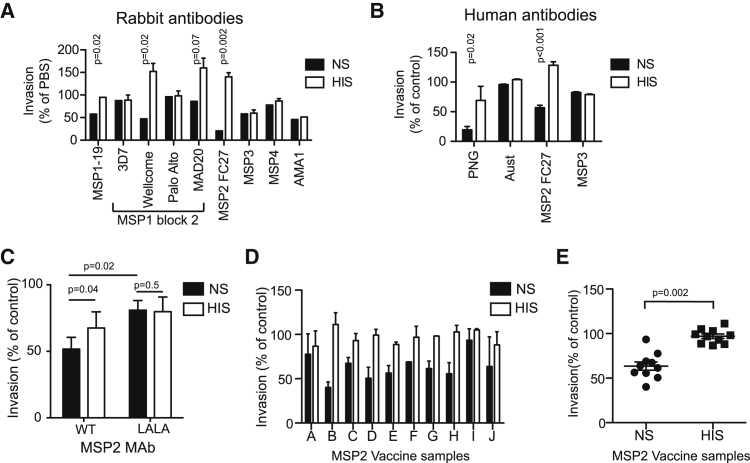
MSP1 and MSP2 Are Targets of Ab-C′ Inhibition, and Ab-C′ Inhibition Is Induced by Human Vaccination (A) Invasion-inhibitory activity of rabbit antibodies specific to merozoite surface antigens in the presence of 50% NS and HIS. Data represent the mean ± range from two independent assays performed in duplicate. (B) Invasion-inhibitory activity of naturally acquired human affinity-purified MSP2 and MSP3 antibodies at 50 μg/ml in the presence of 50% NS or HIS. Data represent the mean ± SEM from four independent assays performed in duplicate. (C) Invasion inhibition with human mAbs (IgG1) to MSP2 in the presence of 50% NS or HIS (mAb concentration 50 μg/ml). Recombinant MSP2 mAb was expressed with the wild-type sequence or with a LALA Fc mutation, which is known to ablate C1q fixation. Two independent assays were performed in duplicate. (D) Invasion-inhibitory activity of purified IgG from malaria-naive adults immunized with recombinant MSP2 antigen was tested in the presence of 50% NS or HIS at a 1/2 dilution of the original concentration. Data represent the mean ± range from two independent assays performed in duplicate. (E) Overall, inhibitory activity by IgG from individuals receiving the MSP2 vaccine was greater when it was tested in the presence of 50% NS than when it was tested in the presence of HIS. See also Figure S5.

To identify merozoite antigens that are targets of Ab-C′ inhibition, we tested rabbit antibodies to several major merozoite surface antigens for inhibition of invasion in the presence of NS or HIS. Antibodies to MSP1-19, MSP1 block 2, and MSP2 substantially inhibited invasion in the presence of NS, but not HIS (Figure 5A). Activity was specific, and no inhibition was seen with antibodies from non-immunized rabbits (data not shown). Antibodies to the MAD20-like MSP1 block 2 alleles used in the parasite line tested (MAD20 and Wellcome alleles) inhibited invasion, whereas antibodies to heterologous K1-like (3D7 or Palo Alto) alleles did not, reflecting the strain specificity of the antibodies. It is notable that some antibodies to MSP2 and MSP1 block 2 enhanced invasion in HIS, as was seen with some antibodies from naturally exposed subjects (Figure 3), whereas they were inhibitory in the presence of NS. With rabbit antibodies to MSP3, MSP4, and AMA1, we observed minimal differences in invasion-inhibitory activity between NS and HIS.

MSP2 and MSP3 are vaccine candidates that have progressed to clinical trials, but development has been hampered by the lack of immunologic correlates of protection, given that these antibodies are relatively non-inhibitory in standard GIAs (McCarthy et al., 2011; Oeuvray et al., 1994). The function of human antibodies to MSP2 and MSP3 was defined with affinity-purified antigen-specific human antibodies in invasion-inhibition assays (Figure 5B). In agreement with results from rabbit antibodies, purified human anti-MSP2 antibodies significantly inhibited invasion in NS, but not in HIS. In contrast, MSP3 antibodies showed a limited amount of direct inhibitory activity and no enhancement by complement.

To further confirm the role of complement fixation in mediating invasion inhibition and the significance of MSP2 as a target, we tested a human MSP2-specific monoclonal antibody (mAb) with and without a specific change (L234A or L235A [LALA]) in the amino acid sequence of the Fc region (Stubbs et al., 2011); this change ablates binding to C1q and complement activation but leaves binding to the antigen unaffected (Hessell et al., 2007). Significantly greater invasion inhibition in NS than in HIS was only seen with wild-type MSP2 mAb and not with the modified mAb. Further, invasion inhibition was greater in NS with the wild-type than in NS with the altered mAb. These results further confirm the importance of C1q fixation in Ab-C′ inhibition and MSP2 as a target.

### Ab-C′ Inhibitory Antibodies Can Be Induced by Human Immunization

We examined whether Ab-C′ inhibitory activity could be induced by immunization of malaria-naive individuals with recombinant merozoite surface proteins. We studied samples from the recent phase 1 clinical trial of the MSP2-C1 vaccine (McCarthy et al., 2011). IgG from ten individuals with high C1q-fixation activity (as defined by ELISA) were tested for invasion-inhibitory activity in NS and HIS (Figures 5D and 5E). IgG from these individuals lacked inhibitory activity in standard GIAs despite high antibody reactivity by ELISA (McCarthy et al., 2011). Notably, eight of ten individual IgG samples showed substantial inhibition in NS, but not in HIS, indicating the induction of Ab-C′ inhibition by vaccination. No inhibition was seen in IgG from pre-vaccinated individuals or placebo-vaccinated samples (Figure S5). These data indicates that MSP2 antibodies induced by vaccination are able to inhibit invasion via Ab-C′ inhibition and identify a potential mechanism mediating the protective efficacy of MSP2-based vaccines (Genton et al., 2002).

## Discussion

Although the importance of antibody in immunity to malaria has been established (Cohen et al., 1961), mechanisms mediating protection are poorly understood. Here, we provide evidence that complement plays a key role in antibody-mediated immunity to malaria in humans. Antibodies from malaria-exposed individuals enhanced complement fixation on merozoites and had substantially greater invasion-inhibitory activity in the presence of complement. Ab-C′ inhibition was the predominant mechanism inhibiting invasion, and many antibodies were only inhibitory in the presence of complement factors. Our findings indicate that the mechanism underlying this activity is predominately mediated by C1q fixation. Antibody-complement interactions also led to merozoite lysis. Targets of Ab-C′ include the most abundant merozoite surface antigens, MSP1 and MSP2. Furthermore, we provide epidemiologic evidence of the role of antibody-complement interactions in human immunity by demonstrating that C1q fixation was very strongly associated with protection from clinical malaria and high-density parasitemia in a prospective longitudinal study of children. Finally, we demonstrated that Ab-C′ inhibition can be induced by human immunization, providing an important proof of concept for translation into malaria vaccine development.

Comparisons of Ab-C′ inhibitory activity in serum depleted and reconstituted with C1q or C5 highlight the importance of C1q in mediating inhibition of invasion. Further, C1q alone was able to significantly enhance the inhibitory activity of anti-malarial antibodies in the absence of other complement factors. This was further demonstrated by comparison of wild-type and altered human MSP2 mAbs; Ab-C′ inhibition was only seen with the wild-type and not the altered mAb. C1q-mediated antibody neutralization has been previously reported with influenza (Feng et al., 2002) and West Nile virus (Mehlhop et al., 2009). Enhanced inhibition by C1q might be due to increased steric hindrance by the large (460-kDa) C1q-IgG complex blocking binding of parasite proteins to cellular receptors or through the stabilization of IgG of low avidity. In complement-free systems, some antibodies to MSP1 (Blackman et al., 1994; Dluzewski et al., 2008) and MSP2 (Boyle et al., 2014) can be internalized into the RBC while bound to the merozoite surface without inhibiting invasion. However, in the presence of complement, antibodies to MSP2 and MSP1 effectively inhibit invasion. The C1q-IgG complex might be too large to be internalized during invasion, thereby mediating the inhibitory activity of antibodies that are otherwise not directly inhibitory.

Although C1q-mediated enhancement appears central to invasion-inhibitory activity, complement deposition and lysis of merozoites are likely to have other implications in vivo, including enhancement of phagocytosis and induction of pro-inflammatory cytokines that might further mediate control of parasitemia. Complement activation, particularly as part of the innate immune response, has been implicated in pathogenesis as a result of induction of inflammatory responses (reviewed in Biryukov and Stoute, 2014; Silver et al., 2010). In the absence of anti-malarial antibodies, complement did not inhibit merozoite invasion, despite some deposition of C3b and MAC on the parasite surface and the eventual lysis of merozoites in the absence of malaria-exposed IgG with extended incubations. This is most likely due to the reduced rate and extent of complement deposition in the absence of specific antibody and might also indicate that merozoites could be able to inhibit complement activation, as described for other pathogens (Lambris et al., 2008).

Testing antibodies from various individuals demonstrated that Ab-C′ inhibition was the predominant mechanism for inhibition of invasion; the extent of inhibition and the prevalence of Ab-C′ inhibition were greater than direct antibody inhibition, and Ab-C′ inhibition increased with age, reflective of the known acquisition of immunity. Strong epidemiologic evidence of the importance of complement fixation in antibody-mediated immunity to malaria was established in a longitudinal cohort of children acquiring immunity. High-C1q-fixing antibodies were very strongly associated with protection from clinical malaria. Furthermore, complement fixation was strongly associated with protection from high-density parasitemia, consistent with the proposed role of Ab-C′ inhibition in limiting parasite replication and thereby preventing disease. These findings provide insight into the potential role of complement fixation in protective humoral immunity in humans and contrast with the limited and inconsistent associations reported for antibody activity measured in standard GIAs. The GIA is performed in complement-free conditions and is currently the only widely used functional assay of antibodies to merozoites (Crompton et al., 2010; Dent et al., 2008; John et al., 2004; Marsh et al., 1989; McCallum et al., 2008; Wilson et al., 2011). We propose that the weak and inconsistent correlation between standard GIAs and malaria immunity reflects the central importance of complement factors in mediating antibody activity. Further, Ab-C′ inhibition strongly correlated with reactivity of cytophilic antibodies to merozoites, particularly IgG3. This fits with the properties of IgG3 as the most potent activator of complement and is consistent with findings that ELISA titers of IgG3 to merozoite antigens are associated with protection in human cohort studies (Courtin et al., 2009; Ndungu et al., 2002; Nebie et al., 2008; Richards et al., 2010; Roussilhon et al., 2007; Stanisic et al., 2009). Merozoite proteins MSP1 and MSP2 were identified as important targets of Ab-C′ inhibition. Antibodies to MSP1 block 2 and MSP2 show limited inhibition in standard GIAs (Boyle et al., 2014; Cowan et al., 2011; Flueck et al., 2009; Galamo et al., 2009; McCarthy et al., 2011). The block 2 region of MSP1 is polymorphic and under balancing selection, suggesting immune pressure. Consistent with this, the Ab-C′ inhibitory activity of MSP1 block 2 antibodies was strain specific, supporting the view that polymorphisms have evolved to mediate immune evasion.

Our studies have established a proof of principle that Ab-C′ inhibitory antibodies can be induced by human vaccination with recombinant merozoite surface antigens with the use of samples from a phase 1 trial of the MSP2-C1 vaccine. A vaccine based on MSP2 was previously found to have significant protective efficacy against *P. falciparum* parasitemia in a naturally exposed population in PNG (Genton et al., 2002). However, the mechanism of protective function of MSP2 antibodies has been unclear (McCarthy et al., 2011). Here, we have shown that antibodies to MSP2 interact with complement to inhibit invasion. The identification of Ab-C′ activity as a central protective mechanism of antibodies targeting merozoite antigens might suggest a role for this assay in evaluating candidate vaccines. Unlike the phase 2 vaccine trial of MSP2, a phase 2 trial of a MSP1 42-kDa C-terminal construct (MSP1-42) had no efficacy in a naturally exposed population in Kenya (Ogutu et al., 2009). Vaccine failure might be explained by antigenic diversity, given that only a single allele of the polymorphic MSP1-42 antigen was included, or by the nature, epitope specificity, or concentration of antibodies induced; the vaccine might have failed to induce strong complement-fixing antibodies to effectively inhibit invasion, which could be investigated in future studies.

In conclusion, we have identified Ab-C′ inhibition as a prominent mechanism targeting the merozoite in naturally acquired immunity, and we found that complement-dependent inhibition can be mediated by antibodies induced by human immunization with a recombinant merozoite surface-protein vaccine. Our findings demonstrate that human anti-malarial antibodies have evolved to function in the presence of complement by recruiting complement for functional activity and protective immunity. These insights mark a major change in our understanding of mechanisms of functional immunity and provide tools for evaluating naturally acquired and vaccine-induced immunity. Our findings might have translational implications, indicating that focusing on targets and strategies that induce strong complement-fixing antibodies might be an important step in the development of highly efficacious vaccines.

## Experimental Procedures

Further details can be found in the Supplemental Experimental Procedures.

### Parasite Culture, Synchronization, and Invasion-Inhibition Assays

The *P. falciparum* D10-GFP expression line was cultured as previously described and synchronized with heparin (Boyle et al., 2010a; Wilson et al., 2010). Invasion-inhibition assays were performed as described in Boyle et al. (2010a); merozoites were incubated with uninfected RBCs, normal or heat-inactivated serum (NS or HIS, respectively), and test IgG for 30 min (Figure S1A). Cells were washed and cultured for 40 hr and then analyzed via flow cytometry. NS and HIS was from malaria-naive donors. For heat inactivation, sera were heated at 56°C for 30 min. For assays testing the importance of the alternative pathway, sera were heat inactivated at 50°C for 20 min. For assays testing the importance of C1q and C5 for Ab-C′ activity, human serum depleted of C1q or C5 and purified human C1q and C5 (Calbiochem, Merck) were used at 25% concentration. GIAs were performed as described in Persson et al. (2006).

### Human Subjects and Samples

Ethical approval for the use of human serum and plasma samples in these studies was obtained from the Alfred Human Research and Ethics Committee for the Burnet Institute, from the Kenya Medical Research Institute, from the Medical Research Advisory Committee of Papua New Guinea, and from the Human Research and Ethics Committee of the Queensland Institute of Medical Research. Written informed consent was obtained from all participants or, in the case of children, from their parents or guardians. Serum pools from malaria-exposed donors were made from serum samples from Kenya (Ngerenya, Kilifi District) and PNG (Madang District). Unexposed serum pools were from Australian donors residing in Melbourne (Australia Red Cross Blood Bank). IgG from serum pools was purified with Melon Gel according to the manufacturer’s (Thermo Scientific) instructions. Purified IgG was concentrated in 10-kDa MWCO (molecular weight cutoff) spin-purification tubes (Amicon) and buffer exchanged with PBS. For the longitudinal study of PNG children, plasma samples were obtained at enrollment from a prospective treatment-reinfection cohort of 206 children aged 5–14 years (median = 9.3) in Madang, PNG (Michon et al., 2007). Children were actively reviewed every 2 weeks for symptomatic illness and parasitemia, and by passive case detection, over a period of 6 months. A clinical episode of *P. falciparum* malaria was defined as fever and *P. falciparum* parasitemia >5,000 parasites/μl. Serum samples were used from a phase 1 MSP2-C1 vaccine trial in which participants were immunized with both 3D7 and FC27 MSP2 isoforms formulated with Montanide ISA 720 (McCarthy et al., 2011) (sponsored by PATH Malaria Vaccine Initiative; Trial Registration, AWZCTR 12607000552482).

### Rabbit and Human Antibodies to Specific Merozoite Antigens

Rabbit sera were raised against recombinant proteins corresponding to MSP1-19, MSP1 block 2, MSP4, MSP2, the MSP3 C-terminal region, and AMA1 (3D7 and 7G8 alleles) as described in Boyle et al. (2014) and Drew et al. (2012). Human antibodies to MSP2 (FC27) and MSP3 (K1) were purified from PNG and Kenyan serum donors via column chromatography according to established methods (Reiling et al., 2012). A human mAb to MSP2 was previously isolated from a malaria-exposed donor and expressed as recombinant IgG1 with the wild-type sequence or with a Fc-LALA mutation (Stubbs et al., 2011).

### Complement Deposition Assays via Immunoblot, ELISA, and Microscopy

Merozoites were incubated with 25% NS and test IgG or serum samples for 1, 5, 10, 15, or 30 min at 37°C. Merozoites were washed and solubilized for immunoblot analysis. C1q and C3 were detected with anti-C1q (Goat polyclonal, Calbiochem, Merck) and anti-C3 (HRP-conjugated goat polyclonal, MP Biomedicals), respectively.

For ELISA, plates were coated with purified merozoites at 5 × 10^6^ merozoites/well. Plates were blocked and then incubated with sera samples (1/250), and then recombinant C1q (10 μg/ml) and C1q were detected with goat anti-C1q antibodies and anti-goat-HRP. C1q and C3 deposition was also detected with C5-depleted serum as a complement source. For ELISA analysis of MAC deposition, isolated merozoites were incubated with 25% NS serum and IgG from PNG or Australian (Melbourne) serum pools for 10 min at 37°C and then washed and coated into Nunc 96-well plates. Plates were blocked, and the presence of MAC was detected with anti-C5–C9 antibodies (rabbit) followed by anti-rabbit-HRP.

For immuno-electron microscopy, merozoites were incubated with NS or HIS with PNG IgG for 10 min. Merozoites were washed and fixed in 1% glutaraldehyde and then processed and imaged as described in Boyle et al. (2010a). For IF microscopy, merozoites were incubated with 25% NS serum and IgG from PNG or Melbourne donors for 10 min (37°C). Merozoites were washed and dried on slides, fixed with methanol, and blocked, and then MAC was detected with anti-C5–C9 antibodies (rabbit) and anti-rabbit-Alexa 488 antibodies. Images were obtained as described in Reiling et al. (2012).

### Assays of Merozoite Lysis

Merozoites were incubated with 5% PNG or Melbourne IgG and 20% NS, HIS, or C1q-depleted serum for 10 min (37°C); for assays to assess the rate of merozoite lysis, an aliquot of sample was taken every minute for analysis by flow cytometry. Samples were diluted 1/100 in 200 μl cold PBS and 1% newborn calf serum, and the density of merozoites was counted with CountBright counting beads via flow cytometry.

### ELISA to Intact Merozoites

ELISAs were performed according to standard methods (Stanisic et al., 2009). Purified merozoites were coated in PBS and placed on microtiter plates. Merozoites were blocked and subsequently incubated with Kenya serum samples diluted at 1/250 and then sheep anti-human IgG HRP diluted at 1/2,500.

### Data Analysis

Differences in invasion-inhibitory IgG activity between NS and HIS and between C1q-depleted and -reconstituted serum were calculated with paired t tests in Stata/SE 11.2. Associations between antibody reactivity to intact merozoites by ELISA and functional activity in assays of Ab-C′ inhibition, direct inhibition, and growth inhibition were assessed with Spearman’s correlations calculated in Prism.

Analysis of the cohort study was performed with Stata/SE 12.0. Differences in C1q deposition between groups were assessed by chi-square tests (for categorical variables) or Wilcoxon rank-sum tests (for continuous variables). For assessment of associations between C1q deposition and protection, subjects were stratified into tertiles according to low (including those classified as “negative”), medium, or high deposition of C1q, as determined by optical-density values for each sample. Groups were compared for risk of clinical malaria (fever and >5,000 parasites/μl) or high-density parasitemia (>5,000 parasites/μl) with the Cox proportional-hazards model (Reiling et al., 2010; Richards et al., 2010). Survival analysis included first episodes only. Age and location of residence were previously identified as potential confounders from a range of factors (Stanisic et al., 2009).

## Author Contributions

M.J.B., L.R., G.F., and J.G.B. planned the study and interpreted results with input from all authors. M.J.B., L.R., Y.S.C., G.F., H.A.J., and C.L. performed experiments. F.H.O., K.K.A.T., D.J.C., I.M., J.S.M., K.M., J.S., and R.F.A. provided key reagents. M.J.B., L.R., G.F., and J.G.B. wrote the manuscript with input from all authors.

## Figures and Tables

**Table 1:** Correlation between Invasion-Inhibition Assays and GIAs and Antibodies to the Merozoite Surface

	Antibody Prevalence^b^	Functional Activity^a^					
		IIA-Ab-C′		IIA-Direct		GIA	
		r^c^	p	r^c^	p	r^c^	p
Total IgG	82%	0.57	<0.001	0.18	0.31	0.41	0.02
IgG1	85%	0.56	<0.001	0.13	0.49	0.32	0.07
IgG2	55%	0.46	0.01	0.37	0.04	0.52	0.001
IgG3	52%	0.73	<0.0001	0.16	0.37	0.48	0.005
IgG4	3%	0.49	0.07	0.07	0.70	0.3	0.09

a Functional activity of individuals was measured in invasion-inhibition assays with NS (IIA-Ab-C′) and HIS (IIA-direct) and in a standard GIA that measures growth-inhibitory activity over two invasion cycles in complement-free conditions.

b Thirty-three Kenyan serum samples were tested for total IgG, IgG1, IgG2, IgG3, and IgG4 on the merozoite surface by ELISA. Positive responses were defined as greater than the mean optical-density values of Australian (Melbourne) controls plus 3 SDs.

c Spearman correlation coefficients.

**Table 2:** Association between Antibody-Mediated C1q Deposition on the Merozoite Surface and Protection from Clinical Malaria and High-Density Parasitemia

		uHR (95% CI)	p	aHR (95% CI)	p
Clinical malaria	LvM	0.56 (0.34–0.94)	0.028	0.64 (0.38–1.09)	0.1
	LvH	0.12 (0.05–0.28)	<0.0001	0.15 (0.06–0.35)	<0.0001
High-density parasitemia	LvM	0.80 (0.50–1.30)	0.37	0.94 (0.58–1.53)	0.804
	LvH	0.26 (0.13–0.49)	<0.0001	0.35 (0.18–0.70)	0.003

The cohort was stratified into three equal groups according to low, medium, or high C1q reactivity (see also Table S1). Unadjusted hazard ratios (uHRs) and HRs adjusted for age and location of residence (aHRs) were calculated to compare low-versus-medium (LvM) or low-versus-high (LvH) groups for the risk of symptomatic malaria or high-density parasitemia (>5,000 parasites/μl) over the time period of 6 months. Calculations were based on the first episode only.

## Supplemental Information

Document S1. Figures S1–S5, Table S1, and Supplemental Experimental Procedures

Document S2. Article plus Supplemental Information
