# Supplementary material for: Human Antibodies Fix Complement to Inhibit Plasmodium falciparum Invasion of Erythrocytes and Are Associated with Protection against Malaria
Source: Immunity. 2015 Mar 17;42(3):580–90. doi: 10.1016/j.immuni.2015.02.012 (PMC4372259; doi:10.1016/j.immuni.2015.02.012)
Supplement: Document S1. Figures S1–S5, Table S1, and Supplemental Experimental Procedures [file mmc1.pdf]

Immunity

Supplemental Information

Human Antibodies Fix Complement to Inhibit *Plasmodium falciparum* Invasion of Erythrocytes and Are Associated with Protection against Malaria

Michelle J. Boyle, Linda Reiling, Gaoqian Feng, Christine Langer, Faith H. Osier, Harvey Aspeling-Jones, Yik Sheng Cheng, Janine Stubbs, Kevin K.A. Tetteh, David J. Conway, James S. McCarthy, Ivo Muller, Kevin Marsh, Robin F. Anders, and James G. Beeson

Supplementary Data

Supplementary Figure 1:

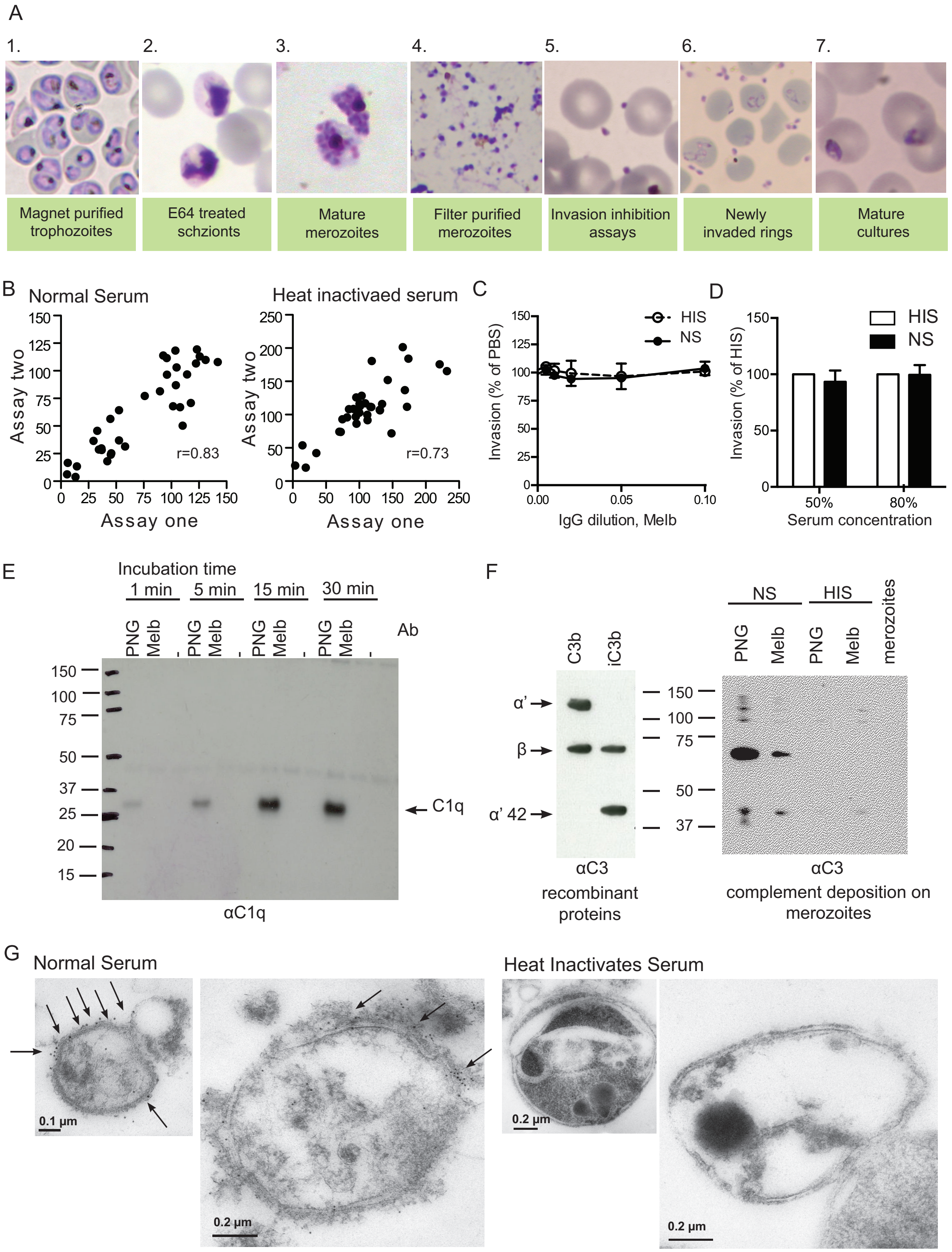

Supplementary Figure S1: Invasion inhibition assays and complement deposition on merozoites (see also Figure 1).

A. Procedure for isolation of merozoites and invasion inhibition assays – refer to Supplementary Experimental Procedures; 1) synchronized mature parasites are purified via MACs separation. 2) Cultures are matured to segmented schizonts and treated with E64. 3) Mature merozoites are formed within E64 treated schizonts. 4) Merozoites are isolated via membrane filtration. 5) Isolated merozoites are incubated with uninfected RBCs, normal or heat-inactivated serum and purified IgG for 30 minutes in invasion inhibition assays. 6) Invaded rings are washed and returned to standard culture conditions. 7) Parasites are cultured in standard conditions until mature and enumerated via flow-cytometry. Figure is modified from (Boyle et al., 2013). **B.** Invasion inhibition assays with Normal Serum (NS) and Heat-inactivated serum (HIS) are reproducible; purified IgG from 33 Kenyan donors were tested with 50% NS and 50% HIS in invasion inhibition assays on two separate experimental days. Assays were reproducible; Spearman's rho was 0.83 for inhibition with NS (95% CI; 0.69 0.92, $p<0.001$) and $r=0.73$ for inhibition in HIS (95% CI; 0.51 0.86, $p<0.001$). **C.** IgG from unexposed donors has no inhibitory activity with NS or HIS; Purified merozoites were allowed to invade RBCs in the presence of 50% NS or HIS with IgG from serum pools from residents in Melbourne, Australia (data are mean ± range of two assays in duplicate). **D.** Complement alone does not inhibit invasion; Purified merozoites were allowed to invade RBCs in the presence of normal serum (NS) or heat-inactivated serum (HIS) at 50% and 80% final serum concentrations (data is mean ± SEM. of four assays in duplicate). **E.** Specificity of C1q antibodies used in complement deposition assays; Entire western blot of C1q deposition on merozoites from Figure 1b, indicating that anti-C1q antibody is specific, and reacts with a single protein of approximately 30kDa, consistent with C1q. **F.** Specificity of C3 antibodies and reactivity to C3b and iC3b; Right panel shows C3 antibody reactivity with recombinant C3b and iC3b, with arrows indicating α and β chains. Left panel shows complement deposition on merozoites incubated with IgG from PNG or Melbourne donors, incubated with normal serum (NS) or heat-inactivated serum (HIS). **G.** Deposition of C3b on merozoites by immune-electron microscopy; Two example images of C3b deposition on the merozoite surface with merozoites incubated with PNG IgG and normal serum (left panels), and the lack of C3b deposition on merozoites incubated with PNG IgG and heat-inactivated serum (right panels).

Supplementary Figure 2:

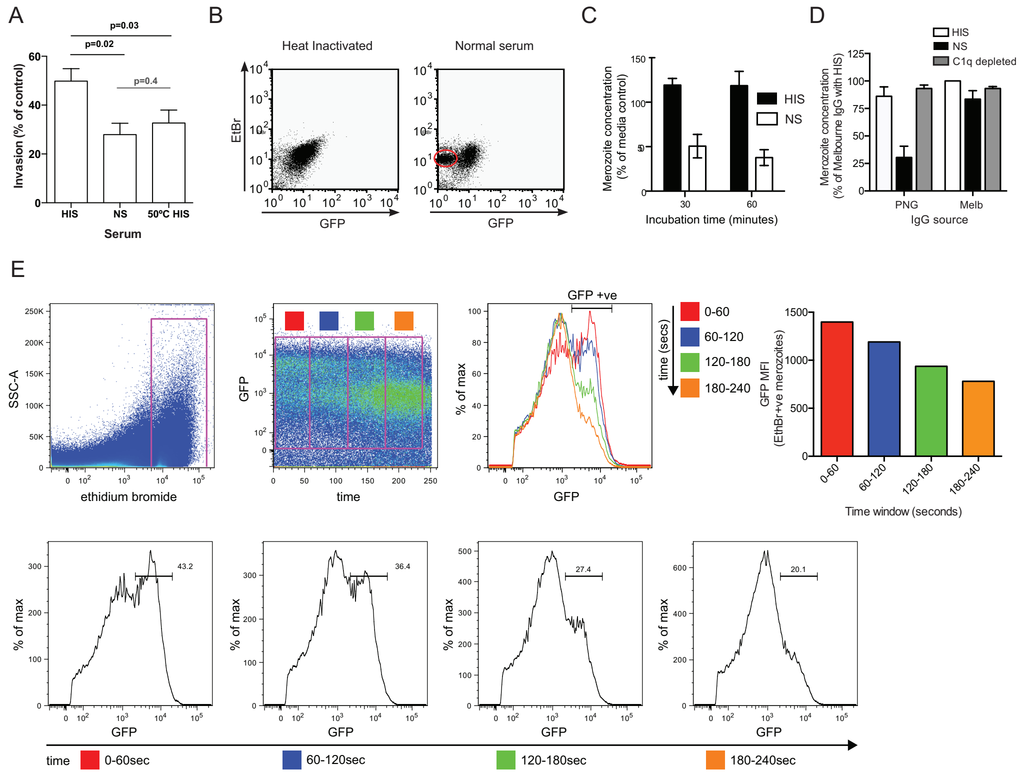

Supplementary Figure S2: Importance of different complement components in mediating inhibition (see also Figure 2).

A. Alternative complement pathway is less important than classical complement pathway; Invasion inhibition activity of PNG IgG was tested in 25% serum that heat-inactivated serum at 56°C for 30 minutes (HIS), normal serum (NS) and serum heat-treated at 50°C for 20 minute (50°C HIS). Heat-inactivation at 56°C for 30 minutes disrupts all complement activation cascades, while heat-treatment at 50°C for 20 minutes disrupts only the alternative complement cascade by inactivation of Factor B, while leaving the classical complement cascade intact. The 50°C treatment of serum had no significant effect on the invasion-enhancing activity of serum; invasion-inhibition by PNG IgG was significantly greater in the presence of NS and 50°C-treated serum, compared to HIS. **B.** Analysis of merozoite lysis; Merozoites were incubated with HIS or NS and lysis monitored via flow cytometry by assessing loss of cytosolyic GFP expression by *P. falciparum* merozoites. In NS, merozoites lost GFP expression as indicated by the red circle. Intact merozoites were counted with count bright counting beads by gating on EtBr positive and GFP positive merozoites. **C.** Merozoite lysis occurs in the absence of IgG in extended incubations; Merozoites were incubated for 30 or 60 minutes with NS and HIS in the absence of IgG. Merozoite lysis was evident after prolonged incubation, indicating that over extended time periods lysis of merozoites did

occur, likely mediated by the activation of alternative complement pathways on the merozoite surface. **D.** Rapid lysis of merozoites requires C1q; Merozoites were incubated for 10 minutes with NS or HIS or C1q-depleted serum together with IgG from PNG or Melbourne, Australian donors. Merozoite lysis after ten minutes of incubation was only observed in NS with IgG from PNG donors, indicating that lysis was dependent on the activation of the classical complement cascade by merozoite specific IgG. **E.** Gating strategies for assessing the rate of merozoite lysis; To assess the rate of lysis of merozoites incubated with NS and malaria-specific IgG, merozoites were incubated at 37°C with PNG IgG and NS and monitored via flow-cytometry during lysis. Merozoites were gated on EtBr positive cells, and then GFP MFI was monitored. GFP positive cells were rapidly lost within 1-4 minutes of incubation, as indicated by the loss of positive GPF cells and overall decreased MFI of merozoites.

Supplementary Figure 3:

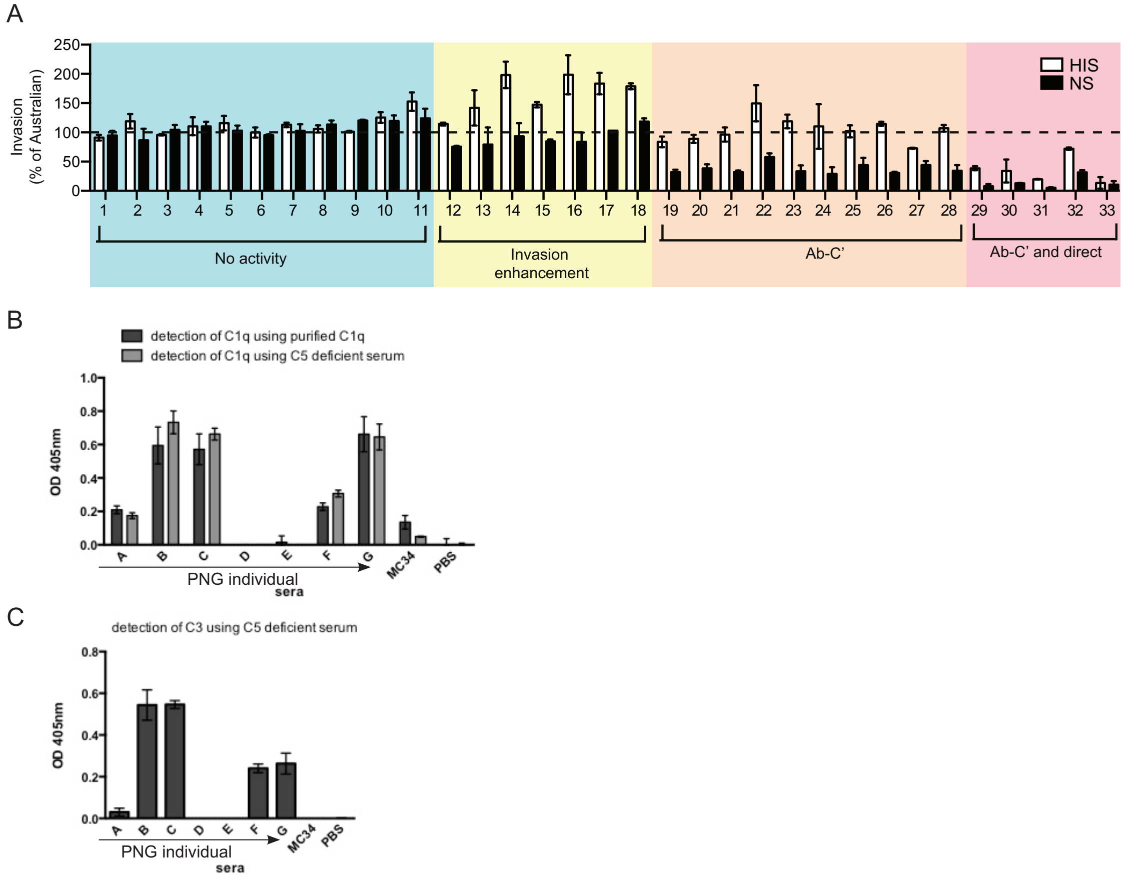

Supplementary Figure S3: Ab-C’ inhibition in individuals and fixation of complement components on merozoites (see also Figure 3).

A. <u>Ab-C’ is the predominant mechanism of invasion-inhibition in naturally acquired antibodies</u>; Invasion inhibition activity profiles of purified IgG from all 33 Kenyan individuals in the presence of NS and HIS; no inhibition activity (blue), invasion enhancement activity (in HIS and not NS) (yellow), Ab-C’ inhibition (orange) and Ab-C’ and direct inhibitory activity (red) (data are mean ± range of two assays in duplicate). Sample numbers do not necessarily correspond with those represented in Figure 3b. **B.** <u>Quantification of C1q deposition with PNG individuals using C1q and C5-deficient serum on merozoite;</u> C1q-fixation on the merozoite surface by IgG from PNG individuals (A-G), IgG from Melbourne donors (MC34) or control (PBS). Either purified C1q or C5-deficient serum was used as the source of C1q in the assays. **C.** <u>Quantification of C3b deposition with PNG individuals using C5-deficient serum on merozoites;</u> C3b-fixation on the merozoite surface by IgG from PNG individuals (A-G), IgG from Melbourne donors (MC34) or control (PBS). C5-deficient serum was used as the source of complement in the assays.

Supplementary Figure 4:

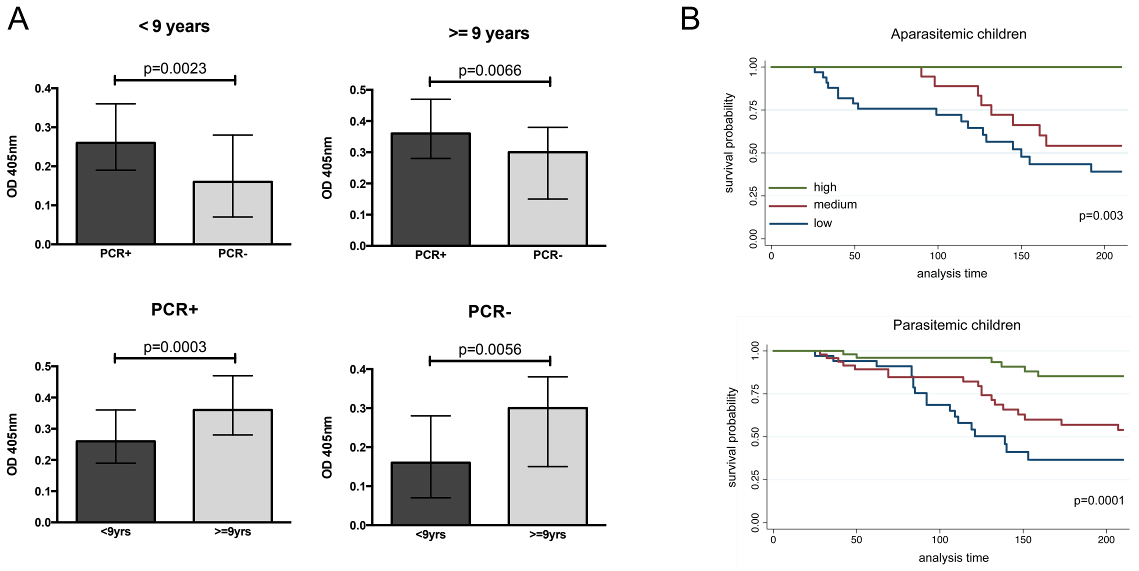

Supplementary Figure S4: Analysis of complement fixation in the children's cohort stratified by age and parasite status (see also Figure 4).

A. C1q fixation by children; C1q fixation (median OD) by children's samples was stratified by parasitemia status at enrolment (determined by PCR) and age. C1q fixation was significantly higher in the presence of parasitemia among older and younger children. Older children had greater C1q fixation than younger children regardless of whether there was active parasitemia. **B.** Association between complement fixing antibodies and clinical malaria; Kaplan Meier survival curves for time to first clinical episode of malaria with children stratified into the groups of high, medium and low C1q fixation. Survival curves are shown for children stratified by parasitemia status at time of enrolment; all children were subsequently treated to clear parasitemia prior to follow-up (p=0.003 for aparasitemic children, and p=0.0001 for parasite positive children, comparing high medium and low responder groups.)

Supplementary Figure 5:

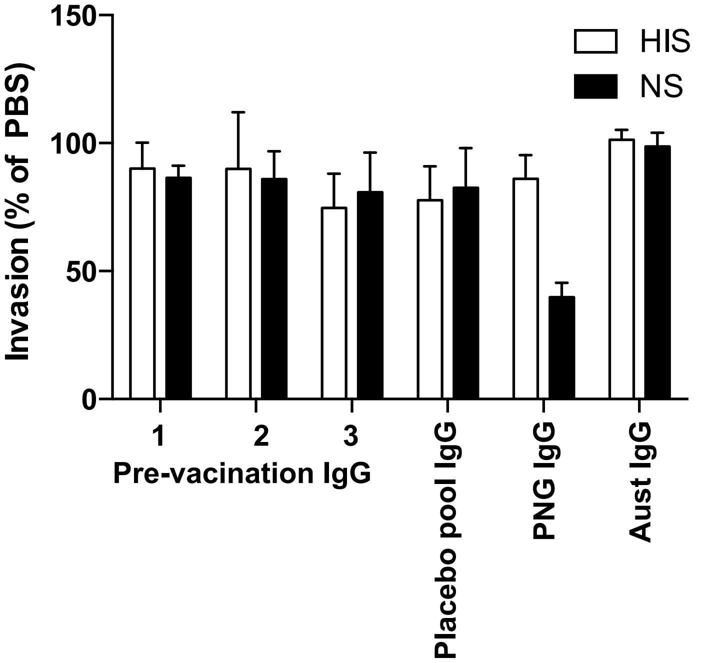

Supplementary Figure S5: Pre-vaccination samples and placebo vaccine serum pools in invasion inhibition assays (see also Figure 5).

Purified IgG from individuals prior to vaccination and from day 120 post vaccination placebo sample pools were tested in invasion inhibition assays with normal serum (NS) and heat-inactivated serum (HIS). Data represent two assays in duplicate and normalized to invasion with Australian IgG. PNG IgG and Australian IgG were used as positive and negative controls respectively.

Supplementary Table S1: Prevalence and levels of antibody-mediated C1q deposition on the merozoite surface in the cohort of children (related to table Table 2).

		Age [4]			Enrolment P. falciparum parasitemic status [5]		
	**All n =200** [7]	**≤ 9yrs n=90**	**> 9yrs n=110**	**p**	**PCR- n=65**	**PCR+ n=135**	**p**
**Seropositive**[1] **%**[2]	198 99%	88 97.8%	110 100%	0.116	64 98.5%	134 99.3%	0.5
**Median OD [IQR]** [3]	0.30 [0.20-0.41]	0.24 [0.14-0.33]	0.35 [0.26-0.43]	**<0.0001**	0.23 [0.08-0.34]	0.34 [0.23-0.43]	**<0.0001**

[1] Number of individuals from the cohort with C1q deposition on merozoites' surface

[2] %: percent of individuals from the cohort with C1q deposition on merozoites' surface

[3] [IQR]: inter-quartile range

[4] Age: the cohort was stratified by age into two groups: children 9 years of age an younger (≤) or older than 9 years of age

[5] Enrolment *P. falciparum* parasitemic status: PCR- indicates aparasitemic status at enrolment, PCR+ indicates parasitemic status at enrolment, both as determined by PCR

[7] Number of samples is 200 due to insufficient sample volume or excluded values where the discrepancy between duplicates was >25%.

Supplementary Experimental Procedures

Parasite culture and synchronization

The *P. falciparum* D10-GFP expression line (Wilson et al., 2010) was cultured as described (Persson et al., 2006) in culture medium of RPMI-HEPES (pH 7.4) supplemented with 50 μg/ml hypoxanthine, 20 μg/ml gentamicin, 25 mM sodium bicarbonate and 10% (volume/volume) pooled human serum from unexposed Melbourne, Australia blood donors (supplied by the Australian Red Cross Blood service). RBCs from group O+ blood donors (supplied by the Australian Red Cross Blood service) were used to culture parasites. Typically cultures were maintained at 3% hematocrit, with a parasitemia of 1-5%. Cultures were maintained in 1% O_2, 4% CO_2, 95% N_2 and incubated at 37°C. For monitoring of cultures, blood slides were stained with 10% Giemsa (Merck).

Parasites were synchronized using 5% D-sorbitol-treatment; cultures were incubated for 5 minutes with 5% D-sorbitol, pelleted and washed one time with culture media before returning to culture. Cultures were further synchronized using the invasion inhibitory properties of heparin (Boyle et al., 2010a; Wilson et al., 2010). Parasites were cultured in the presence of 30IU (approximately 230 μg/ml) of medical grade heparin (Porcine mucous, Pfizer) until the majority of parasites were at the schizont stage. Under heparin inhibition, invasion of RBCs by merozoites rupturing from schizonts was inhibited. Heparin was then removed from cultures by centrifugation of the culture and re-suspension of cells in fresh culture medium for 4-6 hours allowing merozoite invasion and the development of ring stages in RBCs.

Invasion inhibition assays

Invasion inhibition assays were performed with isolated viable merozoites as described (Boyle et al., 2013; 2010b) (Figure S1). A detailed method for the isolation for viable merozoites and invasion inhibition assay can be found at Methods in Malaria Research, http://www.mr4.org/Publications/MethodsinMalariaResearch.aspx. Late stage trophozoites were magnet purified on MACs purification column to remove uninfected RBCs. Following purification, parasites were incubated in standard culture media with

10μM of trans –Epoxysuccinyl-L-leucylamido(4-guanidino)butane (E64) cysteine protease inhibitor for 6-8 hours. This cysteine protease inhibitor allows for the maturation of merozoites but prevents schizont rupture (Blackman, 2008; Boyle et al., 2010b; Glushakova et al., 2009). Following incubation, mature merozoites were formed and isolated via membrane filtration in a 1.2 μm filter; cultures were washed once to remove E64 and resuspended in serum free culture media in a volume required for invasion inhibition assays (for example, if 1ml of isolated merozoites was required, E64 treated cultures were resuspended in 1.5ml of media prior to filtration). Isolated merozoites were then added to uninfected RBCs (final concentration 0.5% haematocrit), test IgG (typically at 1/10 dilution or as indicated in results) and normal serum (NS, complement active – concentrations indicated in results) or heat inactivated serum (HIS, complement in-active – concentrations indicated in results) in a 96 well plate in 50μl suspensions. Cultures were incubated for 10 minutes with agitation on a plate shaker at 500rpm and then transferred to a gassed chamber (standard culture gas conditions) for a further 20 minutes of incubations, allowing for a total invasion time of 30 minute. A 30 minute incubation period was chosen as prior published data indicates that merozoite invasion occurs rapidly, with 80% of invasion occurs within 10 minutes of merozoite contact with RBCs. However, a further 20% of invasion occurs after 10minutes (Boyle et al., 2010b), and it is possible that *in vivo* sequestration of parasites would require free merozoites to make their way to areas of the blood stream where RBCs are available (Boyle et al., 2013). Cultures were then washed twice with incomplete culture media (lacking human serum) and then once with standard culture media to remove IgG and NS and HIS and a proportion of uninvaded merozoites. Cultures were then returned to culture in standard culture media for invaded parasite to mature. Cultures were maintained for 40 hours and then analysed by flow cytometry as described (Boyle et al., 2010b; Wilson et al., 2010). Analysis was performed at 40 hours post invasion due to ease of gating. Invasion assays were reproducible, as measured by Spearman's correlations of two independent assays; for inhibition in NS, correlation coefficient was 0.83 (95% CI; 0.69 0.92, p<0.001) and for inhibition in HIS r=0.73 (95% CI; 0.51 0.86, p<0.001) (Figure S2).

Complement-active serum as the source for invasion inhibition assays (normal serum; NS) was collected from malaria-naïve (Australian) donors; blood was collected

without anti-coagulants and allowed to clot for 1-2 hours at room temperature. RBCs were pelleted at 2800 rpm for 10 minutes and serum aliquoted and stored at -80°C. Serum was thawed once only for use in assays. For heat-inactivation, serum was thawed and heated at 56°C for 30 minutes, to generate heat-inactivated serum (HIS) lacking complement activity. To test for the importance of alternative pathway amplification in Ab-C' activity, serum was heat-inactivated at 50°C for 20 minutes, which disrupts Factor B and the alternative pathway, but does not affect classical pathway activation. For assays testing the importance of C1q and C5 for Ab-C' activity, human serum depleted of complement factors C1q or C5 and purified human C1q and C5 were purchased from Calbiochem, Merck. Due to reagent limitations, a final serum concentration of 25% was used. Ab-C' inhibition activity of PNG IgG at 1:20 dilution was comparable in NS at 25% or 50% concentration.

Growth inhibition assays

Growth inhibition assays were performed as described previously (McCallum et al., 2008; Persson et al., 2006; Wilson et al., 2010). Detailed methods for growth inhibition assays can be found at http://www.mr4.org/Publications/MethodsinMalariaResearch.aspx. Duplicated suspensions of sorbitol synchronized parasites at 0.2% parasitemia, 1% hematocrite were incubated with 10% plasma collected in XYZ tubes from donors in 96-well sterile U-bottom plates (Falcon). Plasma was dialyzed as described (Persson et al., 2006), via 50kDa cut-off Tube-O-Dialyzer in PBS for 2 hours at 4°C. Following dialysis, samples were concentrated in 100kDa cut-off Nanosep spin tubes and restored to original volumes with PBS. Plates were incubated as for parasite culture for 72 hours. At 48 hour time point 5ul of fresh media was added to supplement cultures. After 72 hours, parasites were stained with ethidium bromide and analysed via flow cytometry as described for invasion inhibition assays. The use of flow cytometry for analysis of growth inhibitory assays has been shown to highly replicable (Persson et al., 2006; Wilson et al., 2010).

Human subjects and samples

Ethical approval for the use of human serum and plasma samples in these studies was obtained from the Alfred Human Research and Ethics Committee (for the Burnet Institute), Kenya Medical Research Institute, Medical Research Advisory Committee of Papua New Guinea, and the Human Research and Ethics Committee of the Queensland Institute of Medical Research. Written informed consent was obtained from all participants, or their parents/guardians (in the case of children).

Serum pools from malaria-exposed adults were made from individual serum samples from two geographically distinct regions; Ngerenya pool from Kilifi District, Kenya, and PNG pool from Madang district, Papua New Guinea. Ngerenya samples were collected in a cross sectional survey conducted in 1998 in the Kilifi district in an area of low transmission (Mwangi et al., 2005). For the Ngerenya pool, 33 individual serums samples (age 1 – 68 years) were screened for reactivity to merozoite antigens by ELISA, and the pool made from 27 positive individuals. PNG serum was pooled from 26 individuals (men and women) taken during a cross sectional study performed in Modilon Hospital, Madang and Yagaum Health Centre PNG in 2001/2002 (Beeson et al., 2007). Unexposed serum pools were from Melbourne, Australia donors from Red Cross Blood bank supplies. IgG from serum pools was Melon Gel purified (Thermo Scientific), as per manufacturer's instructions. 500 μl of Melon Gel purification support matrix was equilibrated to room temperature and washed two times with purification buffer though spin columns. Serum was diluted 1:10 with purification buffer. Diluted serum was incubated for five minutes on purification support matrix and the unbound (antibody) fraction was collected in flow through. Diluted purified IgG was concentrated in 10 kDa MWC spin purification tubes (Amicon) and purification buffer was exchanged with PBS to a concentration of < 0.8%. The concentrations of IgG purified from the malaria-exposed and malaria-naïve serum were comparable (data not shown).

Serum samples were used from a Phase 1 vaccine trial of MSP2-C1 where vaccinees were immunised with both 3D7 and FC27 isoforms of MSP2 formulated with Montanide® ISA 720 as described (McCarthy et al., 2011) (sponsored by PATH Malaria Vaccine Initiative; Trial Registration, Australian New Zealand Clinical Trials Registry 12607000552482) . Vaccinated adults were resident of Brisbane, Australia, not from a

malaria endemic country, and had not travelled to a malaria endemic country in the two years prior to vaccination.

For the longitudinal study of Papua New Guinean children, plasma samples were obtained at enrolment from a prospective treatment-reinfection cohort of 206 children aged 5-14 years (median=9.3) in Madang, PNG (Michon et al., 2007). At enrolment the prevalence of was 67.5% (n= 139) by PCR and 40.3% (n= 83) by light microscopy (the geometric mean parasite density was 361 parasites/ml (95% CI, 240–544). After enrolment, all children received 7 days of artesunate orally to clear parasitemia; treatment failures were differentiated from re-infection by genotyping of msp2. Children were actively reviewed every 2 weeks for symptomatic illness and parasitemia by PCR and microscopy, and by passive case detection, over a period of 6 months. A clinical episode of *P. falciparum* malaria was defined as fever and *P. falciparum* parasitemia >5000/ parasites/µl. At enrolment, 21.8% of children had mixed *P. falciparum* and *P. vivax* infections, and 82% of children had PCR-detectable *P. vivax* blood-stage infection during the course of follow-up (Michon et al., 2007). In data analysis of the relationship between antibodies and *P. falciparum* infection, we included all *P. falciparum* infections. In the analysis of associations between antibodies and risk of malaria, we included *P. falciparum* malaria cases only (during follow-up there were very few malaria episodes due to other *Plasmodium* species). We have previously found no relationship between antibodies to *P. falciparum* and *P. vivax* infection, or between *P. vivax* antibodies and *P. falciparum* infection ((Cole-Tobian et al., 2009); and unpublished observations). Additionally, in a prior study on the Thailand-Myanmar border, we similarly found no clear effect of *P. vivax* infection on *P. falciparum* antibodies (Fowkes et al., 2012).

MSP2 and MSP3 human antibodies

Human antibodies to MSP2 (FC27) from naturally exposed individuals were affinity-purified from a serum pool of PNG residents by column chromatography using the two allelic isoforms of MSP2 both as full length recombinant proteins, namely MSP2(3D7) and MSP2(FC27), using established methods (Reiling et al., 2012). Consequently, MSP2(FC27) purified IgG contained antibodies directed only to the FC27-

allelic specific region of MSP2. The D10-PfPHG parasite strain used in these assays expresses the FC27 allele of MSP2 (confirmed by western blot, data not shown).

Human antibodies against the K1 allelic version of MSP3(Polley et al., 2007) were affinity purified from a 50ml pool of plasma taken from malaria semi-immune adults in Kenya using CNBr-activated SepharoseTM 4B (GE Healthcare), using established methods (Reiling et al., 2012).

Monoclonal human antibodies targeting MSP2, along with Fc-LALA mutants were isolated and developed as describe (Stubbs et al., 2011).

Rabbit antibodies

Rabbit serum were raised as described; to MSP1-19 (Stanisic et al., 2009) (Brendan Crabb and Paul Gilson, Burnet Institute); to MSP1-block 2 (Boyle et al., 2010b); to full length MSP4 (Wang et al., 1999) (Ross Coppel and Brian Cooke, Monash University), AMA1 (3D7 and 7G8 alleles) polyclonal rabbit serum (Drew et al., 2012) (Damien Drew, Burnet Institute and Anthony Hodder, Walter and Eliza Hall Institute). Rabbit polyclonal antibodies to MSP2 were generated by vaccination C-terminally His-tagged recombinant MSP2 formulated in Montanide ISA720 as used in the phase clinical trail of MSP2-C1 (McCarthy et al., 2011); Rabbit polyclonal serum was raised to the MSP3 C-terminal MBP-tagged conserved region (Polley et al., 2007).

Complement deposition assays on whole merozoites for western blot and microscopy

To test for the deposition of complement on the merozoite surface, isolated merozoites were incubated with 25% NS with IgG from exposed (PNG), or unexposed (Australian) pools, Ngerenya individuals or PBS for 1, 5, 10, 15 or 30 minutes, agitated at 37°C. After incubation, merozoites were pelleted at 3000g for 5 minutes, washed twice with cold PBS containing complete protease inhibitors and then solubilised in reducing sample buffer and processed for western blot. Deposition of C1q and C3 was assessed using antibodies to C1q (Goat polyclonal, Calbiochem, Merck) and C3 (HRP conjugated goat polyclonal, MP Biomedicals). The merozoite surface protein, MSP1-42 fragment was used as a loading control and was detected with polyclonal rabbit serum to MSP1-19.

For immune-electron microscopy isolated merozoites were incubated with normal and heat inactivated serum with PNG IgG for ten minutes. Merozoites were washed twice in PBS containing complete protease inhibitor and fixed in 1% glutaraldehyde in RPMI-HEPES on ice for 30 min. Samples were pelleted in low-melt agarose before being transferred into water, dehydrated in ethanol, and embedded in LR White Resin (ProSciTech). Following polymerization by benzoyl peroxide (SPI-Chem), 100 nm sections were prepared by using an Ultracut R ultramicrotome (Leica). Sections were post-stained with 2% aqueous uranyl-acetate, and observed at 120 kV on a CM120 BioTWIN transmission electron microscope (Philips).

To detect membrane attack complex (MAC) deposition on merozoites, isolated merozoites were incubated with 25% NS serum and IgG from PNG or Australian serum pools for 10 minutes at 37°C. Merozoites were washed twice in cold PBS with protease inhibitors and either dried on slides for IF-microscopy. Slides were fixed with cold methanol for 5 minutes, dried, blocked with 3% BSA and MAC detected with anti-C5-9 antibodies (rabbit), and anti-rabbit-Alexa 488 conjugated antibodies. Slides were mounted in VectaSheild (Vector Laboratories) with 0.1ng/ml 4',6-diamidino-2-phenylindole (DAPI) to label the parasite nucleus. Images were obtained using a Plan-Apochromate (100X/1.40) oil immersion phase-contrast lens (Carl Zeiss) on an AxioCam Mrm camera (Carl Zeiss). Images were processed using Photoshop CS4 (Adobe). In cases where brightness and contrast were changes, processing was applied to whole images and controls equally.

Complement deposition on whole merozoites for ELISA

Plates were coated with purified merozoites (Boyle et al., 2010b)at $5x10^6$ merozoites/well, incubated overnight at 4 degree. Plates were blocked for 2 hours at 37 degree with 1% casein, incubated with sera samples at 1/250 dilution in 0.1% casein for 2 hours at room temperature (RT). For detection and quantification of C1q fixation, plates were incubation with recombinant C1q at 10 μg/ml in 0.1% casein for 30 minutes at RT. C1q deposition was detected with goat anti-C1q antibodies, followed by anti-goat-HRP, each incubated for 1 hour at RT, diluted 1/500 in 0.1% casein. For detection of C1q and C3 fixation using serum as a complement source, plates were incubated with 20% C5-

deficient serum to prevent lysis of merozoites. C3 was detected with anti-C3-HRP (conjugated goat polyclonal, MP Biomedicals), followed by anti-goat-HRP, each incubated for 1 hour at RT, diluted 1/500 in 0.1% casein. To detect membrane attack complex (MAC) deposition on merozoites, isolated merozoites were incubated with 25% NS serum and IgG from PNG or Australian serum pools for 10 minutes at 37°C. Merozoites were washed twice in cold PBS with protease inhibitors and coated into Nunc plates for ELISA. Merozoites were coated overnight at 4°C in PBS containing protease inhibitors. Wells were then blocked with 10% milk in PBS, and MAC deposition detected with anti-C5-9 antibodies.

Enzymatic activity was detected using ABTS liquid substrate with reactions being stopped after 30 minutes to 1 hour with 1% SDS. Each sample was run in duplicate, and samples with a discrepancy of >25% between duplicates were excluded from the analysis. Variations between plates were accounted for by standardizing using positive controls on each plate. Unexposed controls from Melbourne Australia donors were used as negative controls. For each assay we include control wells, which contain no merozoites. These blank wells give an OD value in the range of 0.1-0.26. This value is regarded as background non-specific signal and deducted from test sample readings, and is well below the signal of the strong positive samples or positive controls.

Merozoite lysis assays

Freshly isolated D10-GPF merozoites were added to a final concentration of 5% hyperimmuned PNG serum (VT pool) and 20% fresh/heat-inactivated serum and incubated at 37 degree for 10 minutes. A pool of sera from malaria-naive Melbourne donors was used instead of PNG serum as negative control. Following incubation samples were diluted 1/100 in 200ul cold PBS-1%NCS (new born calf serum) immediately after incubation (quenching complement deposition). The density of merozoites was counted by flow cytometry following the addition of CountBright counting beads. Merozoites were gated as the GFP-positive population and the density was calculated relative to counting beads; merozoite lysis was expressed as the percentage of lysed merozoites compared to the negative control. For assays to assess rate of merozoite lysis, merozoites were incubated as described above and aliquots taken at

one minute intervals for flow cytometry analysis. Data is expressed as the % of maximum lysis that occurs within 10 minutes of incubation.

ELISA to intact merozoites

ELISA were performed using standard methods (Stanisic et al., 2009). Purified merozoites were coated in PBS onto Maxisorb microtitre plates (Nunc) overnight at 4°C. Plates were blocked with 10% milk for 2 hours at 37°C, followed by incubation with Ngerenya serum samples diluted 1:250 in 5% milk at room temperature for two hours. Binding of antibody was detected with polyclonal sheep anti-human IgG HRP 1:2500 5% milk (Chemicon). Binding was detected with ABTS liquid substrate system (Sigma). Reactions were stopped after 20 minutes with 1% sodium dodecyl sulfate (SDS) and optical density measured at 405 nm. Plates were washed five times with PBS following each incubation.

Data analysis

Differences in invasion inhibition activity for IgG in NS compared to HIS and serum C1q depleted and reconstituted serum was calculated with paired t-test in StataSE 11.2. Associations between antibody levels to intact merozoites via ELISA and functional activity in Ab-C', direct inhibition and growth inhibitory assays were assessed with Spearman's correlations calculated in Prism.

Analysis of the cohort study was performed using Stata/SE 12.0 (StataCorp College Station, Texas, USA). Differences in prevalence and levels of C1q deposition between subgroups were assessed by chi-square tests (for categorical variables) or Wilcoxon rank sum tests (for continuous variable). In order to assess associations between C1q deposition and protection, the subjects was stratified into tertiles according to low (including those classified as 'negative'/'no deposition), medium or high deposition of C1q, as determined by OD values for each sample. Groups were compared for the risk of clinical malaria or high-density parasitemia as described previously (Reiling et al., 2010; Richards et al., 2010). Survival analysis included time to first episode only. The cox proportional hazards model was used to calculate hazard ratios for risk of clinical malaria or high-density parasitemia between different tertiles. Age and location of residence were

previously identified as potential confounders from a range of factors (Michon et al., 2007). Although antibodies at baseline were higher in those children with current parasitemia, parasitemia at baseline was not significantly associated with malaria. As such, hazard ratios were only adjusted for by age and location of residence.

Supplementary Reference

Beeson, J.G., Ndungu, F., Persson, K.E.M., Chesson, J.M., Kelly, G.L., Uyoga, S., Hallamore, S.L., Williams, T.N., Reeder, J.C., Brown, G.V., et al. (2007). Antibodies among men and children to placental-binding Plasmodium falciparum-infected erythrocytes that express var2csa. American Journal of Tropical Medicine and Hygiene *77*, 22–28.

Blackman, M.J. (2008). Malarial proteases and host cell egress: an “emerging”cascade. Cell. Microbiol. *10*, 1925–1934.

Boyle, M.J., Wilson, D.W., and Beeson, J.G. (2013). New approaches to studying Plasmodium falciparum merozoite invasion and insights into invasion biology. International Journal for Parasitology *43*, 1–10.

Cole-Tobian, J.L., Michon, P., Biasor, M., Richards, J.S., Beeson, J.G., Mueller, I., and King, C.L. (2009). Strain-specific duffy binding protein antibodies correlate with protection against infection with homologous compared to heterologous plasmodium vivax strains in Papua New Guinean children. Infection and Immunity *77*, 4009–4017.

Fowkes, F.J.I., McGready, R., Cross, N.J., Hommel, M., Simpson, J.A., Elliott, S.R., Richards, J.S., Lackovic, K., Viladpai-Nguen, J., Narum, D., et al. (2012). New insights into acquisition, boosting, and longevity of immunity to malaria in pregnant women. Journal of Infectious Diseases *206*, 1612–1621.

Glushakova, S., Mazar, J., Hohmann-Marriott, M.F., Hama, E., and Zimmerberg, J. (2009). Irreversible effect of cysteine protease inhibitors on the release of malaria parasites from infected erythrocytes. Cell. Microbiol. *11*, 95–105.

Mwangi, T.W., Ross, A., Snow, R.W., and Marsh, K. (2005). Case definitions of clinical malaria under different transmission conditions in Kilifi District, Kenya. J. Infect. Dis. *191*, 1932–1939.

Polley, S.D., Tetteh, K.K.A., Lloyd, J.M., Akpogheneta, O.J., Greenwood, B.M., Bojang, K.A., and Conway, D.J. (2007). Plasmodium falciparum merozoite surface protein 3 is a target of allele-specific immunity and alleles are maintained by natural selection. J. Infect. Dis. *195*, 279–287.

Wang, L., Black, C.G., Marshall, V.M., and Coppel, R.L. (1999). Structural and antigenic properties of merozoite surface protein 4 of Plasmodium falciparum. Infection and
