# Supplementary material for: Human Antibodies Fix Complement to Inhibit Plasmodium falciparum Invasion of Erythrocytes and Are Associated with Protection against Malaria
Source: Immunity. 2015 Mar 17;42(3):580–90. doi: 10.1016/j.immuni.2015.02.012 (PMC4372259; doi:10.1016/j.immuni.2015.02.012)
Supplement: Document S2. Article plus Supplemental Information [file mmc2.pdf]

Article

Immunity

Human Antibodies Fix Complement to Inhibit *Plasmodium falciparum* Invasion of Erythrocytes and Are Associated with Protection against Malaria

Graphical Abstract

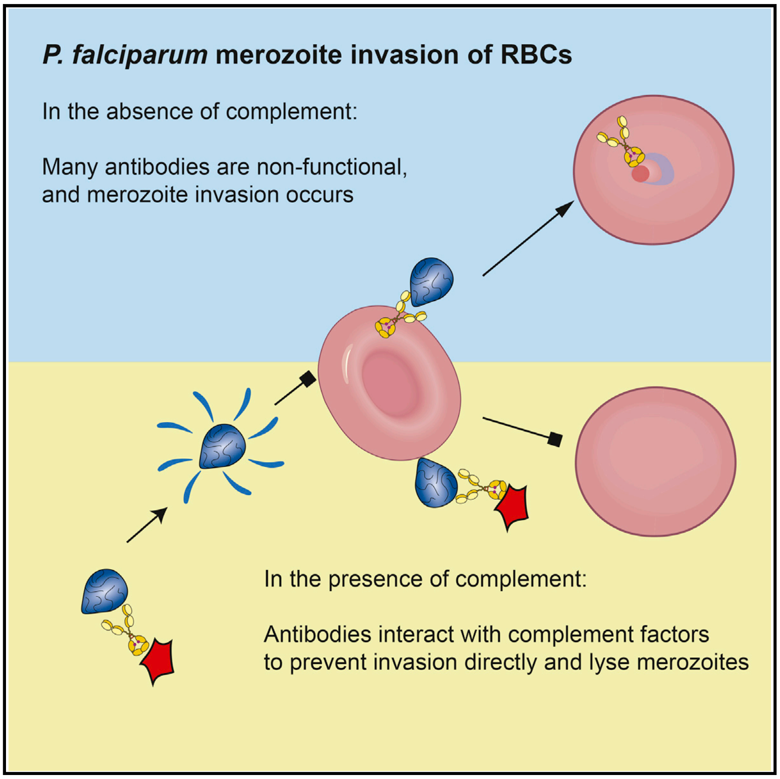

Highlights

- Antibodies function with complement to inhibit *P. falciparum* replication
- Antibodies fix C1q to block invasion and lyse merozoites
- Complement-fixing antibodies are strongly associated with immunity in children
- Antibody-complement inhibition can be induced by human vaccination

Authors

Michelle J. Boyle, Linda Reiling, ..., Robin F. Anders, James G. Beeson

Correspondence

beeson@burnet.edu.au

In Brief

Antibodies are important in immunity to malaria, but their protective function has been unclear. Boyle and colleagues report that acquired and vaccine-induced human antibodies recruit complement to block infection of erythrocytes and blood-stage replication of *Plasmodium falciparum*.

Boyle et al., 2015, Immunity *42*, 580–590
March 17, 2015 ©2015 The Authors
http://dx.doi.org/10.1016/j.immuni.2015.02.012

Immunity
Article

Human Antibodies Fix Complement to Inhibit *Plasmodium falciparum* Invasion of Erythrocytes and Are Associated with Protection against Malaria

Michelle J. Boyle,[1,2] Linda Reiling,[1] Gaoqian Feng,[1] Christine Langer,[1] Faith H. Osier,[3] Harvey Aspeling-Jones,[4] Yik Sheng Cheng,[1,2] Janine Stubbs,[1] Kevin K.A. Tetteh,[4] David J. Conway,[4] James S. McCarthy,[5] Ivo Muller,[6] Kevin Marsh,[3] Robin F. Anders,[7] and James G. Beeson[1,8,*]
[1]The Burnet Institute for Medical Research and Public Health, 85 Commercial Road, Melbourne, VIC 3004, Australia
[2]Department of Medical Biology, University of Melbourne, Royal Parade, Melbourne, VIC 3010, Australia
[3]Centre for Geographic Medicine Research, Kenya Medical Research Institute, Coast, PO Box 230, 80108 Kilifi, Kenya
[4]London School of Hygiene and Tropical Medicine, Keppel Street, London WC1E7HT, UK
[5]QIMR Berghofer Medical Research Institute, University of Queensland, 300 Herston Road, Herston, QLD 4006, Australia
[6]Walter and Eliza Hall Institute, Royal Parade, Melbourne, VIC 3050, Australia
[7]Department of Biochemistry, La Trobe Institute for Molecular Science, La Trobe University, Melbourne, VIC 3086, Australia
[8]Department of Microbiology, Monash University, Clayton, VIC 3800, Australia
*Correspondence: beeson@burnet.edu.au
http://dx.doi.org/10.1016/j.immuni.2015.02.012
This is an open access article under the CC BY license (http://creativecommons.org/licenses/by/4.0/).

SUMMARY

Antibodies play major roles in immunity to malaria; however, a limited understanding of mechanisms mediating protection is a major barrier to vaccine development. We have demonstrated that acquired human anti-malarial antibodies promote complement deposition on the merozoite to mediate inhibition of erythrocyte invasion through C1q fixation and activation of the classical complement pathway. Antibody-mediated complement-dependent (Ab-C′) inhibition was the predominant invasion-inhibitory activity of human antibodies; most antibodies were non-inhibitory without complement. Inhibitory activity was mediated predominately via C1q fixation, and merozoite surface proteins 1 and 2 were identified as major targets. Complement fixation by antibodies was very strongly associated with protection from both clinical malaria and high-density parasitemia in a prospective longitudinal study of children. Ab-C′ inhibitory activity could be induced by human immunization with a candidate merozoite surface-protein vaccine. Our findings demonstrate that human anti-malarial antibodies have evolved to function by fixing complement for potent invasion-inhibitory activity and protective immunity.

INTRODUCTION

Humoral responses to *Plasmodium falciparum* are an important component of acquired immunity against malaria, as demonstrated in pivotal studies in which immunoglobulin G (IgG) from immune adults was transferred to malaria-infected children and resulted in parasite clearance and recovery (Cohen et al., 1961). Antibodies are thought to protect by inhibiting blood-stage replication and preventing high-density parasitemia. However, specific mechanisms of protection are not well understood. The merozoite stage, which infects red blood cells (RBCs), is an important target, and antibodies to some merozoite antigens can inhibit *P. falciparum* replication in vitro (Hodder et al., 2001; Miura et al., 2009; Reiling et al., 2012; Wilson et al., 2011). However, antibodies targeting numerous merozoite antigens, including vaccine candidates such as MSP2 and MSP3, lack activity in these standard assays (McCarthy et al., 2011; Oeuvray et al., 1994), despite some evidence of efficacy in clinical and pre-clinical trials (Genton et al., 2002; Sirima et al., 2011). Indeed, growth-inhibitory activity of human antibodies is not consistently predictive of clinical immunity (Crompton et al., 2010; Dent et al., 2008; Marsh et al., 1989; McCallum et al., 2008), and antibodies from immune adults often fail to inhibit parasite replication in standard assays (Dent et al., 2008; McCallum et al., 2008; Shi et al., 1999). A lack of established immune correlates of protection severely hampers the evaluation and prioritization of vaccines (Beeson et al., 2014).

Overall reactivity of antibodies to merozoite antigens as measured by ELISA correlates with protection in some, but not all, human studies (Fowkes et al., 2010). Human antibodies to merozoite antigens are predominantly cytophilic subclasses IgG1 and IgG3; these have been associated with protection from malaria (Polley et al., 2006; Richards et al., 2010; Roussilhon et al., 2007; Stanisic et al., 2009; Taylor et al., 1998). This raises the question of whether complement might be an important effector of antibody function. Although complement activation has been reported in malaria infection and innate activation has been implicated in pathogenesis (reviewed in Biryukov and Stoute, 2014), the role of complement in antibody-mediated protection has not been defined.

Here, we developed approaches and assays to determine the ability of acquired human antibodies to fix complement and inhibit merozoite invasion of RBCs and to identify major merozoite targets of these antibodies. We evaluated antibody activity

in naturally exposed individuals from diverse geographic regions and vaccinated humans, and we obtained epidemiologic evidence supporting a role for antibody-mediated complement fixation in protective immunity to malaria in children. Our findings represent a major advance in understanding immunity to malaria and provide a much-needed strategy for the development and evaluation of vaccines.

RESULTS

Human IgG from Malaria-Exposed Donors Has Complement-Dependent Inhibitory Activity

To assess the role of complement in antibody inhibition of invasion, we performed merozoite-invasion assays in the presence or absence of active complement (Boyle et al., 2010b; Figures S1A and S1B). Merozoites were isolated from schizonts via membrane filtration and incubated with uninfected RBCs together with increasing concentrations of purified IgG (1/200 to 1/10 dilution) from malaria-exposed pooled donors (from Kenya and Papua New Guinea [PNG]) in the presence of either normal serum (NS; complement active) or heat-inactivated serum (HIS; complement inactive). IgG from Kenyan donors was non-inhibitory in HIS but effectively inhibited invasion when incubated with NS (Figure 1A). IgG from PNG donors had some activity in HIS, but inhibition was much greater in NS (Figure 1A). IgG from malaria-naive donors (Australian residents) was not inhibitory in NS or HIS, and the fact that NS did not inhibit in the absence of IgG indicates that complement alone is non-inhibitory (Figures S1C and S1D). The greater inhibition of merozoite invasion by malaria-exposed IgG in NS than in HIS suggests that IgG interacts with complement to inhibit invasion. This identifies an invasion-inhibitory mechanism that we refer to as antibody-mediated complement-dependent (Ab-C′) inhibition. We will refer to inhibitory activity of antibodies in the absence of complement (HIS) as direct antibody inhibition.

Complement fixation on merozoites incubated with malaria-exposed IgG (PNG residents) or malaria-naive IgG (Australian residents) was investigated via immunoblot. C1q and C3b were detected at higher levels on merozoites incubated with NS and PNG IgG than on merozoites incubated with Australian IgG, reflecting activation of the classical complement cascade by anti-merozoite antibodies (Figure 1B; Figures S1E and S1F). Some C3b deposition was detected on merozoites incubated with Australian IgG and NS, suggesting activation of the antibody-independent alternate pathway. C3b deposition was confirmed by immuno-electron microscopy; merozoite surface-bound C3b was detected after incubation with PNG IgG and NS, but not with HIS (Figure 1C; Figure S1G). The formation of the membrane attack complex (MAC; components C5–C9) was detected by immuno-fluorescence (IF) microscopy. MAC formation was detected on merozoites incubated with PNG IgG and NS. Lower MAC formation was seen with Australian IgG and NS (Figure 1D). Using an ELISA-based assay, we quantified MAC deposition and demonstrated that it was significantly higher ($p < 0.01$) with PNG IgG than with Australian (Melbourne) IgG (Figure 1E). These data show that human anti-malarial antibodies enhance complement deposition on merozoites via C1q fixation and thus result in increased C3b deposition and MAC formation.

C1q Fixation Mediates Ab-C′ Inhibition

To test the importance of the activation of the classical complement cascade in relation to that of the alternative complement cascade, we compared the invasion-inhibitory activity of anti-malarial antibodies between NS and HIS heated at 50°C for 20 min; this treatment inhibits the alternative complement cascade by inactivating Factor B but leaves the classical complement cascade intact. The 50°C treatment of serum had no significant effect on the invasion-enhancing activity; invasion inhibition by PNG IgG was greater in the presence of NS and 50°C-treated serum than in the presence of standard HIS (Figure S2A). This indicates that amplification of the alternative pathway does not account for Ab-C′ inhibition. To address the relative importance of different complement components, we tested invasion inhibition in C1q- and C5-depleted serum and reconstituted serum. There was significant enhancement of invasion inhibition by PNG IgG in assays with C1q-reconstituted serum in comparison to assays with C1q-depleted serum, which was comparable to the greater inhibition in NS than in HIS (Figure 2A). In contrast, there was significantly less of a difference in the extent of PNG IgG inhibition in C5-reconstituted serum than in C5-depleted serum. This suggests that activation of the classical complement cascade, specifically fixation of C1–C4, might be sufficient to mediate the majority of Ab-C′ and that deposition of C5–C9 is of less importance. To investigate this further, we incubated merozoites with PNG or Australian IgG in increasing concentrations of purified human C1q in the absence of other complement factors. C1q substantially enhanced the inhibitory activity of PNG IgG (Figure 2B), indicating that C1q fixation alone was sufficient to mediate substantial Ab-C′ inhibition. No inhibition was seen in the presence of control IgG or C1q.

To investigate whether MAC deposition and lysis could also contribute to inhibiting invasion, we assessed the ability of IgG and complement to lyse merozoites. Merozoite invasion occurs rapidly, such that 80% of invasion occurs within 10 min of mixing with RBCs (Boyle et al., 2010b); therefore, we assessed the lysis of merozoites within this time period. Merozoites were incubated with PNG or Australian IgG and 20% NS or HIS and counted by flow cytometry (Figure 2C; Figure S2B). PNG IgG mediated a significantly greater reduction in merozoites in NS than in HIS, whereas there was little lysis of merozoites with Melbourne IgG. Over an extended time period (30–60 min), lysis of merozoites was observed in NS alone (Figure S2C), consistent with the low rate of MAC formation in the absence of malaria-specific IgG (Figure 1E). Rapid merozoite lysis was dependent on activation of the classical complement cascade and was not an artifact of agglutination, as confirmed by the lack of lysis in C1q-depleted serum (Figure S2D). Using PNG IgG and 20% NS, analysis of the timing of merozoite lysis revealed that lysis occurred rapidly, such that the majority occurred within 2–3 min of incubation and reached a maximum by 4 min (Figure 2D).

Ab-C′ Inhibition Is the Predominant Mechanism Targeting Merozoite Invasion

The importance of Ab-C′ inhibition in naturally acquired immunity was assessed with purified IgG from Kenyan ($n = 33$) and PNG ($n = 10$) individuals. Overall, there was much greater Ab-C′ inhibition than direct inhibitory activity (Figure 3A). Compared to direct inhibition, Ab-C′ inhibitory activity was seen in a

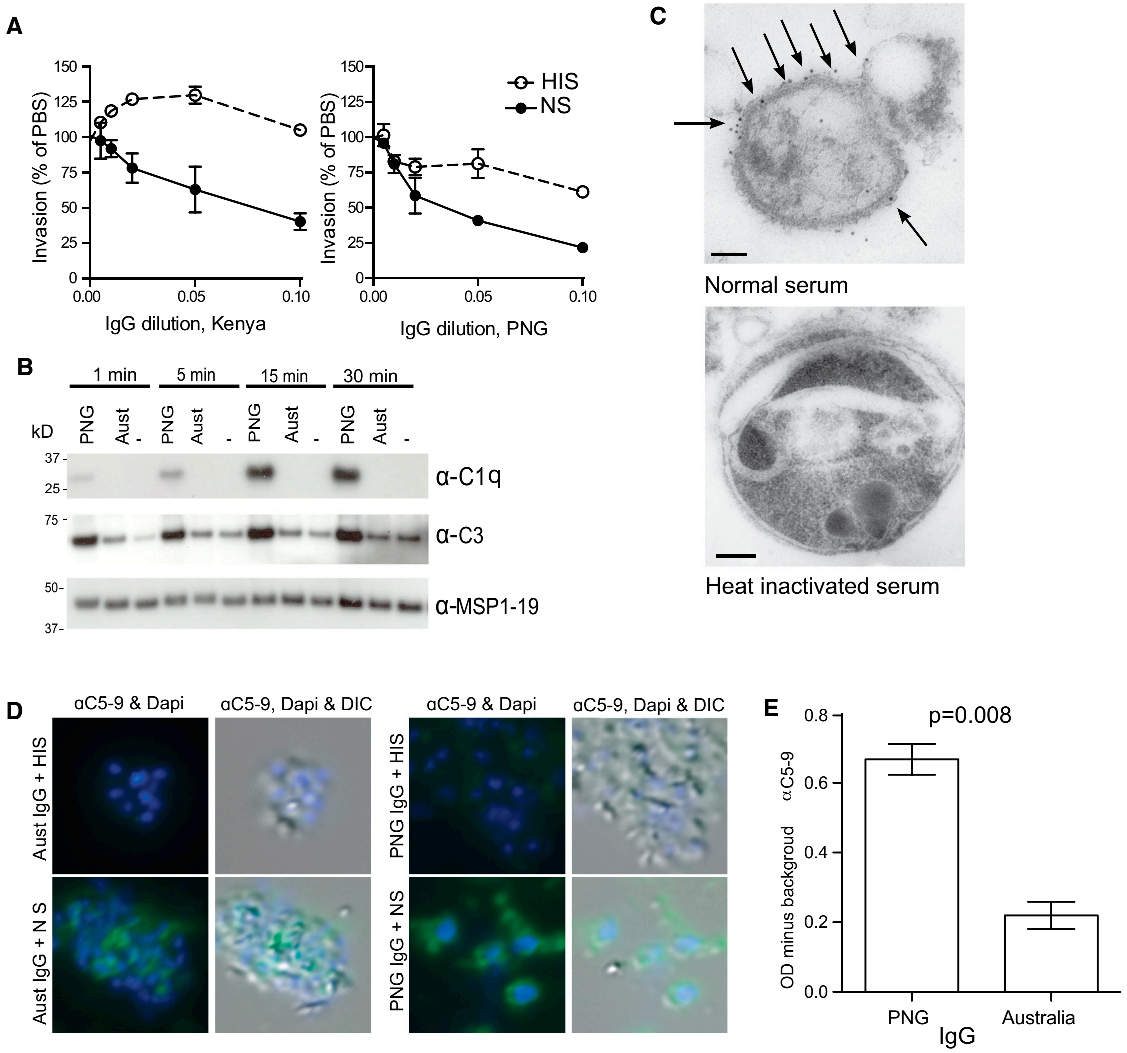

Figure 1. Invasion Inhibition by IgG and Complement and Complement Deposition on the Merozoite Surface

(A) Invasion-inhibitory activity of purified IgG from Kenya and PNG was tested in invasion assays performed with 50% normal serum (NS; complement active) or heat-inactivated serum (HIS; complement inactivated). Data represent the mean ± range from two independent assays performed in duplicate.

(B) C1q and C3 deposition on merozoites incubated with purified PNG IgG, purified malaria-naive IgG (Australian donors), or PBS together with 25% NS for 1, 5, 15, and 30 min. MSP1-19, a merozoite surface protein, was used as a loading control.

(C) C3b deposition on merozoites incubated with purified PNG IgG and 25% NS or HIS via immuno-electron microscopy. Gold labeling is indicated with arrows. Scale bars represent 0.1 μm.

(D) Formation of the membrane attack complex (MAC; complement components C5–C9) on merozoites incubated with purified PNG or Australian (Melbourne) IgG and 25% NS or HIS via IF microscopy.

(E) MAC deposition as quantified by ELISA on merozoites incubated with NS and PNG or Australian IgG. Immunoblots and microscopy images are representative of two independent experiments.

See also Figure S1.

much greater proportion of individuals (proportion positive [defined as >15% inhibition]: 57.2% ± 8.7% for Ab-C′ and 21.2% ± 7.2% for direct inhibition; $p < 0.01$). These striking results reveal that the majority of human antibodies require complement factors to effectively inhibit merozoite invasion. The extent of inhibitory activity varied widely. In Kenyan individuals, four activity profiles were observed: (1) no inhibitory activity in NS or HIS (11/33 [33%]); (2) invasion enhancement in HIS, but not NS (7/33 [21%]); (3) Ab-C′ inhibition only, demonstrated by invasion inhibition in NS, but not HIS (10/33 [30%]); and (4) a combination of Ab-C′ inhibition and direct inhibitory activity, demonstrated by inhibition in HIS and increased inhibition in NS (5/33 [15%]) (Figure 3B; Figure S3A). Among PNG individuals, 50% had Ab-C′ inhibitory activity only, and the remaining samples had substantial direct inhibitory activity (Figure 3C). Overall, 37% of samples had only complement-dependent inhibitory antibodies. In those with both complement-dependent and directly active antibodies, complement-dependent inhibition ranged from 12% to 81% of the total inhibitory response.

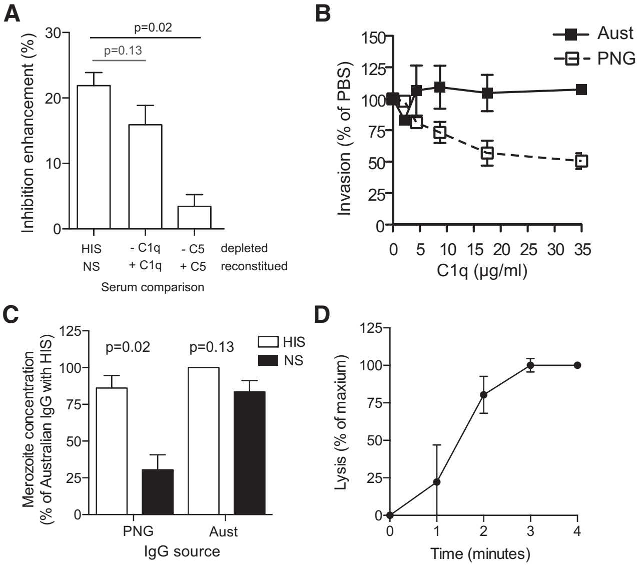

Figure 2. C1q Fixation by IgG Inhibits Invasion, and Complement Fixation Leads to Merozoite Lysis

(A) Invasion-inhibitory activity of purified PNG IgG (1/10 dilution) was tested in the presence of 25% NS and HIS, C1q-depleted serum with and without reconstitution with purified C1q, and C5-depeleted serum with and without reconstitution with C5. The difference in invasion-inhibitory activity between depleted and reconstituted serum was calculated. Data represent the mean ± SEM from four to five independent assays performed in duplicate.

(B) Invasion-inhibitory activity of purified PNG IgG in the presence of increasing concentrations (2.2–35 μm/ml) of purified C1q. Data show invasion as a percentage of that of media alone and represent the mean ± range from two independent assays performed in duplicate.

(C) Lysis of merozoites: merozoite concentration after 10-min incubation with 1/20 dilution of purified PNG or purified malaria-naive Australian IgG and 20% NS or HIS. Data show merozoite concentration as a percentage of that of purified Australian IgG with HIS and represent the mean ± SEM from three independent assays performed in duplicate.

(D) Lysis rate of merozoites incubated with purified PNG IgG and 20% NS. Data show lysis as a percentage of the maximum and represent the mean ± SEM from four independent assays.

See also Figure S2.

Ab-C′ Inhibition Strongly Correlates with Cytophilic Antibodies to Merozoites

In Kenyan individuals, we assessed the relationship among Ab-C′ inhibition, direct inhibition, IgG subclass reactivity to merozoites, and antibody activity in standard complement-free growth-inhibition assays (GIAs). The prevalence of antibodies to merozoites was high, and merozoite-specific IgG was strongly and significantly correlated with Ab-C′ inhibition, but not with direct inhibitory activity and less strongly with activity in GIAs (Table 1). Of note, the strongest correlation was between Ab-C′ inhibition and IgG3 (Figure 3D). This relationship was stronger than that seen for IgG1 and is consistent with the known property of IgG3 as the most potent activator of complement. Ab-C′ inhibition was also strongly correlated with age (Spearman's r = 0.63, p = 0.0003), matching the acquisition of immunity. In contrast, direct inhibitory activity only weakly correlated with age (Spearman's r = 0.17, p = 0.38). With a median invasion in NS of 32.3% (95% CI = 54.7–82.2), Ab-C′ activity was also greater than activity in GIAs, whose median growth was 89.3% (95% CI = 81–91, p = 0.007). Further, the proportion of individuals with positive Ab-C′ inhibition increased with age, and 100% of adults had Ab-C′ inhibitory activity; only 44% had direct activity (Figure 3E). These results strongly suggest that Ab-C′ is the predominant mechanism of antibodies targeting merozoite invasion and is acquired in naturally exposed individuals as immunity to malaria develops.

C1q Fixation by Antibodies Correlates with Ab-C′ Inhibition

Having shown that Ab-C′ inhibition functions via fixation of C1q and the activation of the classical complement cascade, we evaluated the relationship between C1q deposition and Ab-C′ inhibition. We measured antibody-mediated C1q deposition on merozoites by immunoblot using IgG from nine Kenyan individuals with high, medium, or low Ab-C′ inhibition (n = 3 for each group). C1q deposition was notably higher with IgG from individuals who had high Ab-C′ inhibitory activity than with IgG from those with medium or low activity (Figure 3F). Next, to quantify C1q fixation on merozoites, we developed a high-throughput plate-based assay that uses small sample volumes to allow testing of large numbers of serum samples and that can be used with small-volume pediatric samples (Figure S3B). Antibody-mediated C1q deposition on merozoites was strongly correlated with Ab-C′ activity (Figure 3G). Samples that promoted high C1q fixation also had high C3b fixation (Figure S3C). This supports the important role of C1q fixation and activation of the classical cascade in Ab-C′ inhibition and establishes an efficient complement-deposition assay that is suitable for application to clinical studies.

Antibodies that Fix Complement Are Associated with Protection from Malaria

To obtain epidemiologic evidence of the importance of antibody-mediated complement fixation in acquired immunity to malaria, we tested antibodies for C1q fixation from a longitudinal cohort of 206 5- to 14-year-old children who were resident in a malaria-endemic region of PNG (Michon et al., 2007); all children were treated for malaria parasitemia at enrollment and then monitored by active surveillance for parasitemia and clinical malaria over 6 months of follow-up. The prevalence of antibody-mediated C1q fixation on the merozoite surface was very high (Table S1), reflective of substantial exposure to malaria in this population. Antibody-mediated C1q deposition was associated with age, such that older children had significantly higher C1q deposition than younger children (Figure 4A; Figure S4). C1q deposition was also higher in children who were parasitemic at the time of sample collection than in aparasitemic children (Figure 4B). The increase in antibody-dependent C1q deposition with age and parasitemia is consistent with the expected acquisition of immunity.

To assess the role of antibody-mediated C1q fixation in protection from symptomatic malaria and high-density parasitemia, we grouped children into low, medium, and high categories of C1q-fixation activity; we then compared the relative risk of malaria between response groups. High C1q deposition was very strongly associated with protection from clinical malaria (Table 2). The association between antibodies and protection from malaria appeared to have a dose-response relationship

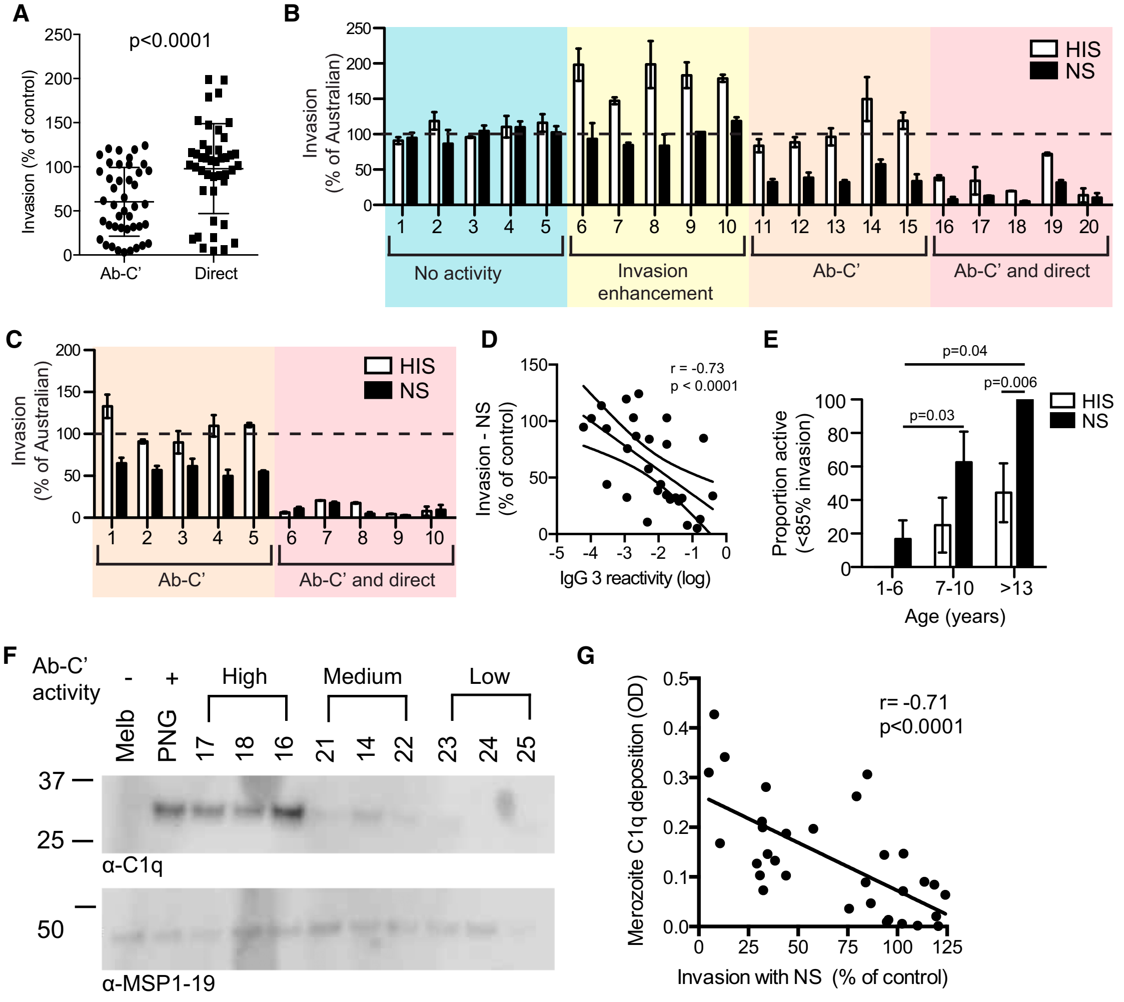

Figure 3. Ab-C′ Is the Predominant Mechanism of Naturally Acquired Antibodies and Correlates with C1q-Deposition Activity

(A) Invasion-inhibitory activity of purified IgG from Kenyan and PNG individuals in 50% NS or HIS (median ± interquartile range).

(B) Invasion-inhibitory activity of purified IgG from Kenyan individuals in the presence of NS and HIS; shown are no inhibitory activity (blue), invasion-enhancement activity (in HIS and not NS, yellow), Ab-C′ inhibition (orange), and Ab-C′ and direct inhibitory activity (red). Data represent the mean ± range from two independent assays performed in duplicate.

(C) Invasion-inhibitory activity of purified IgG from PNG individuals.

(D) IgG3 reactivity to the merozoite surface as measured by ELISA correlated with functional activity in Ab-C′ assays. Data show invasion as a percentage of that of the control.

(E) Significant activity in invasion-inhibition assays with 50% NS or HIS was defined as <85% invasion (>15% inhibition); data show the proportion of individuals with activity, and individuals are stratified by age. p values are shown for comparisons of the proportion of samples that were positive in assays using NS between the 1–6 and 7–13 age groups and between the 1–6 and >13 age groups. The p value is also shown for the comparison of the proportion of positive samples in the >13 age group between assays using NS and assays using HIS.

(F) C1q deposition on merozoites was assessed by immunoblot for nine purified Kenyan IgG samples that had high, medium, or low Ab-C′ inhibitory activity. The image is representative of two independent assays. Abbreviations are as follows: Melb, pool of Melbourne IgG included as a negative control; PNG, pool of PNG IgG included as a positive control.

(G) Measured by ELISA, C1q deposition on merozoites in purified Kenyan IgG samples correlated with Ab-C′ inhibitory activity (n = 33).

See also Figure S3.

(Figure 4C); high responders had fewer symptomatic episodes than did medium or low responders. Numerous variables were explored as possible confounding factors; only subject age and location of residence were significantly associated with risk of malaria (Michon et al., 2007). The strong protective association for antibody-mediated C1q deposition remained after adjustment for age and location of residence ($p < 0.0001$), and protective associations were observed for children who were parasitemic or aparasitemic at enrollment (Figure S4B). High C1q deposition was also strongly associated with protection from episodes of high-density parasitemia (>5,000 parasites/μl), which remained significant after adjustment for confounders (Table 2; Figure 4D). These data are consistent with a role for antibody-mediated complement fixation and Ab-C′ inhibition in limiting blood-stage replication of *P. falciparum* and preventing disease.

Table 1. Correlation between Invasion-Inhibition Assays and GIAs and Antibodies to the Merozoite Surface

		Functional Activity[a]					
	Antibody Prevalence[b]	IIA-Ab-C′		IIA-Direct		GIA	
		r[c]	p	r[c]	p	r[c]	p
Total IgG	82%	0.57	<0.001	0.18	0.31	0.41	0.02
IgG1	85%	0.56	<0.001	0.13	0.49	0.32	0.07
IgG2	55%	0.46	0.01	0.37	0.04	0.52	0.001
IgG3	52%	0.73	<0.0001	0.16	0.37	0.48	0.005
IgG4	3%	0.49	0.07	0.07	0.70	0.3	0.09

[a]Functional activity of individuals was measured in invasion-inhibition assays with NS (IIA-Ab-C′) and HIS (IIA-direct) and in a standard GIA that measures growth-inhibitory activity over two invasion cycles in complement-free conditions.
[b]Thirty-three Kenyan serum samples were tested for total IgG, IgG1, IgG2, IgG3, and IgG4 on the merozoite surface by ELISA. Positive responses were defined as greater than the mean optical-density values of Australian (Melbourne) controls plus 3 SDs.
[c]Spearman correlation coefficients.

MSP1 and MSP2 Are Targets of Ab-C′ Inhibitory Antibodies

To identify merozoite antigens that are targets of Ab-C′ inhibition, we tested rabbit antibodies to several major merozoite surface antigens for inhibition of invasion in the presence of NS or HIS. Antibodies to MSP1-19, MSP1 block 2, and MSP2 substantially inhibited invasion in the presence of NS, but not HIS (Figure 5A). Activity was specific, and no inhibition was seen with antibodies from non-immunized rabbits (data not shown). Antibodies to the MAD20-like MSP1 block 2 alleles used in the parasite line tested (MAD20 and Wellcome alleles) inhibited invasion, whereas antibodies to heterologous K1-like (3D7 or Palo Alto) alleles did not, reflecting the strain specificity of the antibodies. It is notable that some antibodies to MSP2 and MSP1 block 2 enhanced invasion in HIS, as was seen with some antibodies from naturally exposed subjects (Figure 3), whereas they were inhibitory in the presence of NS. With rabbit antibodies to MSP3, MSP4, and AMA1, we observed minimal differences in invasion-inhibitory activity between NS and HIS.

MSP2 and MSP3 are vaccine candidates that have progressed to clinical trials, but development has been hampered by the lack of immunologic correlates of protection, given that these antibodies are relatively non-inhibitory in standard GIAs (McCarthy et al., 2011; Oeuvray et al., 1994). The function of human antibodies to MSP2 and MSP3 was defined with affinity-purified antigen-specific human antibodies in invasion-inhibition assays (Figure 5B). In agreement with results from rabbit antibodies, purified human anti-MSP2 antibodies significantly inhibited invasion in NS, but not in HIS. In contrast, MSP3 antibodies showed a limited amount of direct inhibitory activity and no enhancement by complement.

To further confirm the role of complement fixation in mediating invasion inhibition and the significance of MSP2 as a target, we tested a human MSP2-specific monoclonal antibody (mAb) with and without a specific change (L234A or L235A [LALA]) in the amino acid sequence of the Fc region (Stubbs et al., 2011); this change ablates binding to C1q and complement activation but leaves binding to the antigen unaffected (Hessell et al., 2007). Significantly greater invasion inhibition in NS than in HIS was only seen with wild-type MSP2 mAb and not with the modified mAb. Further, invasion inhibition was greater in NS with the wild-type than in NS with the altered mAb. These results further confirm the importance of C1q fixation in Ab-C′ inhibition and MSP2 as a target.

Ab-C′ Inhibitory Antibodies Can Be Induced by Human Immunization

We examined whether Ab-C′ inhibitory activity could be induced by immunization of malaria-naive individuals with recombinant merozoite surface proteins. We studied samples from the recent phase 1 clinical trial of the MSP2-C1 vaccine (McCarthy et al., 2011). IgG from ten individuals with high C1q-fixation activity (as defined by ELISA) were tested for invasion-inhibitory activity in NS and HIS (Figures 5D and 5E). IgG from these individuals lacked inhibitory activity in standard GIAs despite high antibody reactivity by ELISA (McCarthy et al., 2011). Notably, eight of ten individual IgG samples showed substantial inhibition in NS, but not in HIS, indicating the induction of Ab-C′ inhibition by vaccination. No inhibition was seen in IgG from pre-vaccinated individuals or placebo-vaccinated samples (Figure S5). These data indicates that MSP2 antibodies induced by vaccination are able to inhibit invasion via Ab-C′ inhibition and identify a potential mechanism mediating the protective efficacy of MSP2-based vaccines (Genton et al., 2002).

DISCUSSION

Although the importance of antibody in immunity to malaria has been established (Cohen et al., 1961), mechanisms mediating protection are poorly understood. Here, we provide evidence that complement plays a key role in antibody-mediated immunity to malaria in humans. Antibodies from malaria-exposed individuals enhanced complement fixation on merozoites and had substantially greater invasion-inhibitory activity in the presence of complement. Ab-C′ inhibition was the predominant mechanism inhibiting invasion, and many antibodies were only inhibitory in the presence of complement factors. Our findings indicate that the mechanism underlying this activity is predominately mediated by C1q fixation. Antibody-complement interactions also led to merozoite lysis. Targets of Ab-C′ include the most abundant merozoite surface antigens, MSP1 and MSP2. Furthermore, we provide epidemiologic evidence of the role of antibody-complement interactions in human immunity by demonstrating that C1q fixation was very strongly associated with protection from clinical malaria and high-density parasitemia in a prospective longitudinal study of children. Finally, we demonstrated that Ab-C′ inhibition can be induced by human immunization, providing an important proof of concept for translation into malaria vaccine development.

Comparisons of Ab-C′ inhibitory activity in serum depleted and reconstituted with C1q or C5 highlight the importance of C1q in mediating inhibition of invasion. Further, C1q alone was able to significantly enhance the inhibitory activity of antimalarial antibodies in the absence of other complement factors. This was further demonstrated by comparison of wild-type and altered human MSP2 mAbs; Ab-C′ inhibition was only seen

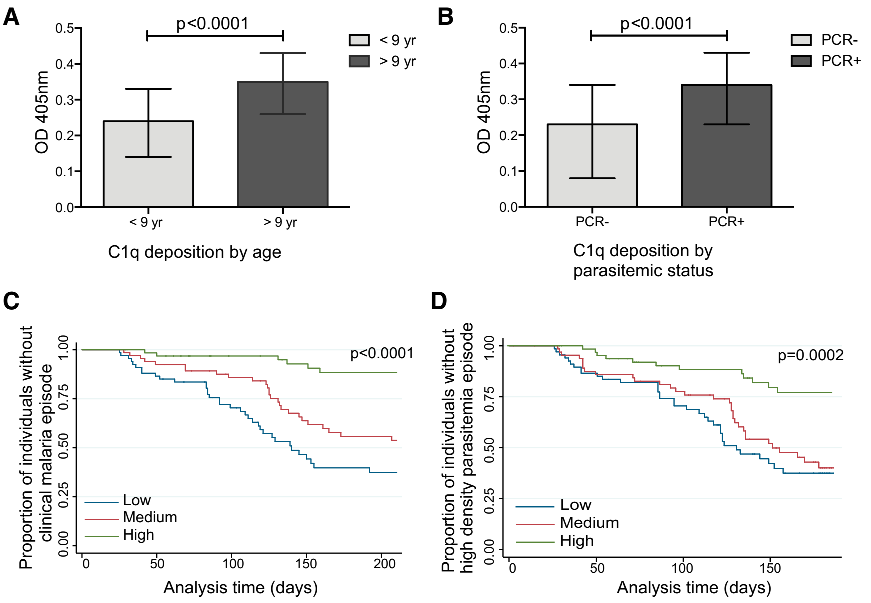

Figure 4. C1q Fixation by Antibodies Is Associated with Protection from Malaria

Antibody-mediated C1q deposition on the merozoite surface was measured in plasma from a cohort of 206 children in PNG.

(A) C1q deposition was higher in older children (>9 years; n = 115).

(B) C1q deposition was higher in children with concurrent *P. falciparum* infection (n = 139) than in uninfected children, as determined by PCR.

(C) High C1q fixation by antibodies was strongly associated with reduced risk of clinical malaria episodes. Children were divided into three groups on the basis of high, medium, and low C1q-fixing antibodies.

(D) High antibody-mediated C1q fixation was associated with reduced risk of high-density parasitemia.

See also Figure S4.

with the wild-type and not the altered mAb. C1q-mediated antibody neutralization has been previously reported with influenza (Feng et al., 2002) and West Nile virus (Mehlhop et al., 2009). Enhanced inhibition by C1q might be due to increased steric hindrance by the large (460-kDa) C1q-IgG complex blocking binding of parasite proteins to cellular receptors or through the stabilization of IgG of low avidity. In complement-free systems, some antibodies to MSP1 (Blackman et al., 1994; Dluzewski et al., 2008) and MSP2 (Boyle et al., 2014) can be internalized into the RBC while bound to the merozoite surface without inhibiting invasion. However, in the presence of complement, antibodies to MSP2 and MSP1 effectively inhibit invasion. The C1q-IgG complex might be too large to be internalized during invasion, thereby mediating the inhibitory activity of antibodies that are otherwise not directly inhibitory.

Although C1q-mediated enhancement appears central to invasion-inhibitory activity, complement deposition and lysis of merozoites are likely to have other implications in vivo, including enhancement of phagocytosis and induction of pro-inflammatory cytokines that might further mediate control of parasitemia. Complement activation, particularly as part of the innate immune response, has been implicated in pathogenesis as a result of induction of inflammatory responses (reviewed in Biryukov and Stoute, 2014; Silver et al., 2010). In the absence of anti-malarial antibodies, complement did not inhibit merozoite invasion, despite some deposition of C3b and MAC on the parasite surface and the eventual lysis of merozoites in the absence of malaria-exposed IgG with extended incubations. This is most likely due to the reduced rate and extent of complement deposition in the absence of specific antibody and might also indicate that merozoites could be able to inhibit complement activation, as described for other pathogens (Lambris et al., 2008).

Testing antibodies from various individuals demonstrated that Ab-C′ inhibition was the predominant mechanism for inhibition of invasion; the extent of inhibition and the prevalence of Ab-C′ inhibition were greater than direct antibody inhibition, and Ab-C′ inhibition increased with age, reflective of the known acquisition of immunity. Strong epidemiologic evidence of the importance of complement fixation in antibody-mediated immunity to malaria was established in a longitudinal cohort of children acquiring immunity. High-C1q-fixing antibodies were very strongly associated with protection from clinical malaria. Furthermore, complement fixation was strongly associated with protection from high-density parasitemia, consistent with the proposed role of Ab-C′ inhibition in limiting parasite replication and thereby preventing disease. These findings provide

Table 2. Association between Antibody-Mediated C1q Deposition on the Merozoite Surface and Protection from Clinical Malaria and High-Density Parasitemia

		uHR (95% CI)	p	aHR (95% CI)	p
Clinical malaria	LvM	0.56 (0.34–0.94)	0.028	0.64 (0.38–1.09)	0.1
	LvH	0.12 (0.05–0.28)	<0.0001	0.15 (0.06–0.35)	<0.0001
High-density parasitemia	LvM	0.80 (0.50–1.30)	0.37	0.94 (0.58–1.53)	0.804
	LvH	0.26 (0.13–0.49)	<0.0001	0.35 (0.18–0.70)	0.003

The cohort was stratified into three equal groups according to low, medium, or high C1q reactivity (see also Table S1). Unadjusted hazard ratios (uHRs) and HRs adjusted for age and location of residence (aHRs) were calculated to compare low-versus-medium (LvM) or low-versus-high (LvH) groups for the risk of symptomatic malaria or high-density parasitemia (>5,000 parasites/μl) over the time period of 6 months. Calculations were based on the first episode only.

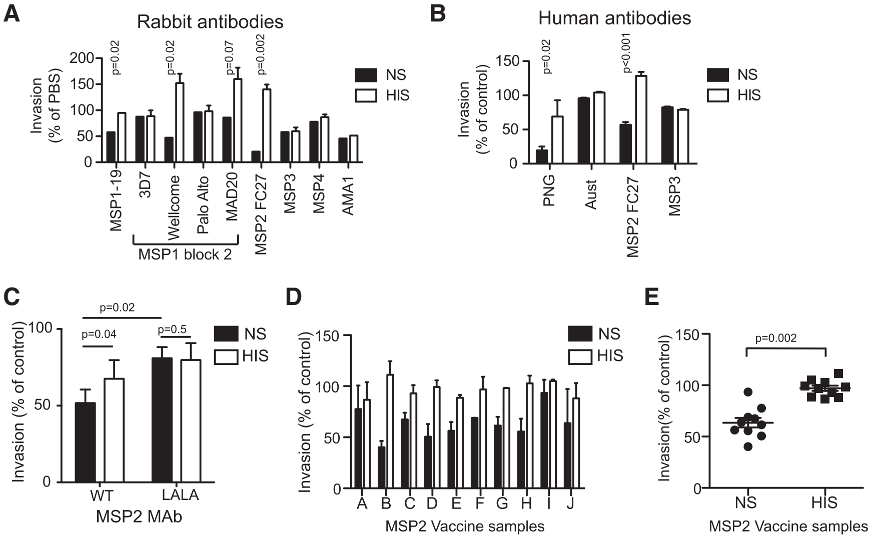

Figure 5. MSP1 and MSP2 Are Targets of Ab-C′ Inhibition, and Ab-C′ Inhibition Is Induced by Human Vaccination

(A) Invasion-inhibitory activity of rabbit antibodies specific to merozoite surface antigens in the presence of 50% NS and HIS. Data represent the mean ± range from two independent assays performed in duplicate.

(B) Invasion-inhibitory activity of naturally acquired human affinity-purified MSP2 and MSP3 antibodies at 50 μg/ml in the presence of 50% NS or HIS. Data represent the mean ± SEM from four independent assays performed in duplicate.

(C) Invasion inhibition with human mAbs (IgG1) to MSP2 in the presence of 50% NS or HIS (mAb concentration 50 μg/ml). Recombinant MSP2 mAb was expressed with the wild-type sequence or with a LALA Fc mutation, which is known to ablate C1q fixation. Two independent assays were performed in duplicate.

(D) Invasion-inhibitory activity of purified IgG from malaria-naive adults immunized with recombinant MSP2 antigen was tested in the presence of 50% NS or HIS at a 1/2 dilution of the original concentration. Data represent the mean ± range from two independent assays performed in duplicate.

(E) Overall, inhibitory activity by IgG from individuals receiving the MSP2 vaccine was greater when it was tested in the presence of 50% NS than when it was tested in the presence of HIS.

See also Figure S5.

insight into the potential role of complement fixation in protective humoral immunity in humans and contrast with the limited and inconsistent associations reported for antibody activity measured in standard GIAs. The GIA is performed in complement-free conditions and is currently the only widely used functional assay of antibodies to merozoites (Crompton et al., 2010; Dent et al., 2008; John et al., 2004; Marsh et al., 1989; McCallum et al., 2008; Wilson et al., 2011). We propose that the weak and inconsistent correlation between standard GIAs and malaria immunity reflects the central importance of complement factors in mediating antibody activity. Further, Ab-C′ inhibition strongly correlated with reactivity of cytophilic antibodies to merozoites, particularly IgG3. This fits with the properties of IgG3 as the most potent activator of complement and is consistent with findings that ELISA titers of IgG3 to merozoite antigens are associated with protection in human cohort studies (Courtin et al., 2009; Ndungu et al., 2002; Nebie et al., 2008; Richards et al., 2010; Roussilhon et al., 2007; Stanisic et al., 2009). Merozoite proteins MSP1 and MSP2 were identified as important targets of Ab-C′ inhibition. Antibodies to MSP1 block 2 and MSP2 show limited inhibition in standard GIAs (Boyle et al., 2014; Cowan et al., 2011; Flueck et al., 2009; Galamo et al., 2009; McCarthy et al., 2011). The block 2 region of MSP1 is polymorphic and under balancing selection, suggesting immune pressure. Consistent with this, the Ab-C′ inhibitory activity of MSP1 block 2 antibodies was strain specific, supporting the view that polymorphisms have evolved to mediate immune evasion.

Our studies have established a proof of principle that Ab-C′ inhibitory antibodies can be induced by human vaccination with recombinant merozoite surface antigens with the use of samples from a phase 1 trial of the MSP2-C1 vaccine. A vaccine based on MSP2 was previously found to have significant protective efficacy against *P. falciparum* parasitemia in a naturally exposed population in PNG (Genton et al., 2002). However, the mechanism of protective function of MSP2 antibodies has been unclear (McCarthy et al., 2011). Here, we have shown that antibodies to MSP2 interact with complement to inhibit invasion. The identification of Ab-C′ activity as a central protective mechanism of antibodies targeting merozoite antigens might suggest a role for this assay in evaluating candidate vaccines. Unlike the phase 2 vaccine trial of MSP2, a phase 2 trial of a MSP1 42-kDa C-terminal construct (MSP1-42) had no efficacy in a naturally exposed population in Kenya (Ogutu et al., 2009). Vaccine failure might be explained by antigenic diversity, given that only a single allele of the polymorphic MSP1-42 antigen was included, or by the nature, epitope specificity, or concentration of antibodies induced; the vaccine might have failed to induce strong complement-fixing antibodies to effectively inhibit invasion, which could be investigated in future studies.

In conclusion, we have identified Ab-C′ inhibition as a prominent mechanism targeting the merozoite in naturally acquired immunity, and we found that complement-dependent inhibition can be mediated by antibodies induced by human immunization with a recombinant merozoite surface-protein vaccine. Our findings demonstrate that human anti-malarial antibodies have evolved to function in the presence of complement by recruiting complement for functional activity and protective immunity. These insights mark a major change in our understanding of mechanisms of functional immunity and provide tools for evaluating naturally acquired and vaccine-induced immunity. Our findings might have translational implications, indicating that focusing on targets and strategies that induce strong complement-fixing antibodies might be an important step in the development of highly efficacious vaccines.

EXPERIMENTAL PROCEDURES

Further details can be found in the Supplemental Experimental Procedures.

Parasite Culture, Synchronization, and Invasion-Inhibition Assays

The *P. falciparum* D10-GFP expression line was cultured as previously described and synchronized with heparin (Boyle et al., 2010a; Wilson et al., 2010). Invasion-inhibition assays were performed as described in Boyle et al. (2010a); merozoites were incubated with uninfected RBCs, normal or heat-inactivated serum (NS or HIS, respectively), and test IgG for 30 min (Figure S1A). Cells were washed and cultured for 40 hr and then analyzed via flow cytometry. NS and HIS was from malaria-naive donors. For heat inactivation, sera were heated at 56°C for 30 min. For assays testing the importance of the alternative pathway, sera were heat inactivated at 50°C for 20 min. For assays testing the importance of C1q and C5 for Ab-C′ activity, human serum depleted of C1q or C5 and purified human C1q and C5 (Calbiochem, Merck) were used at 25% concentration. GIAs were performed as described in Persson et al. (2006).

Human Subjects and Samples

Ethical approval for the use of human serum and plasma samples in these studies was obtained from the Alfred Human Research and Ethics Committee for the Burnet Institute, from the Kenya Medical Research Institute, from the Medical Research Advisory Committee of Papua New Guinea, and from the Human Research and Ethics Committee of the Queensland Institute of Medical Research. Written informed consent was obtained from all participants or, in the case of children, from their parents or guardians. Serum pools from malaria-exposed donors were made from serum samples from Kenya (Ngerenya, Kilifi District) and PNG (Madang District). Unexposed serum pools were from Australian donors residing in Melbourne (Australia Red Cross Blood Bank). IgG from serum pools was purified with Melon Gel according to the manufacturer's (Thermo Scientific) instructions. Purified IgG was concentrated in 10-kDa MWCO (molecular weight cutoff) spin-purification tubes (Amicon) and buffer exchanged with PBS. For the longitudinal study of PNG children, plasma samples were obtained at enrollment from a prospective treatment-reinfection cohort of 206 children aged 5–14 years (median = 9.3) in Madang, PNG (Michon et al., 2007). Children were actively reviewed every 2 weeks for symptomatic illness and parasitemia, and by passive case detection, over a period of 6 months. A clinical episode of *P. falciparum* malaria was defined as fever and *P. falciparum* parasitemia >5,000 parasites/μl. Serum samples were used from a phase 1 MSP2-C1 vaccine trial in which participants were immunized with both 3D7 and FC27 MSP2 isoforms formulated with Montanide ISA 720 (McCarthy et al., 2011) (sponsored by PATH Malaria Vaccine Initiative; Trial Registration, AWZCTR 12607000552482).

Rabbit and Human Antibodies to Specific Merozoite Antigens

Rabbit sera were raised against recombinant proteins corresponding to MSP1-19, MSP1 block 2, MSP4, MSP2, the MSP3 C-terminal region, and AMA1 (3D7 and 7G8 alleles) as described in Boyle et al. (2014) and Drew et al. (2012). Human antibodies to MSP2 (FC27) and MSP3 (K1) were purified from PNG and Kenyan serum donors via column chromatography according to established methods (Reiling et al., 2012). A human mAb to MSP2 was previously isolated from a malaria-exposed donor and expressed as recombinant IgG1 with the wild-type sequence or with a Fc-LALA mutation (Stubbs et al., 2011).

Complement Deposition Assays via Immunoblot, ELISA, and Microscopy

Merozoites were incubated with 25% NS and test IgG or serum samples for 1, 5, 10, 15, or 30 min at 37°C. Merozoites were washed and solubilized for immunoblot analysis. C1q and C3 were detected with anti-C1q (Goat polyclonal, Calbiochem, Merck) and anti-C3 (HRP-conjugated goat polyclonal, MP Biomedicals), respectively.

For ELISA, plates were coated with purified merozoites at 5×10^6 merozoites/well. Plates were blocked and then incubated with sera samples (1/250), and then recombinant C1q (10 μg/ml) and C1q were detected with goat anti-C1q antibodies and anti-goat-HRP. C1q and C3 deposition was also detected with C5-depleted serum as a complement source. For ELISA analysis of MAC deposition, isolated merozoites were incubated with 25% NS serum and IgG from PNG or Australian (Melbourne) serum pools for 10 min at 37°C and then washed and coated into Nunc 96-well plates. Plates were blocked, and the presence of MAC was detected with anti-C5–C9 antibodies (rabbit) followed by anti-rabbit-HRP.

For immuno-electron microscopy, merozoites were incubated with NS or HIS with PNG IgG for 10 min. Merozoites were washed and fixed in 1% glutaraldehyde and then processed and imaged as described in Boyle et al. (2010a). For IF microscopy, merozoites were incubated with 25% NS serum and IgG from PNG or Melbourne donors for 10 min (37°C). Merozoites were washed and dried on slides, fixed with methanol, and blocked, and then MAC was detected with anti-C5–C9 antibodies (rabbit) and anti-rabbit-Alexa 488 antibodies. Images were obtained as described in Reiling et al. (2012).

Assays of Merozoite Lysis

Merozoites were incubated with 5% PNG or Melbourne IgG and 20% NS, HIS, or C1q-depleted serum for 10 min (37°C); for assays to assess the rate of merozoite lysis, an aliquot of sample was taken every minute for analysis by flow cytometry. Samples were diluted 1/100 in 200 μl cold PBS and 1% newborn calf serum, and the density of merozoites was counted with Count-Bright counting beads via flow cytometry.

ELISA to Intact Merozoites

ELISAs were performed according to standard methods (Stanisic et al., 2009). Purified merozoites were coated in PBS and placed on microtiter plates. Merozoites were blocked and subsequently incubated with Kenya serum samples diluted at 1/250 and then sheep anti-human IgG HRP diluted at 1/2,500.

Data Analysis

Differences in invasion-inhibitory IgG activity between NS and HIS and between C1q-depleted and -reconstituted serum were calculated with paired t tests in Stata/SE 11.2. Associations between antibody reactivity to intact merozoites by ELISA and functional activity in assays of Ab-C′ inhibition, direct inhibition, and growth inhibition were assessed with Spearman's correlations calculated in Prism.

Analysis of the cohort study was performed with Stata/SE 12.0. Differences in C1q deposition between groups were assessed by chi-square tests (for categorical variables) or Wilcoxon rank-sum tests (for continuous variables). For assessment of associations between C1q deposition and protection, subjects were stratified into tertiles according to low (including those classified as "negative"), medium, or high deposition of C1q, as determined by optical-density values for each sample. Groups were compared for risk of clinical malaria (fever and >5,000 parasites/μl) or high-density parasitemia (>5,000 parasites/μl) with the Cox proportional-hazards model (Reiling et al., 2010; Richards et al., 2010). Survival analysis included first episodes only. Age and location of residence were previously identified as potential confounders from a range of factors (Stanisic et al., 2009).

SUPPLEMENTAL INFORMATION

Supplemental Information includes five figures, one table, and Supplemental Experimental Procedures and can be found with this article online at http://dx.doi.org/10.1016/j.immuni.2015.02.012.

AUTHOR CONTRIBUTIONS

M.J.B., L.R., G.F., and J.G.B. planned the study and interpreted results with input from all authors. M.J.B., L.R., Y.S.C., G.F., H.A.J., and C.L. performed experiments. F.H.O., K.K.A.T., D.J.C., I.M., J.S.M., K.M., J.S., and R.F.A. provided key reagents. M.J.B., L.R., G.F., and J.G.B. wrote the manuscript with input from all authors.

ACKNOWLEDGMENTS

RBCs and serum for parasite culture were provided by the Australian Red Cross Blood Bank (Melbourne). We thank the following people for reagents: D. Drew and A. Hodder (AMA1 antibodies), R. Coppel and B. Cooke (MSP4 antigen and MSP4 antibodies), and B. Crabb and P. Gilson (MSP1-19 antibodies). Funding was provided by the National Health and Medical Research Council of Australia (program grant to J.G.B.; Infrastructure for Research Institutes Support Scheme grant and research fellowships to J.G.B. and J.S.M.),

the Australian Research Council (Future Fellowship to J.G.B.), a Victorian State Government Operational Infrastructure Support grant, the Queensland Government (Medical Research Fellowship to J.S.M.), the Australian Government (PhD scholarship to M.J.B.), and the University of Melbourne Department of Medicine, Dentistry, and Health Sciences (PhD top-up award to M.J.B.). The MSP2 phase 1 vaccine trial was funded by PATH Malaria Vaccine Initiative. The funders had no role in the study design, data collection and analysis, decision to publish, or preparation of the manuscript. We thank D. Wilson and P. Jagannathan for critical reading of the manuscript and Kristina Persson for helpful discussions. This paper is published with permission from the director of KEMRI.

Received: July 6, 2014
Revised: December 1, 2014
Accepted: February 23, 2015
Published: March 17, 2015

REFERENCES

Beeson, J.G., Fowkes, F.J., Reiling, L., Osier, F.H., Drew, D.R., and Brown, G.V. (2014). Correlates of protection for Plasmodium falciparum malaria vaccine development: current knowledge and future research. In Malaria Vaccine Development: Over 40 Years of Trials and Tribulations, G. Corradin and H. Engers, eds. (Future Medicine), pp. 80–104.

Biryukov, S., and Stoute, J.A. (2014). Complement activation in malaria: friend or foe? Trends Mol. Med. *20*, 293–301.

Blackman, M.J., Scott-Finnigan, T.J., Shai, S., and Holder, A.A. (1994). Antibodies inhibit the protease-mediated processing of a malaria merozoite surface protein. J. Exp. Med. *180*, 389–393.

Boyle, M.J., Richards, J.S., Gilson, P.R., Chai, W., and Beeson, J.G. (2010a). Interactions with heparin-like molecules during erythrocyte invasion by Plasmodium falciparum merozoites. Blood *115*, 4559–4568.

Boyle, M.J., Wilson, D.W., Richards, J.S., Riglar, D.T., Tetteh, K.K.A., Conway, D.J., Ralph, S.A., Baum, J., and Beeson, J.G. (2010b). Isolation of viable Plasmodium falciparum merozoites to define erythrocyte invasion events and advance vaccine and drug development. Proc. Natl. Acad. Sci. USA *107*, 14378–14383.

Boyle, M.J., Langer, C., Chan, J.A., Hodder, A.N., Coppel, R.L., Anders, R.F., and Beeson, J.G. (2014). Sequential processing of merozoite surface proteins during and after erythrocyte invasion by Plasmodium falciparum. Infect. Immun. *82*, 924–936.

Cohen, S., McGREGOR, I.A., and Carrington, S. (1961). Gamma-globulin and acquired immunity to human malaria. Nature *192*, 733–737.

Courtin, D., Oesterholt, M., Huismans, H., Kusi, K., Milet, J., Badaut, C., Gaye, O., Roeffen, W., Remarque, E.J., Sauerwein, R., et al. (2009). The quantity and quality of African children's IgG responses to merozoite surface antigens reflect protection against Plasmodium falciparum malaria. PLoS ONE *4*, e7590.

Cowan, G.J.M., Creasey, A.M., Dhanasarnsombut, K., Thomas, A.W., Remarque, E.J., and Cavanagh, D.R. (2011). A malaria vaccine based on the polymorphic block 2 region of MSP-1 that elicits a broad serotype-spanning immune response. PLoS ONE *6*, e26616.

Crompton, P.D., Miura, K., Traore, B., Kayentao, K., Ongoiba, A., Weiss, G., Doumbo, S., Doumtabe, D., Kone, Y., Huang, C.-Y., et al. (2010). In vitro growth-inhibitory activity and malaria risk in a cohort study in mali. Infect. Immun. *78*, 737–745.

Dent, A.E., Bergmann-Leitner, E.S., Wilson, D.W., Tisch, D.J., Kimmel, R., Vulule, J., Sumba, P.O., Beeson, J.G., Angov, E., Moormann, A.M., and Kazura, J.W. (2008). Antibody-mediated growth inhibition of Plasmodium falciparum: relationship to age and protection from parasitemia in Kenyan children and adults. PLoS ONE *3*, e3557.

Dluzewski, A.R., Ling, I.T., Hopkins, J.M., Grainger, M., Margos, G., Mitchell, G.H., Holder, A.A., and Bannister, L.H. (2008). Formation of the food vacuole in Plasmodium falciparum: a potential role for the 19 kDa fragment of merozoite surface protein 1 (MSP1(19)). PLoS ONE *3*, e3085.

Drew, D.R., Hodder, A.N., Wilson, D.W., Foley, M., Mueller, I., Siba, P.M., Dent, A.E., Cowman, A.F., and Beeson, J.G. (2012). Defining the antigenic diversity of Plasmodium falciparum apical membrane antigen 1 and the requirements for a multi-allele vaccine against malaria. PLoS ONE *7*, e51023.

Feng, J.Q., Mozdzanowska, K., and Gerhard, W. (2002). Complement component C1q enhances the biological activity of influenza virus hemagglutinin-specific antibodies depending on their fine antigen specificity and heavy-chain isotype. J. Virol. *76*, 1369–1378.

Flueck, C., Frank, G., Smith, T., Jafarshad, A., Nebie, I., Sirima, S.B., Olugbile, S., Alonso, P., Tanner, M., Druilhe, P., et al. (2009). Evaluation of two long synthetic merozoite surface protein 2 peptides as malaria vaccine candidates. Vaccine *27*, 2653–2661.

Fowkes, F.J.I., Richards, J.S., Simpson, J.A., and Beeson, J.G. (2010). The relationship between anti-merozoite antibodies and incidence of Plasmodium falciparum malaria: A systematic review and meta-analysis. PLoS Med. *7*, e1000218.

Galamo, C.D., Jafarshad, A., Blanc, C., and Druilhe, P. (2009). Anti-MSP1 block 2 antibodies are effective at parasite killing in an allele-specific manner by monocyte-mediated antibody-dependent cellular inhibition. J. Infect. Dis. *199*, 1151–1154.

Genton, B., Betuela, I., Felger, I., Al-Yaman, F., Anders, R.F., Saul, A., Rare, L., Baisor, M., Lorry, K., Brown, G.V., et al. (2002). A recombinant blood-stage malaria vaccine reduces Plasmodium falciparum density and exerts selective pressure on parasite populations in a phase 1-2b trial in Papua New Guinea. J. Infect. Dis. *185*, 820–827.

Hessell, A.J., Hangartner, L., Hunter, M., Havenith, C.E.G., Beurskens, F.J., Bakker, J.M., Lanigan, C.M.S., Landucci, G., Forthal, D.N., Parren, P.W.H.I., et al. (2007). Fc receptor but not complement binding is important in antibody protection against HIV. Nature *449*, 101–104.

Hodder, A.N., Crewther, P.E., and Anders, R.F. (2001). Specificity of the protective antibody response to apical membrane antigen 1. Infect. Immun. *69*, 3286–3294.

John, C.C., O'Donnell, R.A., Sumba, P.O., Moormann, A.M., de Koning-Ward, T.F., King, C.L., Kazura, J.W., and Crabb, B.S. (2004). Evidence that invasion-inhibitory antibodies specific for the 19-kDa fragment of merozoite surface protein-1 (MSP-1 19) can play a protective role against blood-stage Plasmodium falciparum infection in individuals in a malaria endemic area of Africa. J. Immunol. *173*, 666–672.

Lambris, J.D., Ricklin, D., and Geisbrecht, B.V. (2008). Complement evasion by human pathogens. Nat. Rev. Microbiol. *6*, 132–142.

Marsh, K., Otoo, L., Hayes, R.J., Carson, D.C., and Greenwood, B.M. (1989). Antibodies to blood stage antigens of Plasmodium falciparum in rural Gambians and their relation to protection against infection. Trans. R. Soc. Trop. Med. Hyg. *83*, 293–303.

McCallum, F.J., Persson, K.E.M., Mugyenyi, C.K., Fowkes, F.J.I., Simpson, J.A., Richards, J.S., Williams, T.N., Marsh, K., and Beeson, J.G. (2008). Acquisition of growth-inhibitory antibodies against blood-stage Plasmodium falciparum. PLoS ONE *3*, e3571.

McCarthy, J.S., Marjason, J., Elliott, S., Fahey, P., Bang, G., Malkin, E., Tierney, E., Aked-Hurditch, H., Adda, C., Cross, N., et al. (2011). A phase 1 trial of MSP2-C1, a blood-stage malaria vaccine containing 2 isoforms of MSP2 formulated with Montanide® ISA 720. PLoS ONE *6*, e24413.

Mehlhop, E., Nelson, S., Jost, C.A., Gorlatov, S., Johnson, S., Fremont, D.H., Diamond, M.S., and Pierson, T.C. (2009). Complement protein C1q reduces the stoichiometric threshold for antibody-mediated neutralization of West Nile virus. Cell Host Microbe *6*, 381–391.

Michon, P., Cole-Tobian, J.L., Dabod, E., Schoepflin, S., Igu, J., Susapu, M., Tarongka, N., Zimmerman, P.A., Reeder, J.C., Beeson, J.G., et al. (2007). The risk of malarial infections and disease in Papua New Guinean children. Am. J. Trop. Med. Hyg. *76*, 997–1008.

Miura, K., Zhou, H., Diouf, A., Moretz, S.E., Fay, M.P., Miller, L.H., Martin, L.B., Pierce, M.A., Ellis, R.D., Mullen, G.E.D., and Long, C.A. (2009). Anti-apical-membrane-antigen-1 antibody is more effective than anti-42-kilodalton-merozoite-surface-protein-1 antibody in inhibiting plasmodium falciparum growth,

as determined by the in vitro growth inhibition assay. Clin. Vaccine Immunol. *16*, 963–968.

Ndungu, F.M., Bull, P.C., Ross, A., Lowe, B.S., Kabiru, E., and Marsh, K. (2002). Naturally acquired immunoglobulin (Ig)G subclass antibodies to crude asexual Plasmodium falciparum lysates: evidence for association with protection for IgG1 and disease for IgG2. Parasite Immunol. *24*, 77–82.

Nebie, I., Diarra, A., Ouedraogo, A., Soulama, I., Bougouma, E.C., Tiono, A.B., Konate, A.T., Chilengi, R., Theisen, M., Dodoo, D., et al. (2008). Humoral responses to Plasmodium falciparum blood-stage antigens and association with incidence of clinical malaria in children living in an area of seasonal malaria transmission in Burkina Faso, West Africa. Infect. Immun. *76*, 759–766.

Oeuvray, C., Bouharoun-Tayoun, H., Gras-Masse, H., Bottius, E., Kaidoh, T., Aikawa, M., Filgueira, M.C., Tartar, A., and Druilhe, P. (1994). Merozoite surface protein-3: a malaria protein inducing antibodies that promote Plasmodium falciparum killing by cooperation with blood monocytes. Blood *84*, 1594–1602.

Ogutu, B.R., Apollo, O.J., McKinney, D., Okoth, W., Siangla, J., Dubovsky, F., Tucker, K., Waitumbi, J.N., Diggs, C., Wittes, J., et al.; MSP-1 Malaria Vaccine Working Group (2009). Blood stage malaria vaccine eliciting high antigen-specific antibody concentrations confers no protection to young children in Western Kenya. PLoS ONE *4*, e4708.

Persson, K.E.M., Lee, C.T., Marsh, K., and Beeson, J.G. (2006). Development and optimization of high-throughput methods to measure Plasmodium falciparum-specific growth inhibitory antibodies. J. Clin. Microbiol. *44*, 1665–1673.

Polley, S.D., Conway, D.J., Cavanagh, D.R., McBride, J.S., Lowe, B.S., Williams, T.N., Mwangi, T.W., and Marsh, K. (2006). High levels of serum antibodies to merozoite surface protein 2 of Plasmodium falciparum are associated with reduced risk of clinical malaria in coastal Kenya. Vaccine *24*, 4233–4246.

Reiling, L., Richards, J.S., Fowkes, F.J.I., Barry, A.E., Triglia, T., Chokejindachai, W., Michon, P., Tavul, L., Siba, P.M., Cowman, A.F., et al. (2010). Evidence that the erythrocyte invasion ligand PfRh2 is a target of protective immunity against Plasmodium falciparum malaria. J. Immunol. *185*, 6157–6167.

Reiling, L., Richards, J.S., Fowkes, F.J.I., Wilson, D.W., Chokejindachai, W., Barry, A.E., Tham, W.-H., Stubbs, J., Langer, C., Donelson, J., et al. (2012). The Plasmodium falciparum erythrocyte invasion ligand Pfrh4 as a target of functional and protective human antibodies against malaria. PLoS ONE *7*, e45253.

Richards, J.S., Stanisic, D.I., Fowkes, F.J.I., Tavul, L., Dabod, E., Thompson, J.K., Kumar, S., Chitnis, C.E., Narum, D.L., Michon, P., et al. (2010). Association between naturally acquired antibodies to erythrocyte-binding antigens of Plasmodium falciparum and protection from malaria and high-density parasitemia. Clin. Infect. Dis. *51*, e50–e60.

Roussilhon, C., Oeuvray, C., Müller-Graf, C., Tall, A., Rogier, C., Trape, J.-F., Theisen, M., Balde, A., Pérignon, J.-L., and Druilhe, P. (2007). Long-term clinical protection from falciparum malaria is strongly associated with IgG3 antibodies to merozoite surface protein 3. PLoS Med. *4*, e320.

Shi, Y.P., Udhayakumar, V., Oloo, A.J., Nahlen, B.L., and Lal, A.A. (1999). Differential effect and interaction of monocytes, hyperimmune sera, and immunoglobulin G on the growth of asexual stage Plasmodium falciparum parasites. Am. J. Trop. Med. Hyg. *60*, 135–141.

Silver, K.L., Higgins, S.J., McDonald, C.R., and Kain, K.C. (2010). Complement driven innate immune response to malaria: fuelling severe malarial diseases. Cell. Microbiol. *12*, 1036–1045.

Sirima, S.B., Cousens, S., and Druilhe, P. (2011). Protection against malaria by MSP3 candidate vaccine. N. Engl. J. Med. *365*, 1062–1064.

Stanisic, D.I., Richards, J.S., McCallum, F.J., Michon, P., King, C.L., Schoepflin, S., Gilson, P.R., Murphy, V.J., Anders, R.F., Mueller, I., and Beeson, J.G. (2009). Immunoglobulin G subclass-specific responses against Plasmodium falciparum merozoite antigens are associated with control of parasitemia and protection from symptomatic illness. Infect. Immun. *77*, 1165–1174.

Stubbs, J., Olugbile, S., Saidou, B., Simpore, J., Corradin, G., and Lanzavecchia, A. (2011). Strain-transcending Fc-dependent killing of Plasmodium falciparum by merozoite surface protein 2 allele-specific human antibodies. Infect. Immun. *79*, 1143–1152.

Taylor, R.R., Allen, S.J., Greenwood, B.M., and Riley, E.M. (1998). IgG3 antibodies to Plasmodium falciparum merozoite surface protein 2 (MSP2): increasing prevalence with age and association with clinical immunity to malaria. Am. J. Trop. Med. Hyg. *58*, 406–413.

Wilson, D.W., Crabb, B.S., and Beeson, J.G. (2010). Development of fluorescent Plasmodium falciparum for in vitro growth inhibition assays. Malar. J. *9*, 152.

Wilson, D.W., Fowkes, F.J.I., Gilson, P.R., Elliott, S.R., Tavul, L., Michon, P., Dabod, E., Siba, P.M., Mueller, I., Crabb, B.S., and Beeson, J.G. (2011). Quantifying the importance of MSP1-19 as a target of growth-inhibitory and protective antibodies against Plasmodium falciparum in humans. PLoS ONE *6*, e27705.

Immunity

Supplemental Information

Human Antibodies Fix Complement to Inhibit *Plasmodium falciparum* Invasion of Erythrocytes and Are Associated with Protection against Malaria

Michelle J. Boyle, Linda Reiling, Gaoqian Feng, Christine Langer, Faith H. Osier, Harvey Aspeling-Jones, Yik Sheng Cheng, Janine Stubbs, Kevin K.A. Tetteh, David J. Conway, James S. McCarthy, Ivo Muller, Kevin Marsh, Robin F. Anders, and James G. Beeson

Supplementary Data

Supplementary Figure 1:

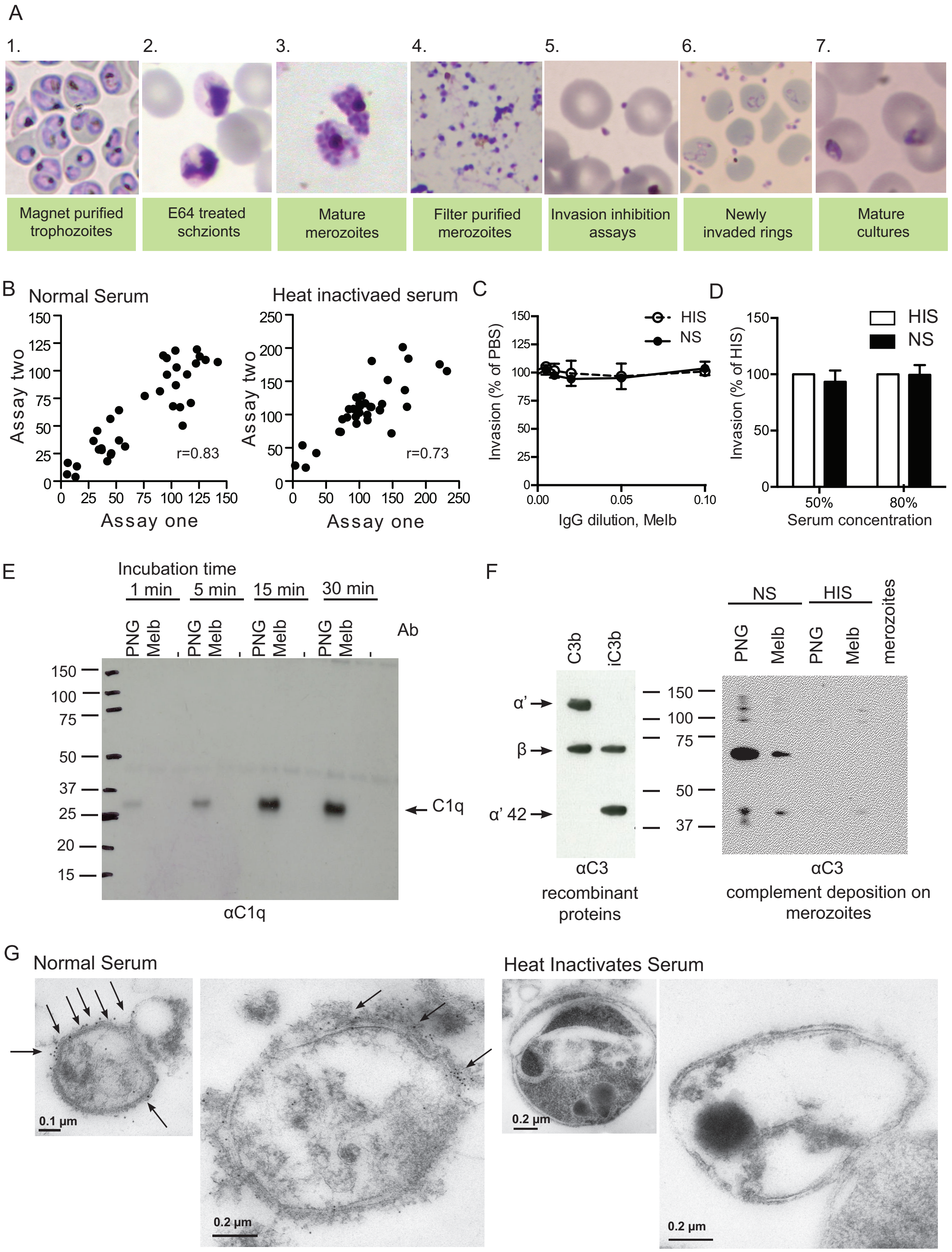

Supplementary Figure S1: Invasion inhibition assays and complement deposition on merozoites (see also Figure 1).

A. Procedure for isolation of merozoites and invasion inhibition assays – refer to Supplementary Experimental Procedures; 1) synchronized mature parasites are purified via MACs separation. 2) Cultures are matured to segmented schizonts and treated with E64. 3) Mature merozoites are formed within E64 treated schizonts. 4) Merozoites are isolated via membrane filtration. 5) Isolated merozoites are incubated with uninfected RBCs, normal or heat-inactivated serum and purified IgG for 30 minutes in invasion inhibition assays. 6) Invaded rings are washed and returned to standard culture conditions. 7) Parasites are cultured in standard conditions until mature and enumerated via flow-cytometry. Figure is modified from (Boyle et al., 2013). **B.** Invasion inhibition assays with Normal Serum (NS) and Heat-inactivated serum (HIS) are reproducible; purified IgG from 33 Kenyan donors were tested with 50% NS and 50% HIS in invasion inhibition assays on two separate experimental days. Assays were reproducible; Spearman's rho was 0.83 for inhibition with NS (95% CI; 0.69 0.92, $p<0.001$) and $r=0.73$ for inhibition in HIS (95% CI; 0.51 0.86, $p<0.001$). **C.** IgG from unexposed donors has no inhibitory activity with NS or HIS; Purified merozoites were allowed to invade RBCs in the presence of 50% NS or HIS with IgG from serum pools from residents in Melbourne, Australia (data are mean ± range of two assays in duplicate). **D.** Complement alone does not inhibit invasion; Purified merozoites were allowed to invade RBCs in the presence of normal serum (NS) or heat-inactivated serum (HIS) at 50% and 80% final serum concentrations (data is mean ± SEM. of four assays in duplicate). **E.** Specificity of C1q antibodies used in complement deposition assays; Entire western blot of C1q deposition on merozoites from Figure 1b, indicating that anti-C1q antibody is specific, and reacts with a single protein of approximately 30kDa, consistent with C1q. **F.** Specificity of C3 antibodies and reactivity to C3b and iC3b; Right panel shows C3 antibody reactivity with recombinant C3b and iC3b, with arrows indicating α and β chains. Left panel shows complement deposition on merozoites incubated with IgG from PNG or Melbourne donors, incubated with normal serum (NS) or heat-inactivated serum (HIS). **G.** Deposition of C3b on merozoites by immune-electron microscopy; Two example images of C3b deposition on the merozoite surface with merozoites incubated with PNG IgG and normal serum (left panels), and the lack of C3b deposition on merozoites incubated with PNG IgG and heat-inactivated serum (right panels).

Supplementary Figure 2:

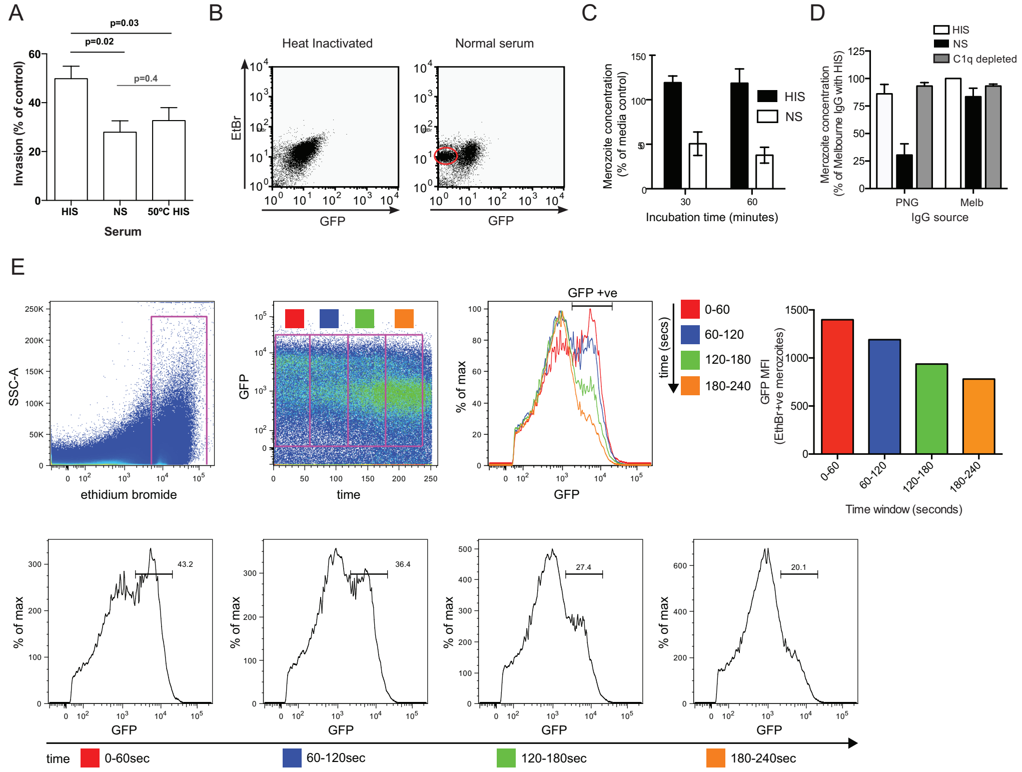

Supplementary Figure S2: Importance of different complement components in mediating inhibition (see also Figure 2).

A. Alternative complement pathway is less important than classical complement pathway; Invasion inhibition activity of PNG IgG was tested in 25% serum that heat-inactivated serum at 56°C for 30 minutes (HIS), normal serum (NS) and serum heat-treated at 50°C for 20 minute (50°C HIS). Heat-inactivation at 56°C for 30 minutes disrupts all complement activation cascades, while heat-treatment at 50°C for 20 minutes disrupts only the alternative complement cascade by inactivation of Factor B, while leaving the classical complement cascade intact. The 50°C treatment of serum had no significant effect on the invasion-enhancing activity of serum; invasion-inhibition by PNG IgG was significantly greater in the presence of NS and 50°C-treated serum, compared to HIS. **B.** Analysis of merozoite lysis; Merozoites were incubated with HIS or NS and lysis monitored via flow cytometry by assessing loss of cytosolyic GFP expression by *P. falciparum* merozoites. In NS, merozoites lost GFP expression as indicated by the red circle. Intact merozoites were counted with count bright counting beads by gating on EtBr positive and GFP positive merozoites. **C.** Merozoite lysis occurs in the absence of IgG in extended incubations; Merozoites were incubated for 30 or 60 minutes with NS and HIS in the absence of IgG. Merozoite lysis was evident after prolonged incubation, indicating that over extended time periods lysis of merozoites did

occur, likely mediated by the activation of alternative complement pathways on the merozoite surface. **D.** Rapid lysis of merozoites requires C1q; Merozoites were incubated for 10 minutes with NS or HIS or C1q-depleted serum together with IgG from PNG or Melbourne, Australian donors. Merozoite lysis after ten minutes of incubation was only observed in NS with IgG from PNG donors, indicating that lysis was dependent on the activation of the classical complement cascade by merozoite specific IgG. **E.** Gating strategies for assessing the rate of merozoite lysis; To assess the rate of lysis of merozoites incubated with NS and malaria-specific IgG, merozoites were incubated at 37°C with PNG IgG and NS and monitored via flow-cytometry during lysis. Merozoites were gated on EtBr positive cells, and then GFP MFI was monitored. GFP positive cells were rapidly lost within 1-4 minutes of incubation, as indicated by the loss of positive GPF cells and overall decreased MFI of merozoites.

Supplementary Figure 3:

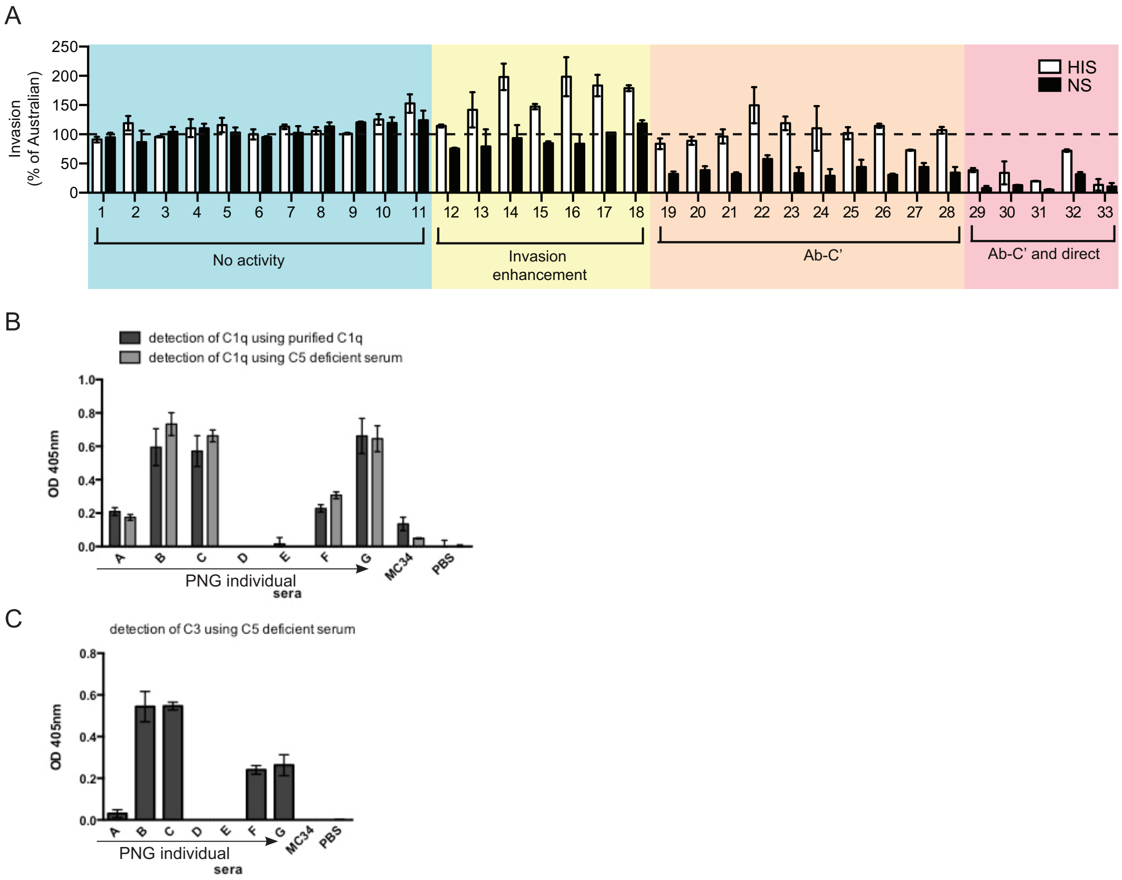

Supplementary Figure S3: Ab-C' inhibition in individuals and fixation of complement components on merozoites (see also Figure 3).

A. Ab-C' is the predominant mechanism of invasion-inhibition in naturally acquired antibodies; Invasion inhibition activity profiles of purified IgG from all 33 Kenyan individuals in the presence of NS and HIS; no inhibition activity (blue), invasion enhancement activity (in HIS and not NS) (yellow), Ab-C' inhibition (orange) and Ab-C' and direct inhibitory activity (red) (data are mean ± range of two assays in duplicate). Sample numbers do not necessarily correspond with those represented in Figure 3b. **B.** Quantification of C1q deposition with PNG individuals using C1q and C5-deficient serum on merozoite; C1q-fixation on the merozoite surface by IgG from PNG individuals (A-G), IgG from Melbourne donors (MC34) or control (PBS). Either purified C1q or C5-deficient serum was used as the source of C1q in the assays. **C.** Quantification of C3b deposition with PNG individuals using C5-deficient serum on merozoites; C3b-fixation on the merozoite surface by IgG from PNG individuals (A-G), IgG from Melbourne donors (MC34) or control (PBS). C5-deficient serum was used as the source of complement in the assays.

Supplementary Figure 4:

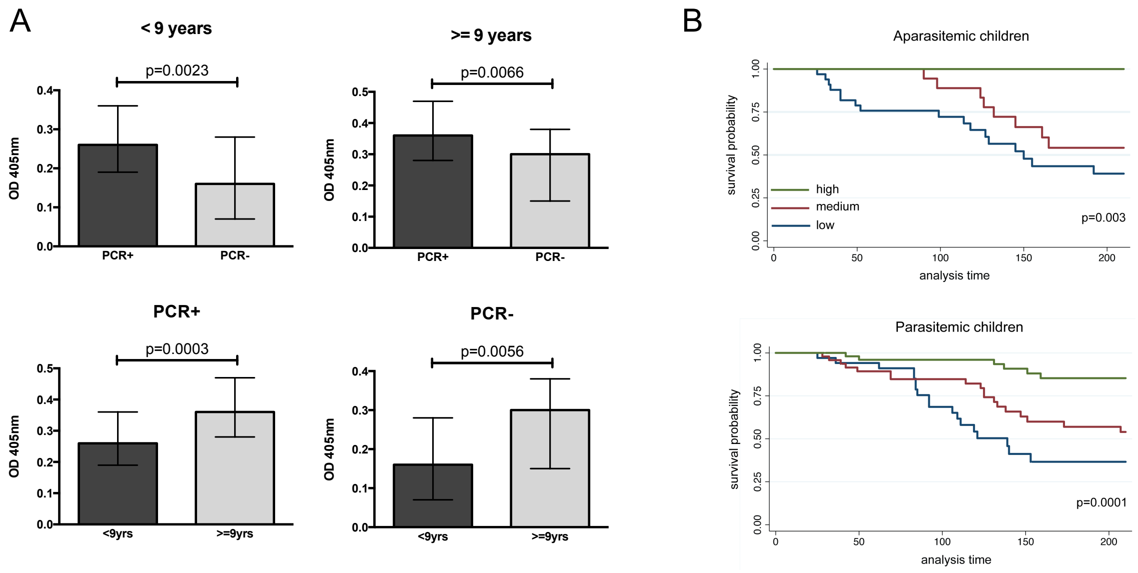

Supplementary Figure S4: Analysis of complement fixation in the children's cohort stratified by age and parasite status (see also Figure 4).

A. C1q fixation by children; C1q fixation (median OD) by children's samples was stratified by parasitemia status at enrolment (determined by PCR) and age. C1q fixation was significantly higher in the presence of parasitemia among older and younger children. Older children had greater C1q fixation than younger children regardless of whether there was active parasitemia. **B.** Association between complement fixing antibodies and clinical malaria; Kaplan Meier survival curves for time to first clinical episode of malaria with children stratified into the groups of high, medium and low C1q fixation. Survival curves are shown for children stratified by parasitemia status at time of enrolment; all children were subsequently treated to clear parasitemia prior to follow-up (p=0.003 for aparasitemic children, and p=0.0001 for parasite positive children, comparing high medium and low responder groups.)

Supplementary Figure 5:

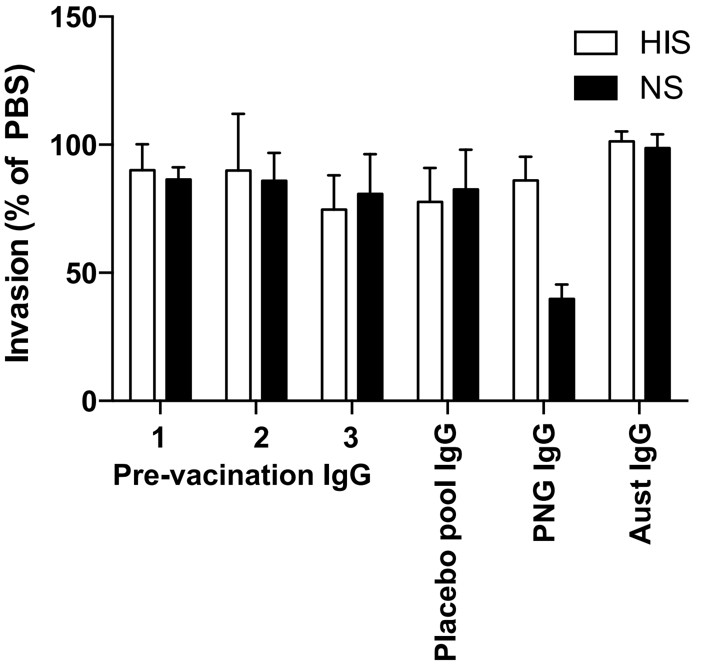

Supplementary Figure S5: Pre-vaccination samples and placebo vaccine serum pools in invasion inhibition assays (see also Figure 5).

Purified IgG from individuals prior to vaccination and from day 120 post vaccination placebo sample pools were tested in invasion inhibition assays with normal serum (NS) and heat-inactivated serum (HIS). Data represent two assays in duplicate and normalized to invasion with Australian IgG. PNG IgG and Australian IgG were used as positive and negative controls respectively.

Supplementary Table S1: Prevalence and levels of antibody-mediated C1q deposition on the merozoite surface in the cohort of children (related to table Table 2).

		Age [4]			Enrolment P. falciparum parasitemic status [5]		
	**All n =200 [7]**	**≤ 9yrs n=90**	**> 9yrs n=110**	**p**	**PCR- n=65**	**PCR+ n=135**	**p**
**Seropositive[1] %[2]**	198 99%	88 97.8%	110 100%	0.116	64 98.5%	134 99.3%	0.5
**Median OD [IQR] [3]**	0.30 [0.20-0.41]	0.24 [0.14-0.33]	0.35 [0.26-0.43]	**<0.0001**	0.23 [0.08-0.34]	0.34 [0.23-0.43]	**<0.0001**

[1] Number of individuals from the cohort with C1q deposition on merozoites' surface

[2] %: percent of individuals from the cohort with C1q deposition on merozoites' surface

[3] [IQR]: inter-quartile range

[4] Age: the cohort was stratified by age into two groups: children 9 years of age an younger (≤) or older than 9 years of age

[5] Enrolment *P. falciparum* parasitemic status: PCR- indicates aparasitemic status at enrolment, PCR+ indicates parasitemic status at enrolment, both as determined by PCR

[7] Number of samples is 200 due to insufficient sample volume or excluded values where the discrepancy between duplicates was >25%.

Supplementary Experimental Procedures

Parasite culture and synchronization

The *P. falciparum* D10-GFP expression line (Wilson et al., 2010) was cultured as described (Persson et al., 2006) in culture medium of RPMI-HEPES (pH 7.4) supplemented with 50 μg/ml hypoxanthine, 20 μg/ml gentamicin, 25 mM sodium bicarbonate and 10% (volume/volume) pooled human serum from unexposed Melbourne, Australia blood donors (supplied by the Australian Red Cross Blood service). RBCs from group O+ blood donors (supplied by the Australian Red Cross Blood service) were used to culture parasites. Typically cultures were maintained at 3% hematocrit, with a parasitemia of 1-5%. Cultures were maintained in 1% O_2, 4% CO_2, 95% N_2 and incubated at 37°C. For monitoring of cultures, blood slides were stained with 10% Giemsa (Merck).

Parasites were synchronized using 5% D-sorbitol-treatment; cultures were incubated for 5 minutes with 5% D-sorbitol, pelleted and washed one time with culture media before returning to culture. Cultures were further synchronized using the invasion inhibitory properties of heparin (Boyle et al., 2010a; Wilson et al., 2010). Parasites were cultured in the presence of 30IU (approximately 230 μg/ml) of medical grade heparin (Porcine mucous, Pfizer) until the majority of parasites were at the schizont stage. Under heparin inhibition, invasion of RBCs by merozoites rupturing from schizonts was inhibited. Heparin was then removed from cultures by centrifugation of the culture and re-suspension of cells in fresh culture medium for 4-6 hours allowing merozoite invasion and the development of ring stages in RBCs.

Invasion inhibition assays

Invasion inhibition assays were performed with isolated viable merozoites as described (Boyle et al., 2013; 2010b) (Figure S1). A detailed method for the isolation for viable merozoites and invasion inhibition assay can be found at Methods in Malaria Research, http://www.mr4.org/Publications/MethodsinMalariaResearch.aspx. Late stage trophozoites were magnet purified on MACs purification column to remove uninfected RBCs. Following purification, parasites were incubated in standard culture media with

10μM of trans –Epoxysuccinyl-L-leucylamido(4-guanidino)butane (E64) cysteine protease inhibitor for 6-8 hours. This cysteine protease inhibitor allows for the maturation of merozoites but prevents schizont rupture (Blackman, 2008; Boyle et al., 2010b; Glushakova et al., 2009). Following incubation, mature merozoites were formed and isolated via membrane filtration in a 1.2 μm filter; cultures were washed once to remove E64 and resuspended in serum free culture media in a volume required for invasion inhibition assays (for example, if 1ml of isolated merozoites was required, E64 treated cultures were resuspended in 1.5ml of media prior to filtration). Isolated merozoites were then added to uninfected RBCs (final concentration 0.5% haematocrit), test IgG (typically at 1/10 dilution or as indicated in results) and normal serum (NS, complement active – concentrations indicated in results) or heat inactivated serum (HIS, complement in-active – concentrations indicated in results) in a 96 well plate in 50μl suspensions. Cultures were incubated for 10 minutes with agitation on a plate shaker at 500rpm and then transferred to a gassed chamber (standard culture gas conditions) for a further 20 minutes of incubations, allowing for a total invasion time of 30 minute. A 30 minute incubation period was chosen as prior published data indicates that merozoite invasion occurs rapidly, with 80% of invasion occurs within 10 minutes of merozoite contact with RBCs. However, a further 20% of invasion occurs after 10minutes (Boyle et al., 2010b), and it is possible that *in vivo* sequestration of parasites would require free merozoites to make their way to areas of the blood stream where RBCs are available (Boyle et al., 2013). Cultures were then washed twice with incomplete culture media (lacking human serum) and then once with standard culture media to remove IgG and NS and HIS and a proportion of uninvaded merozoites. Cultures were then returned to culture in standard culture media for invaded parasite to mature. Cultures were maintained for 40 hours and then analysed by flow cytometry as described (Boyle et al., 2010b; Wilson et al., 2010). Analysis was performed at 40 hours post invasion due to ease of gating. Invasion assays were reproducible, as measured by Spearman's correlations of two independent assays; for inhibition in NS, correlation coefficient was 0.83 (95% CI; 0.69 0.92, $p<0.001$) and for inhibition in HIS $r=0.73$ (95% CI; 0.51 0.86, $p<0.001$) (Figure S2).

Complement-active serum as the source for invasion inhibition assays (normal serum; NS) was collected from malaria-naïve (Australian) donors; blood was collected

without anti-coagulants and allowed to clot for 1-2 hours at room temperature. RBCs were pelleted at 2800 rpm for 10 minutes and serum aliquoted and stored at -80°C. Serum was thawed once only for use in assays. For heat-inactivation, serum was thawed and heated at 56°C for 30 minutes, to generate heat-inactivated serum (HIS) lacking complement activity. To test for the importance of alternative pathway amplification in Ab-C' activity, serum was heat-inactivated at 50°C for 20 minutes, which disrupts Factor B and the alternative pathway, but does not affect classical pathway activation. For assays testing the importance of C1q and C5 for Ab-C' activity, human serum depleted of complement factors C1q or C5 and purified human C1q and C5 were purchased from Calbiochem, Merck. Due to reagent limitations, a final serum concentration of 25% was used. Ab-C' inhibition activity of PNG IgG at 1:20 dilution was comparable in NS at 25% or 50% concentration.

Growth inhibition assays

Growth inhibition assays were performed as described previously (McCallum et al., 2008; Persson et al., 2006; Wilson et al., 2010). Detailed methods for growth inhibition assays can be found at http://www.mr4.org/Publications/MethodsinMalariaResearch.aspx. Duplicated suspensions of sorbitol synchronized parasites at 0.2% parasitemia, 1% hematocrite were incubated with 10% plasma collected in XYZ tubes from donors in 96-well sterile U-bottom plates (Falcon). Plasma was dialyzed as described (Persson et al., 2006), via 50kDa cut-off Tube-O-Dialyzer in PBS for 2 hours at 4°C. Following dialysis, samples were concentrated in 100kDa cut-off Nanosep spin tubes and restored to original volumes with PBS. Plates were incubated as for parasite culture for 72 hours. At 48 hour time point 5ul of fresh media was added to supplement cultures. After 72 hours, parasites were stained with ethidium bromide and analysed via flow cytometry as described for invasion inhibition assays. The use of flow cytometry for analysis of growth inhibitory assays has been shown to highly replicable (Persson et al., 2006; Wilson et al., 2010).

Human subjects and samples

Ethical approval for the use of human serum and plasma samples in these studies was obtained from the Alfred Human Research and Ethics Committee (for the Burnet Institute), Kenya Medical Research Institute, Medical Research Advisory Committee of Papua New Guinea, and the Human Research and Ethics Committee of the Queensland Institute of Medical Research. Written informed consent was obtained from all participants, or their parents/guardians (in the case of children).

Serum pools from malaria-exposed adults were made from individual serum samples from two geographically distinct regions; Ngerenya pool from Kilifi District, Kenya, and PNG pool from Madang district, Papua New Guinea. Ngerenya samples were collected in a cross sectional survey conducted in 1998 in the Kilifi district in an area of low transmission (Mwangi et al., 2005). For the Ngerenya pool, 33 individual serums samples (age 1 – 68 years) were screened for reactivity to merozoite antigens by ELISA, and the pool made from 27 positive individuals. PNG serum was pooled from 26 individuals (men and women) taken during a cross sectional study performed in Modilon Hospital, Madang and Yagaum Health Centre PNG in 2001/2002 (Beeson et al., 2007). Unexposed serum pools were from Melbourne, Australia donors from Red Cross Blood bank supplies. IgG from serum pools was Melon Gel purified (Thermo Scientific), as per manufacturer's instructions. 500 μl of Melon Gel purification support matrix was equilibrated to room temperature and washed two times with purification buffer though spin columns. Serum was diluted 1:10 with purification buffer. Diluted serum was incubated for five minutes on purification support matrix and the unbound (antibody) fraction was collected in flow through. Diluted purified IgG was concentrated in 10 kDa MWC spin purification tubes (Amicon) and purification buffer was exchanged with PBS to a concentration of < 0.8%. The concentrations of IgG purified from the malaria-exposed and malaria-naïve serum were comparable (data not shown).

Serum samples were used from a Phase 1 vaccine trial of MSP2-C1 where vaccinees were immunised with both 3D7 and FC27 isoforms of MSP2 formulated with Montanide® ISA 720 as described (McCarthy et al., 2011) (sponsored by PATH Malaria Vaccine Initiative; Trial Registration, Australian New Zealand Clinical Trials Registry 12607000552482) . Vaccinated adults were resident of Brisbane, Australia, not from a

malaria endemic country, and had not travelled to a malaria endemic country in the two years prior to vaccination.

For the longitudinal study of Papua New Guinean children, plasma samples were obtained at enrolment from a prospective treatment-reinfection cohort of 206 children aged 5-14 years (median=9.3) in Madang, PNG (Michon et al., 2007). At enrolment the prevalence of was 67.5% (n= 139) by PCR and 40.3% (n= 83) by light microscopy (the geometric mean parasite density was 361 parasites/ml (95% CI, 240–544). After enrolment, all children received 7 days of artesunate orally to clear parasitemia; treatment failures were differentiated from re-infection by genotyping of msp2. Children were actively reviewed every 2 weeks for symptomatic illness and parasitemia by PCR and microscopy, and by passive case detection, over a period of 6 months. A clinical episode of *P. falciparum* malaria was defined as fever and *P. falciparum* parasitemia >5000/ parasites/µl. At enrolment, 21.8% of children had mixed *P. falciparum* and *P. vivax* infections, and 82% of children had PCR-detectable *P. vivax* blood-stage infection during the course of follow-up (Michon et al., 2007). In data analysis of the relationship between antibodies and *P. falciparum* infection, we included all *P. falciparum* infections. In the analysis of associations between antibodies and risk of malaria, we included *P. falciparum* malaria cases only (during follow-up there were very few malaria episodes due to other *Plasmodium* species). We have previously found no relationship between antibodies to *P. falciparum* and *P. vivax* infection, or between *P. vivax* antibodies and *P. falciparum* infection ((Cole-Tobian et al., 2009); and unpublished observations). Additionally, in a prior study on the Thailand-Myanmar border, we similarly found no clear effect of *P. vivax* infection on *P. falciparum* antibodies (Fowkes et al., 2012).

MSP2 and MSP3 human antibodies

Human antibodies to MSP2 (FC27) from naturally exposed individuals were affinity-purified from a serum pool of PNG residents by column chromatography using the two allelic isoforms of MSP2 both as full length recombinant proteins, namely MSP2(3D7) and MSP2(FC27), using established methods (Reiling et al., 2012). Consequently, MSP2(FC27) purified IgG contained antibodies directed only to the FC27-

allelic specific region of MSP2. The D10-PfPHG parasite strain used in these assays expresses the FC27 allele of MSP2 (confirmed by western blot, data not shown).

Human antibodies against the K1 allelic version of MSP3(Polley et al., 2007) were affinity purified from a 50ml pool of plasma taken from malaria semi-immune adults in Kenya using CNBr-activated SepharoseTM 4B (GE Healthcare), using established methods (Reiling et al., 2012).

Monoclonal human antibodies targeting MSP2, along with Fc-LALA mutants were isolated and developed as describe (Stubbs et al., 2011).

Rabbit antibodies

Rabbit serum were raised as described; to MSP1-19 (Stanisic et al., 2009) (Brendan Crabb and Paul Gilson, Burnet Institute); to MSP1-block 2 (Boyle et al., 2010b); to full length MSP4 (Wang et al., 1999) (Ross Coppel and Brian Cooke, Monash University), AMA1 (3D7 and 7G8 alleles) polyclonal rabbit serum (Drew et al., 2012) (Damien Drew, Burnet Institute and Anthony Hodder, Walter and Eliza Hall Institute). Rabbit polyclonal antibodies to MSP2 were generated by vaccination C-terminally His-tagged recombinant MSP2 formulated in Montanide ISA720 as used in the phase clinical trail of MSP2-C1 (McCarthy et al., 2011); Rabbit polyclonal serum was raised to the MSP3 C-terminal MBP-tagged conserved region (Polley et al., 2007).

Complement deposition assays on whole merozoites for western blot and microscopy

To test for the deposition of complement on the merozoite surface, isolated merozoites were incubated with 25% NS with IgG from exposed (PNG), or unexposed (Australian) pools, Ngerenya individuals or PBS for 1, 5, 10, 15 or 30 minutes, agitated at 37°C. After incubation, merozoites were pelleted at 3000g for 5 minutes, washed twice with cold PBS containing complete protease inhibitors and then solubilised in reducing sample buffer and processed for western blot. Deposition of C1q and C3 was assessed using antibodies to C1q (Goat polyclonal, Calbiochem, Merck) and C3 (HRP conjugated goat polyclonal, MP Biomedicals). The merozoite surface protein, MSP1-42 fragment was used as a loading control and was detected with polyclonal rabbit serum to MSP1-19.

For immune-electron microscopy isolated merozoites were incubated with normal and heat inactivated serum with PNG IgG for ten minutes. Merozoites were washed twice in PBS containing complete protease inhibitor and fixed in 1% glutaraldehyde in RPMI-HEPES on ice for 30 min. Samples were pelleted in low-melt agarose before being transferred into water, dehydrated in ethanol, and embedded in LR White Resin (ProSciTech). Following polymerization by benzoyl peroxide (SPI-Chem), 100 nm sections were prepared by using an Ultracut R ultramicrotome (Leica). Sections were post-stained with 2% aqueous uranyl-acetate, and observed at 120 kV on a CM120 BioTWIN transmission electron microscope (Philips).

To detect membrane attack complex (MAC) deposition on merozoites, isolated merozoites were incubated with 25% NS serum and IgG from PNG or Australian serum pools for 10 minutes at 37°C. Merozoites were washed twice in cold PBS with protease inhibitors and either dried on slides for IF-microscopy. Slides were fixed with cold methanol for 5 minutes, dried, blocked with 3% BSA and MAC detected with anti-C5-9 antibodies (rabbit), and anti-rabbit-Alexa 488 conjugated antibodies. Slides were mounted in VectaSheild (Vector Laboratories) with 0.1ng/ml 4',6-diamidino-2-phenylindole (DAPI) to label the parasite nucleus. Images were obtained using a Plan-Apochromate (100X/1.40) oil immersion phase-contrast lens (Carl Zeiss) on an AxioCam Mrm camera (Carl Zeiss). Images were processed using Photoshop CS4 (Adobe). In cases where brightness and contrast were changes, processing was applied to whole images and controls equally.

Complement deposition on whole merozoites for ELISA

Plates were coated with purified merozoites (Boyle et al., 2010b)at $5x10^6$ merozoites/well, incubated overnight at 4 degree. Plates were blocked for 2 hours at 37 degree with 1% casein, incubated with sera samples at 1/250 dilution in 0.1% casein for 2 hours at room temperature (RT). For detection and quantification of C1q fixation, plates were incubation with recombinant C1q at 10 μg/ml in 0.1% casein for 30 minutes at RT. C1q deposition was detected with goat anti-C1q antibodies, followed by anti-goat-HRP, each incubated for 1 hour at RT, diluted 1/500 in 0.1% casein. For detection of C1q and C3 fixation using serum as a complement source, plates were incubated with 20% C5-

deficient serum to prevent lysis of merozoites. C3 was detected with anti-C3-HRP (conjugated goat polyclonal, MP Biomedicals), followed by anti-goat-HRP, each incubated for 1 hour at RT, diluted 1/500 in 0.1% casein. To detect membrane attack complex (MAC) deposition on merozoites, isolated merozoites were incubated with 25% NS serum and IgG from PNG or Australian serum pools for 10 minutes at 37°C. Merozoites were washed twice in cold PBS with protease inhibitors and coated into Nunc plates for ELISA. Merozoites were coated overnight at 4°C in PBS containing protease inhibitors. Wells were then blocked with 10% milk in PBS, and MAC deposition detected with anti-C5-9 antibodies.

Enzymatic activity was detected using ABTS liquid substrate with reactions being stopped after 30 minutes to 1 hour with 1% SDS. Each sample was run in duplicate, and samples with a discrepancy of >25% between duplicates were excluded from the analysis. Variations between plates were accounted for by standardizing using positive controls on each plate. Unexposed controls from Melbourne Australia donors were used as negative controls. For each assay we include control wells, which contain no merozoites. These blank wells give an OD value in the range of 0.1-0.26. This value is regarded as background non-specific signal and deducted from test sample readings, and is well below the signal of the strong positive samples or positive controls.

Merozoite lysis assays

Freshly isolated D10-GPF merozoites were added to a final concentration of 5% hyperimmuned PNG serum (VT pool) and 20% fresh/heat-inactivated serum and incubated at 37 degree for 10 minutes. A pool of sera from malaria-naive Melbourne donors was used instead of PNG serum as negative control. Following incubation samples were diluted 1/100 in 200ul cold PBS-1%NCS (new born calf serum) immediately after incubation (quenching complement deposition). The density of merozoites was counted by flow cytometry following the addition of CountBright counting beads. Merozoites were gated as the GFP-positive population and the density was calculated relative to counting beads; merozoite lysis was expressed as the percentage of lysed merozoites compared to the negative control. For assays to assess rate of merozoite lysis, merozoites were incubated as described above and aliquots taken at

one minute intervals for flow cytometry analysis. Data is expressed as the % of maximum lysis that occurs within 10 minutes of incubation.

ELISA to intact merozoites

ELISA were performed using standard methods (Stanisic et al., 2009). Purified merozoites were coated in PBS onto Maxisorb microtitre plates (Nunc) overnight at 4°C. Plates were blocked with 10% milk for 2 hours at 37°C, followed by incubation with Ngerenya serum samples diluted 1:250 in 5% milk at room temperature for two hours. Binding of antibody was detected with polyclonal sheep anti-human IgG HRP 1:2500 5% milk (Chemicon). Binding was detected with ABTS liquid substrate system (Sigma). Reactions were stopped after 20 minutes with 1% sodium dodecyl sulfate (SDS) and optical density measured at 405 nm. Plates were washed five times with PBS following each incubation.

Data analysis

Differences in invasion inhibition activity for IgG in NS compared to HIS and serum C1q depleted and reconstituted serum was calculated with paired t-test in StataSE 11.2. Associations between antibody levels to intact merozoites via ELISA and functional activity in Ab-C', direct inhibition and growth inhibitory assays were assessed with Spearman's correlations calculated in Prism.

Analysis of the cohort study was performed using Stata/SE 12.0 (StataCorp College Station, Texas, USA). Differences in prevalence and levels of C1q deposition between subgroups were assessed by chi-square tests (for categorical variables) or Wilcoxon rank sum tests (for continuous variable). In order to assess associations between C1q deposition and protection, the subjects was stratified into tertiles according to low (including those classified as 'negative'/'no deposition), medium or high deposition of C1q, as determined by OD values for each sample. Groups were compared for the risk of clinical malaria or high-density parasitemia as described previously (Reiling et al., 2010; Richards et al., 2010). Survival analysis included time to first episode only. The cox proportional hazards model was used to calculate hazard ratios for risk of clinical malaria or high-density parasitemia between different tertiles. Age and location of residence were

previously identified as potential confounders from a range of factors (Michon et al., 2007). Although antibodies at baseline were higher in those children with current parasitemia, parasitemia at baseline was not significantly associated with malaria. As such, hazard ratios were only adjusted for by age and location of residence.

Supplementary Reference

Beeson, J.G., Ndungu, F., Persson, K.E.M., Chesson, J.M., Kelly, G.L., Uyoga, S., Hallamore, S.L., Williams, T.N., Reeder, J.C., Brown, G.V., et al. (2007). Antibodies among men and children to placental-binding Plasmodium falciparum-infected erythrocytes that express var2csa. American Journal of Tropical Medicine and Hygiene *77*, 22–28.

Blackman, M.J. (2008). Malarial proteases and host cell egress: an “emerging”cascade. Cell. Microbiol. *10*, 1925–1934.

Boyle, M.J., Wilson, D.W., and Beeson, J.G. (2013). New approaches to studying Plasmodium falciparum merozoite invasion and insights into invasion biology. International Journal for Parasitology *43*, 1–10.

Cole-Tobian, J.L., Michon, P., Biasor, M., Richards, J.S., Beeson, J.G., Mueller, I., and King, C.L. (2009). Strain-specific duffy binding protein antibodies correlate with protection against infection with homologous compared to heterologous plasmodium vivax strains in Papua New Guinean children. Infection and Immunity *77*, 4009–4017.

Fowkes, F.J.I., McGready, R., Cross, N.J., Hommel, M., Simpson, J.A., Elliott, S.R., Richards, J.S., Lackovic, K., Viladpai-Nguen, J., Narum, D., et al. (2012). New insights into acquisition, boosting, and longevity of immunity to malaria in pregnant women. Journal of Infectious Diseases *206*, 1612–1621.

Glushakova, S., Mazar, J., Hohmann-Marriott, M.F., Hama, E., and Zimmerberg, J. (2009). Irreversible effect of cysteine protease inhibitors on the release of malaria parasites from infected erythrocytes. Cell. Microbiol. *11*, 95–105.

Mwangi, T.W., Ross, A., Snow, R.W., and Marsh, K. (2005). Case definitions of clinical malaria under different transmission conditions in Kilifi District, Kenya. J. Infect. Dis. *191*, 1932–1939.

Polley, S.D., Tetteh, K.K.A., Lloyd, J.M., Akpogheneta, O.J., Greenwood, B.M., Bojang, K.A., and Conway, D.J. (2007). Plasmodium falciparum merozoite surface protein 3 is a target of allele-specific immunity and alleles are maintained by natural selection. J. Infect. Dis. *195*, 279–287.

Wang, L., Black, C.G., Marshall, V.M., and Coppel, R.L. (1999). Structural and antigenic properties of merozoite surface protein 4 of Plasmodium falciparum. Infection and
